# A chiral self-sorting photoresponsive coordination cage based on overcrowded alkenes

**DOI:** 10.3762/bjoc.15.268

**Published:** 2019-11-15

**Authors:** Constantin Stuckhardt, Diederik Roke, Wojciech Danowski, Edwin Otten, Sander J Wezenberg, Ben L Feringa

**Affiliations:** 1 Stratingh Institute for Chemistry, University of Groningen, Nijenborgh 4, 9747 AG, Groningen, The Netherlands; 2 Organisch-Chemisches Institut, University of Münster, Corrensstrasse 40, 48149 Münster, Germany; 3 Leiden Institute of Chemistry, Leiden University, Einsteinweg 55, 2333 CC, Leiden, The Netherlands

**Keywords:** coordination cages, molecular motors, molecular switches, overcrowded alkene, palladium

## Abstract

In recent years, increasing efforts have been devoted to designing new functional stimuli-responsive supramolecular assemblies. Here, we present three isomeric supramolecular coordination complexes consisting of a Pd_2_L_4_ stoichiometry. As shown by NMR, CD and X-ray studies, as well as DFT calculations, these complexes form cage-like structures by chiral self-sorting. Photochromic ligands derived from first generation molecular motors enable light-driven interconversion between the three isomers. Two of the isomers were able to form host–guest complexes opening up new prospects toward stimuli-controlled substrate binding and release.

## Introduction

Supramolecular coordination complexes (SCCs) represent a promising class of compounds which have been used in a variety of molecular systems [1–6]. Taking advantage of the vacant cavity inside these complexes, SCCs have been applied in drug delivery [6–8], supramolecular catalysis [9–12], X-ray structure determination [13–14] and stabilization of reactive species [15–17]. The use of reversible metal–ligand coordination bonds gives rise to systems that allow for adaption to external stimuli such as pH, anions, electric potential, concentration and light [4,8,18–21]. Using light to dynamically control the shape and function of SCCs is a very promising strategy as it is a noninvasive stimulus that can be easily applied with high spatiotemporal control, without producing any waste. Systems have been reported where photoisomerization of azobenzene-derived anions encapsulated in supramolecular palladium complexes caused immediate crystallization [22]. Moreover, azobenzenes have been used to functionalize both the interior [23] and exterior [24] of SCCs to photochemically control guest binding and release. Furthermore, incorporation of dithienylethene into the ligands connecting the metal centers has been used to control host–guest interactions [25–28], structural composition [29], and sol–gel transition [30]. However, dithienylethene undergoes a limited structural change upon photoisomerization and, up to now, photoswitchable ligands based on other types of photochromic switches have not been reported in the literature.

In chiral self-sorting SCCs, either homo- or heterochiral complexes are formed exclusively through high fidelity recognition of the components within the complex [31–33]. The formation of such complexes with high selectivity and well-defined chirality is essential for the application of SCCs towards chiral recognition and sensing [34–37]. To the best of our knowledge no self-sorted responsive SCCs have been reported so far.

Molecular motors based on overcrowded alkenes are unique photoswitches that are able to undergo unidirectional rotation upon irradiation with light [38–40]. Moreover, they can be used as chiroptical multistate switches to control various functions in areas such as catalysis [41–43], soft materials [43–45] and supramolecular chemistry [46–47]. Employing molecular motors as ligands in SCCs provides an interesting strategy to form responsive coordination complexes, as they feature a large geometric change upon switching [47].

**Figure 1:**
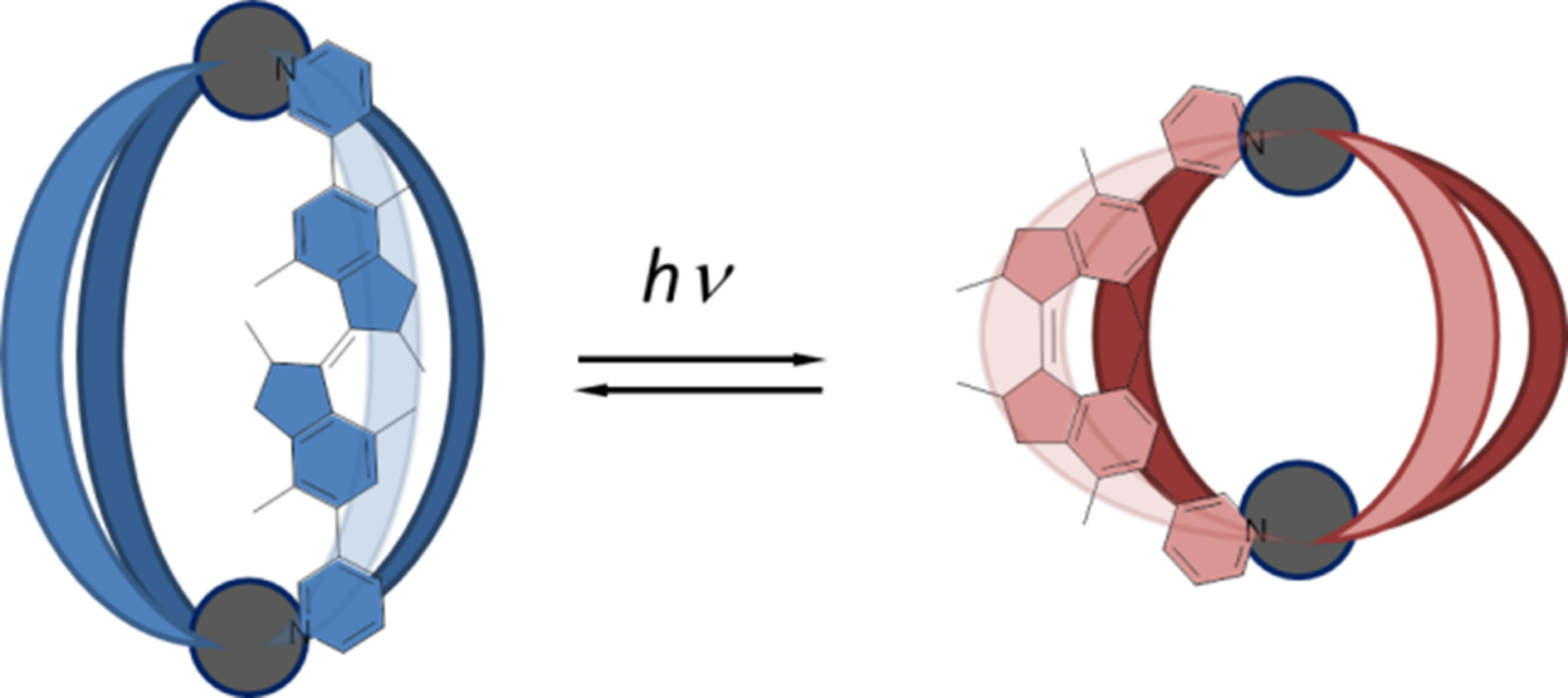
Schematic representation of a photoresponsive cage with ligands based on overcrowded alkenes.

**Scheme 1:**
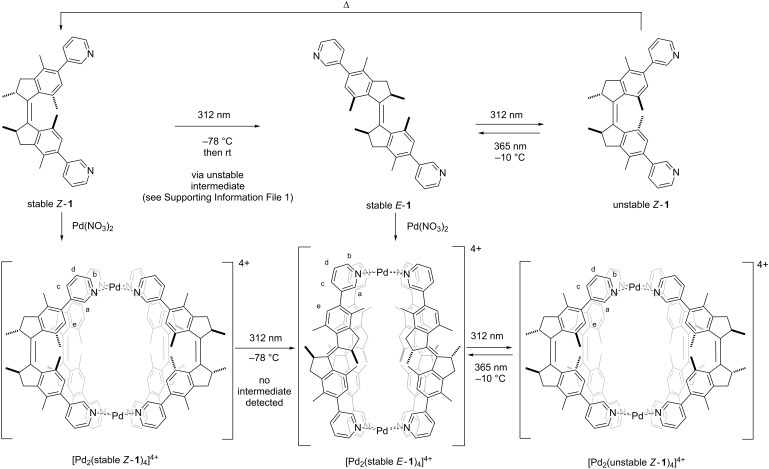
Cage formation of overcrowded alkene switches *E*/*Z*-**1** and their isomerization behavior. Note that the intermediate unstable *E*-**1** is not shown; due to the low barrier of thermal helix inversion and therefore fast isomerization to the corresponding stable isomer.

Herein, we report a photoresponsive coordination cage with ligands based on a first generation molecular motor (Figure 1). Cages with a Pd_2_L_4_ composition are formed from bidentate bispyridyl ligands and Pd(II) ions with a square planar geometry, which have been widely studied [6,48–50]. The photochromic ligands can be switched between three states, forming separate discrete cage complexes, allowing cage-to-cage transformations (Scheme 1). Interestingly, only homochiral cages are formed revealing that a chiral self-sorting process takes place. In addition, two of the cage isomers can bind a tosylate anion in solution by formation of a host–guest complex.

## Results and Discussion

**Figure 2:**
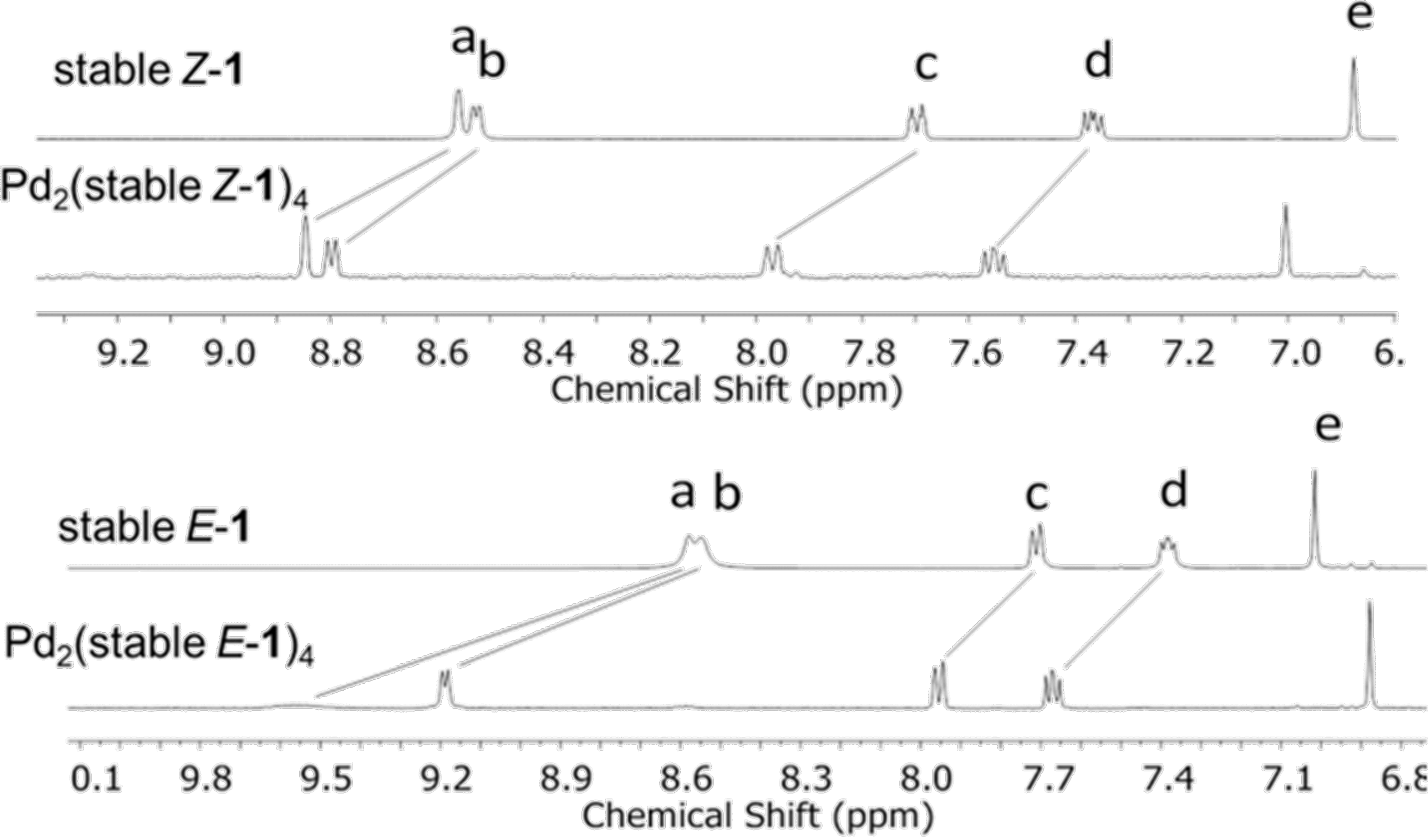
Aromatic region of stacked ^1^H NMR spectra (in CD_3_CN) of stable *Z*-**1** and cage complex Pd_2_(stable *Z*-**1**)_4_ (top) and *E*-**1** and cage complex Pd_2_(stable *E*-**1**)_4_ (bottom).

Ligands *Z-***1** and *E-***1** (Scheme 1) were synthesized by a Suzuki cross-coupling reaction of 3-pyridinylboronic acid with an *E*/*Z* mixture of reported overcrowded alkene precursors [51] (see Supporting Information File 1 for full experimental details). Heating a 2:1 mixture of ligands (*S,S*)-*Z-***1** or (*S,S*)-*E*-**1** with Pd(NO_3_)_2_ in acetonitrile at reflux led to the quantitative formation of cage Pd_2_(stable *Z*-**1**)_4_ or Pd_2_(stable *E*-**1**)_4_, respectively, as evidenced by ^1^H NMR, DOSY and HRMS. The ^1^H NMR signals of the pyridine moieties of the ligands (H_a–d_) in the assembled cages are shifted downfield compared to those of the free ligands, as expected due to metal coordination (Figure 2) [28]. As the ligand exchange in Pd_2_L_4_ complexes is slow on the NMR timescale, the discrete signals do not represent an average of quickly interconverting isomers [52–53]. The formation of cage complexes using a racemic mixture of ligands stable *Z*-**1** or stable *E*-**1**, resulted in the exact same ^1^H NMR spectrum as was obtained with the enantiopure ligands. Using a racemic mixture of ligands, four different diastereomeric cages can be formed ((*S*,*S*)_4_, (*S*,*S*)_3_(*R,R*), (*S*,*S*)_2_(*R*,*R*)_2_ and (*S*,*S*)(*R*,*R*)(*S,S*)(*R,R*) and their enantiomeric pairs). However, in both cases, only one set of signals is observed, which is a strong indication that only one species with high symmetry is formed by chiral self-sorting without any sign of the formation of diastereomeric mixtures. To further confirm the self-sorting behavior of these cage structures, CD spectroscopy was performed. A linear dependence of CD amplitude on the ee of ligand stable *Z*-**1** was found, which can be expected when the homochiral enantiomers of cage structure Pd_2_(stable *Z*-**1**)_4_ are the only optically active species in solution (Figure S1, Supporting Information File 1).

**Figure 3:**
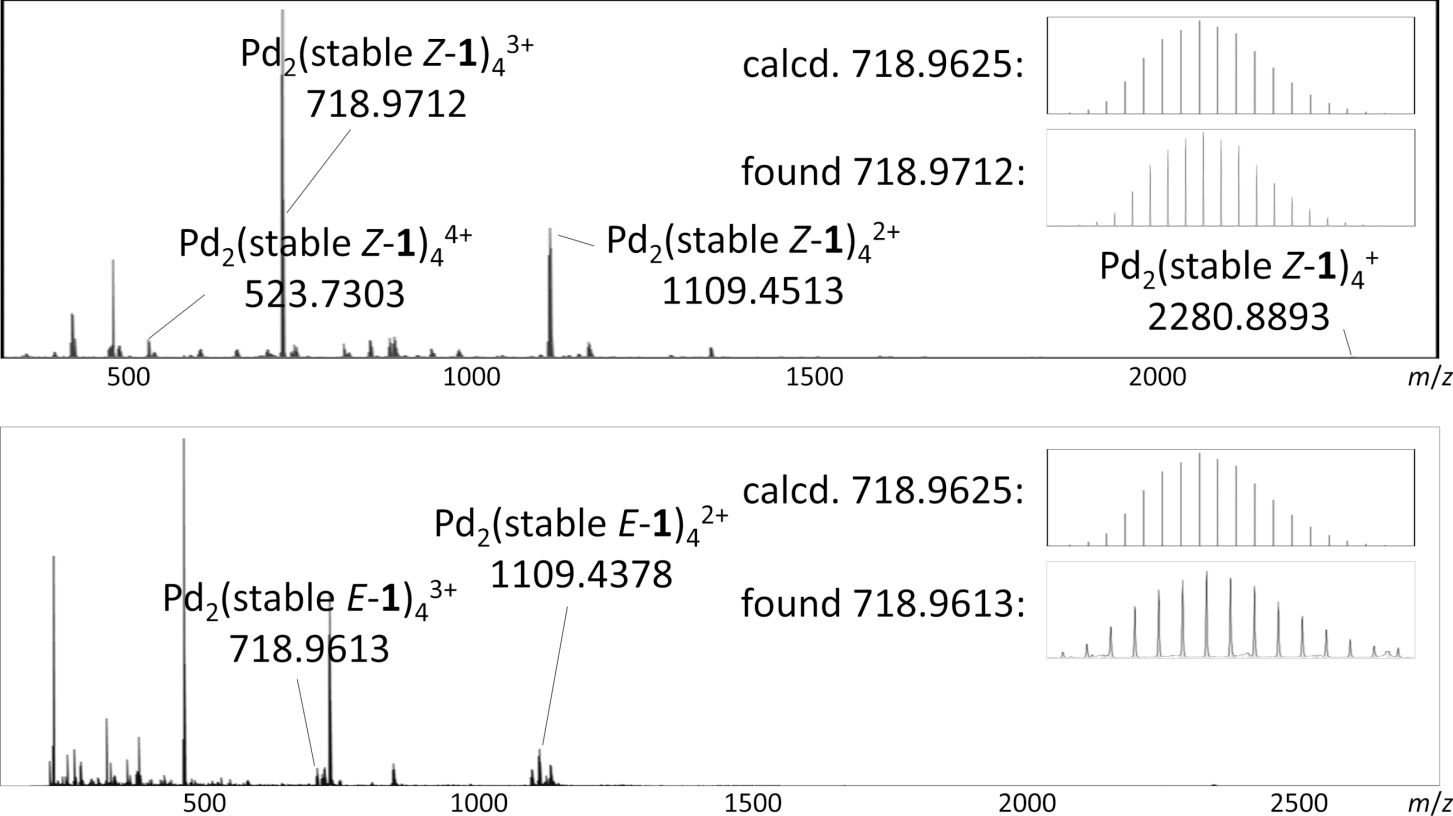
HRMS spectra of cage complex Pd_2_(stable *Z*-**1**)_4_ (top) and cage complex Pd_2_(stable *E*-**1**)_4_ (bottom); Insets: comparison of simulated and measured isotopic patterns of Pd_2_L_4_(NO_3_)^3+^ ions.

Additionally, DOSY NMR spectroscopy revealed that the signals correspond to a single type of assembly in each case (see Supporting Information File 1, section 2). The measured diffusion coefficients (*D =* 8.7 × 10^−10^ m^2^ s^−1^ for Pd_2_(stable *Z*-**1**)_4_ and *D* = 7.9 × 10^−10^ m^2^ s^−1^ for cage Pd_2_(stable *E*-**1**)_4_ in CD_3_CN at 23 °C) can be translated into hydrodynamic radii of *r*_H_ = 7.2 Å for Pd_2_(stable *Z*-**1**)_4_ and *r*_H_ = 8.0 Å for Pd_2_(stable *E*-**1**)_4_ by using the Stokes–Einstein equation [54]. By means of ESI high-resolution mass spectrometry, we were able to verify the Pd_2_L_4_ constitution of both cages. The HRMS spectrum of Pd_2_(stable *Z*-**1**)_4_ shows the signals for the cations [Pd_2_(stable *Z*-**1**)_4_(NO_3_)_3_]^+^, [Pd_2_(stable *Z*-**1**)_4_(NO_3_)_2_]^2+^, [Pd_2_(stable *Z*-**1**)_4_(NO_3_)]^3+^, [Pd_2_(stable *Z*-**1**)_4_]^4+^ (Figure 3). For Pd_2_(stable *E*-**1**)_4_, the peaks corresponding to the cations [Pd_2_(stable *E*-**1**)_4_(NO_3_)_2_]^2+^ and [Pd_2_(stable *E*-**1**)_4_(NO_3_)]^3+^ were observed (Figure 3). For both isomers, the experimental isotopic patterns and exact *m*/*z* values match the simulated patterns.

A single crystal of Pd_2_(stable *E*-**1**)_4_ formed from a racemic mixture of ligand *E*-**1** suitable for X-ray structure determination was grown by vapor diffusion of a 1:1 mixture of benzene and diethyl ether into a solution of the cage in a 1:1 mixture of acetonitrile and chloroform. The crystal structure shows cage structures with a Pd_2_L_4_ stoichiometry with one NO_3_^−^ counterion and one molecule of acetonitrile located inside each cage. In addition, a chloride ion is located close to the metal centers outside of the cage. This counterion most likely originates from the solvent, as chloroform can contain considerable amounts of HCl. The additional anions required to balance the charge of the tetracationic Pd_2_L_4_ cage could not be unambiguously located in the difference Fourier map (see Supporting Information File 1 for details). The structure belongs to the *P*4/*n* space group and the unit cell is occupied by a pair of enantiomeric cages in which the Pd–Pd axis of each cage is located at the 4-fold rotation axis. This means that the cage structure is made up with exclusively (*R,R*) or (*S,S*) enantiomer of the ligand and that the elementary cell is built from the racemic pair of cages.

**Figure 4:**
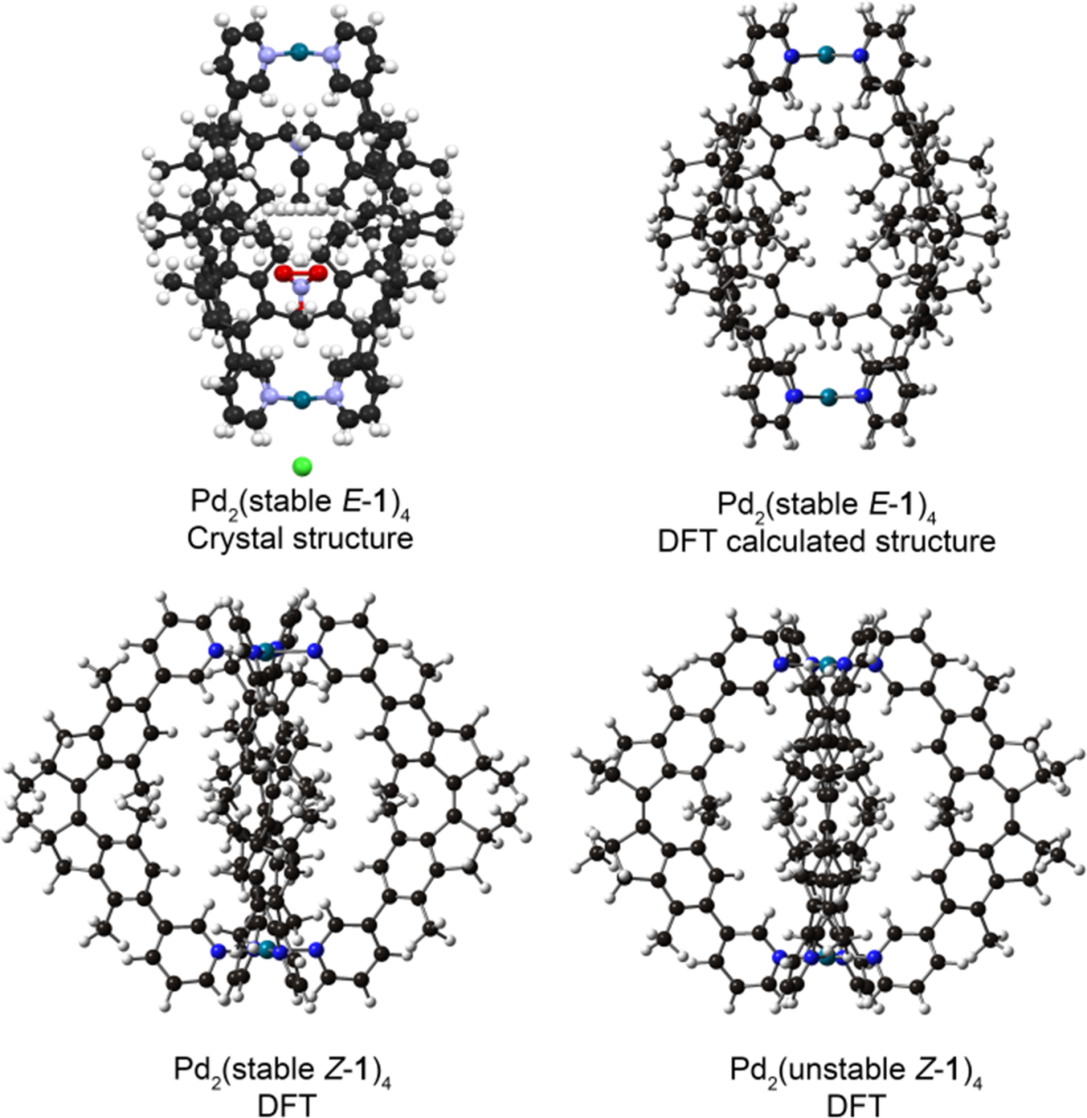
Crystal structure of cage complex Pd_2_(stable *E*-**1**)_4_ (top left) and DFT optimized structures of cage complexes Pd_2_(stable *E*-**1**)_4_, Pd_2_(stable *Z*-**1**)_4_ and Pd_2_(unstable *Z*-**1**)_4_. For clarity only one of the enantiomeric cages was depicted on the figure (carbon – black, nitrogen – blue, oxygen – red, palladium – cyan, chlorine – green, hydrogen – white).

DFT calculations were performed to gain insight in the self-sorting behavior of cages Pd_2_(stable *Z*-**1**)_4_ and Pd_2_(stable *E*-**1**)_4_. The structures of all possible cage diastereomers were optimized using B3LYP/6-31G(d) for C,H,N and LANL2DZ with ECP for Pd in the gas phase without counter ions. The optimized structure of Pd_2_(stable *E*-**1**)_4_ is in good agreement with the solved X-ray structure (Figure 4). Moreover, the calculations revealed that the homochiral cage Pd_2_((*S,S*)-stable *E*-**1**)_4_ (and its enantiomer) are energetically favored by at least 61 kJ mol^−1^ compared to the other possible diastereomers (Table S1, Supporting Information File 1). Similar calculations on the diastereomers of Pd_2_(stable *Z*-**1**)_4_ revealed that the homochiral cage diastereomers Pd_2_((*S,S*)-stable *Z*-**1**)_4_ are energetically favored as well, by at least 19 kJ mol^−1^ (Table S2, Supporting Information File 1). These calculations support that these cages are formed by chiral narcissistic self-sorting.

Next, we were interested in the guest binding abilities of cages Pd_2_(stable *Z*-**1**)_4_ and Pd_2_(stable *E*-**1**)_4_. The tosylate anion was chosen as it has the appropriate size to fit inside the cages. A Job plot analysis revealed a 1:1 binding stoichiometry between both cage isomers and OTs^−^ (Figures S3–S5, Supporting Information File 1), which corresponds to the model in which OTs^−^ serves as a guest molecule which is encapsulated inside the cages [55–56]. ^1^H NMR titrations with tetrabutylammonium tosylate revealed that both cages are able to bind OTs^−^, showing similar binding strengths (*K*_B_ = 1604 ± 39 M^−1^ for Pd_2_(stable *Z*-1)_4_; *K*_B_ = 1758 ± 39 M^−1^ for Pd_2_(stable *E*-**1**)_4_ at 293 K).

Finally, the photochemical and thermal isomerization behavior of the complexes was studied. Initially, the behavior of ligand **1** was studied by UV–vis and NMR spectroscopy, showing similar behavior as related first generation molecular motors (see Supporting Information File 1 for full details). Irradiation with 312 nm light at −70 °C isomerizes stable *Z*-**1** to unstable *E*-**1**, which undergoes a thermal helix inversion (THI) when warmed to room temperature to form stable *E*-**1**. The second half of the rotation cycle is similar, as irradiation with 312 nm light isomerizes stable *E*-**1** to unstable *Z*-**1**, which undergoes THI to form stable *Z*-**1** when left for several days at room temperature.

**Figure 5:**
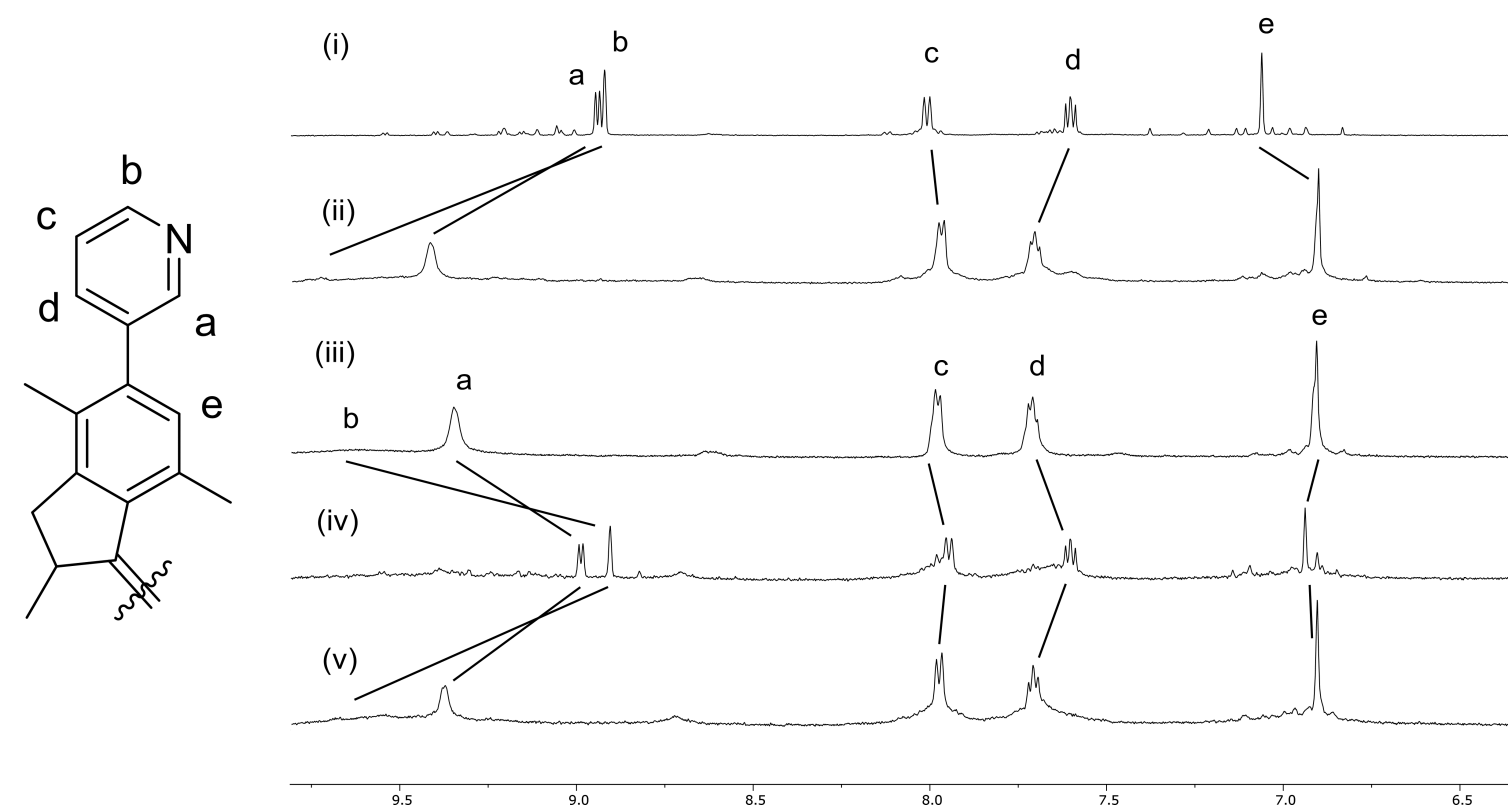
Aromatic region of stacked ^1^H NMR spectra (CD_3_CN/CD_2_Cl_2_ 1:1) of i) Pd_2_(stable *Z*-**1**)_4_; ii) Pd_2_(stable *E*-**1**)_4_ generated by irradiation of Pd_2_(stable *Z*-**1**)_4_ at 312 nm; iii) Pd_2_(stable *E*-**1**)_4_ prepared directly from ligand stable *E*-**1**; iv) Pd_2_(unstable *Z*-**1**)_4_ generated by irradiation of Pd_2_(stable *E*-**1**)_4_ at 312 nm and v) Pd_2_(stable *E*-**1**)_4_ generated by irradiation of Pd_2_(unstable *Z*-**1**)_4_ at 365 nm.

Subsequently, the photochemical and thermal isomerizations of cages Pd_2_(stable *Z*-**1**)_4_ and Pd_2_(stable *E*-**1**)_4_ were followed by ^1^H NMR studies (Figure 5). Irradiation of Pd_2_(stable *Z*-**1**)_4_ in a CD_3_CN/CD_2_Cl_2_ 1:1 mixture at 312 nm at −70 °C was performed to isomerize ligand stable *Z*-**1** to unstable *E*-**1** (vide infra), followed by allowing the sample to warm to room temperature to form stable *E*-**1** (Figure 5ii). The ^1^H NMR spectrum of this newly formed complex is identical to the spectrum of Pd_2_(stable *E*-**1**)_4_ prepared directly from *E*-**1** (Figure 5iii), showing that cage Pd_2_(stable *Z*-**1**)_4_ is effectively converted to Pd_2_(stable *E*-**1**)_4_. An intermediate complex containing unstable *E*-**1** ligands was not observed, even at low temperatures, most likely due to the low barrier for THI of this isomer. Conversion of cage Pd_2_(stable *E*-**1**)_4_ to Pd_2_(unstable *Z*-**1**)_4_ by photochemical *E*/*Z* isomerization of ligand stable *E*-**1** to unstable *Z*-**1** was performed by irradiation of a sample of Pd_2_(stable *E*-**1**)_4_ at 312 nm at −20 °C (Figure 5iv). Signals of cage Pd_2_(stable *Z*-**1**)_4_ disappeared and the formation of a new set of signals was observed. DOSY NMR confirmed the formation of an assembly with a hydrodynamic radius which was similar to that of the cage Pd_2_(stable *Z*-**1**)_4_. Precipitation of the metal centers in this assembly using tetrabutylammonium glutarate liberates the ligands and they were identified as unstable *Z*-**1**. Combined, these results confirm that the photogenerated complex is indeed Pd_2_(unstable *Z*-**1**)_4_. Subsequent irradiation of this sample containing Pd_2_(unstable *Z*-**1**)_4_ at −20 °C at 365 nm converts the unstable *Z*-**1** ligands back to stable *E*-**1**, reforming Pd_2_(stable *E*-**1**)_4_ (Figure 5v). These experiments highlight the reversible formation of Pd_2_(unstable *Z*-**1**)_4_ through photochemical *E*/*Z* isomerization of the ligands.

On the other hand, allowing the THI of ligands unstable *Z*-**1** in cage Pd_2_(unstable *Z*-**1**)_4_ to take place by leaving the solution at room temperature for 5 d did not lead to the formation of Pd_2_(stable *Z*-**1**)_4_, but to disassembly of the cage and formation of ill-defined complexes. Precipitation of the metal centers in these complexes identified the ligands as a mixture of both stable *Z*-**1** and stable *E*-**1** (originating from the PSS mixture), indicating that the THI does take place. A possible explanation could be that the mixture of stable *Z*-**1** and stable *E*-**1** does not form separate well-defined cage structures, but forms mixed complexes.

## Conclusion

In summary, a new photoresponsive supramolecular coordination complex based on overcrowded alkenes is presented, allowing switching between three different cage structures. Interestingly, the cage structures with Pd_2_L_4_ constitution were shown to be homochiral, forming single diastereomers as evident from NMR, CD and X-ray analysis, supported by DFT calculations. Additionally, the cage structures were able to bind OTs^−^ inside their cavity. Although photoswitching affords a large geometric change of the ligands, only minor changes were observed in binding constants of the different cage structures. These results show that by incorporation of overcrowded alkenes into SCCs the geometry of cage structures can be controlled by light. Different designs might be considered to translate these geometrical changes to changes in properties such as guest binding affinity and selectivity.

## Supporting Information

File 1 Experimental procedures, compound characterization, CD spectroscopy, binding studies, NMR studies of the photochemical and thermal isomerizations, X-ray crystallography, computational details and Cartesian coordinates of DFT optimized structures.
