# Supplementary material for: A chiral self-sorting photoresponsive coordination cage based on overcrowded alkenes
Source: Beilstein J Org Chem. 2019 Nov 15;15:2767–73. doi: 10.3762/bjoc.15.268 (PMC6880828; doi:10.3762/bjoc.15.268)
Supplement: File 1 — Experimental procedures, compound characterization, CD spectroscopy, binding studies, NMR studies of the photochemical and thermal isomerizations, X-ray crystallography, computational details and Cartesian coordinates of DFT optimized structures. [file Beilstein_J_Org_Chem-15-2767-s001.pdf]

BEILSTEIN JOURNAL OF ORGANIC CHEMISTRY

Supporting Information

for

A chiral self-sorting photoresponsive coordination cage based on overcrowded alkenes

Constantin Stuckhardt, Diederik Roke, Wojciech Danowski, Edwin Otten,
Sander J. Wezenberg and Ben L. Feringa

Beilstein J. Org. Chem. **2019,** *15,* 2767–2773. doi:10.3762/bjoc.15.268

Experimental procedures, compound characterization, CD spectroscopy, binding studies, NMR studies of the photochemical and thermal isomerizations, X-ray crystallography, computational details and Cartesian coordinates of DFT optimized structures

License and Terms: This is a supporting information file under the terms of the Creative Commons Attribution License (http://creativecommons.org/licenses/by/4.0). Please note that the reuse, redistribution and reproduction in particular requires that the authors and source are credited.
The license is subject to the *Beilstein Journal of Organic Chemistry* terms and conditions: (https://www.beilstein-journals.org/bjoc)

Table of Contents

I.	General procedures …………………………………………………………	S2
II.	Experimental procedures and compound characterization …	S3
III.	CD spectroscopy ………………………………………………………………	S9
IV.	Binding studies ………………………………………………………………..	S10
V.	Photochemical and thermal isomerization studies ……………	S13
VI.	X-ray crystallography ………………………………………………………..	S17
VII.	Computational details ………………………………………………………	S18
VIII.	Carthesian coordinates of DFT optimized structures ………..	S23

I. General procedures

All reactions involving air-sensitive reagents were performed under a dry N_2 atmosphere. Anhydrous THF was used from a solvent purification system (MBRAUN SPS systems, MBSPS-800). All other chemicals were commercial products and were used as received. Flash column chromatography was performed using silica gel (SiO_2) purchased from Merck (type 9385, 230–400 mesh). Thin-layer chromatography (TLC) was carried out on aluminum sheets coated with silica 60 F253 obtained from Merck. Compounds were visualized with a UV lamp (254 nm) or by staining with CAM. Melting points (mp) were determined using a Büchi-B545 capillary melting point apparatus. ^1H and ^{13}C NMR spectra were recorded on a Varian Mercury-Plus 400 or a Varian Unity-Plus 500 spectrometer at 298 K unless indicated otherwise. Chemical shifts are quoted in parts per million (ppm) relative to either $CDCl_3$ (δ 7.26 for ^1H, δ 77.16 for ^{13}C) or CD_2Cl_2 (δ 5.32 for ^1H, δ 53.84 for ^{13}C) or CD_3CN (δ 1.94 for ^1H, also used as reference for solvent mixtures containing CD_3CN). For ^1H NMR spectroscopy, the splitting pattern of peaks is designated as follows: s (singlet), d (doublet), t (triplet), m (multiplet), p (quintett), or dd (doublet of doublets). High resolution mass spectrometry (ESIMS) was performed on a LTQ Orbitrap XL spectrometer for organic compounds and on a Bruker Maxis Plus QTOF spectrometer for coordination compounds with ESI ionisation. UV–vis and CD spectra were recorded in a 1 cm quartz cuvette. CD spectra were recorded on a Jasco 810 CD spectropolarimeter. UV–vis absorption spectra were recorded on an Agilent 8453 UV–vis Spectroscopy System. The UV–is and NMR irradiation experiments with λ_{max} = 312 nm were carried out with a Spectroline ENB-280C/FE lamp (8 W) while cooling the irradiated sample in an ethanol bath. Solvents used for irradiation experiments were degassed by means of at least three freeze-pump-thaw cycles and CD_2Cl_2 as well as CH_2Cl_2 were filtered over K_2CO_3 prior to use. The exact temperature during NMR measurements was determined by measuring the electric resistance with a Keithley 197 Autoranging Microcolt Digital Multimeter which was then translated into the temperature by means of a Pt100 table.

II. Experimental procedures and compound characterization

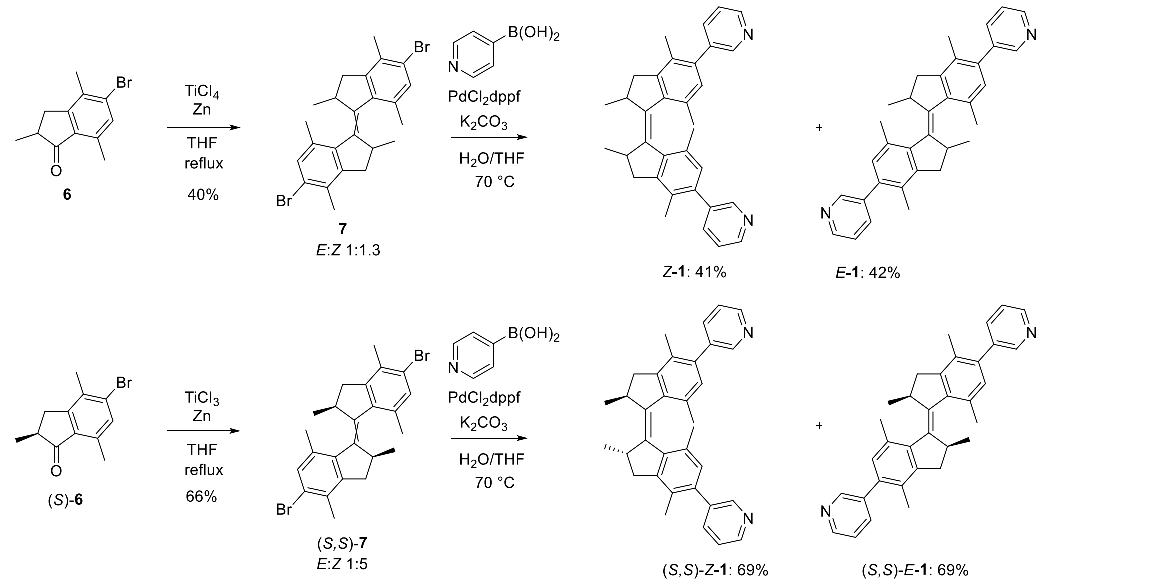

Scheme S1: Synthesis of overcrowded alkene-based ligands **1**

Racemic and enantiopure ketone **6** and motor **7** were synthesized according to literature procedures.[1]

(*Z*)-3,3'-(2,2',4,4',7,7'-hexamethyl-2,2',3,3'-tetrahydro-[1,1'-biindenylidene]-5,5'-diyl)dipyridine (*Z*-1) and (*E*)-3,3'-(2,2',4,4',7,7'-hexamethyl-2,2',3,3'-tetrahydro-[1,1'-biindenylidene]-5,5'-diyl)dipyridine (*E*-1)

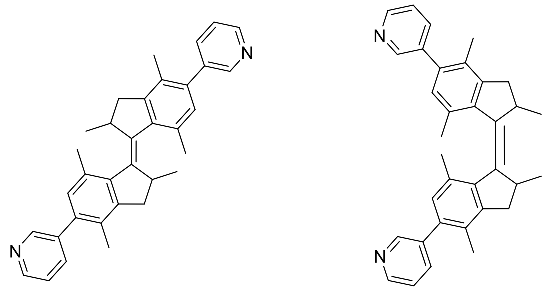

E-**1** *Z*-**1**

A 1.5:1 *E/Z* mixture of motor **7** (700 mg, 1.48 mmol, 1.0 equiv), 3-pyridinylboronic acid (454 mg, 3.69 mmol, 2.5 equiv), [1,1′-bis(diphenylphosphino)ferrocene]dichloropalladium(II), complex with CH_2Cl_2 (60 mg, 73.8 µmol, 0.05 equiv) and K_2CO_3 (1.40 g, 10.1 mmol, 6.8 equiv) were dissolved in a mixture of water (7.4 mL) and THF (21 mL). The mixture was degassed by purging with N_2 for 30 min and then stirred at 70 °C for 2 d. Then, the mixture was diluted with CH_2Cl_2 (50 mL) and washed with brine (50 mL). The organic phases were combined and dried over Mg_2SO_4, volatiles were removed in vacuo and the residue was purified

[1] T. M. Neubauer, T. van Leeuwen, D. Zhao, A. S. Lubbe, J. C. M. Kistemaker, B. L. Feringa, *Org. Lett.* **2014**, *16*, 4220–4223.

using column chromatography (SiO_2, CH_2Cl_2 + 2.5% MeOH) to give ligands *Z*-**1** (343 mg, 0.73 mmol, 82%) and *E*-**1** (233 mg, 0.49 mmol, 83%) as off-white solids.

Z-**1**: ^{1}H-NMR (400 MHz, $CDCl_3$) δ (ppm) = 8.64 (s, 2H), 8.58 (s, 2H), 7.72 (d, J = 7.8 Hz, 2H), 7.37 (s, 2H), 6.86 (s, 2H), 3.43 (t, J = 6.6 Hz, 2H), 3.18 (dd, J = 14.9, 6.4 Hz, 2H), 2.53 (d, J = 15.4 Hz, 2H), 2.20 (s, 6H), 1.61 (s, 6H), 1.15 (d, J = 6.8 Hz, 6H).

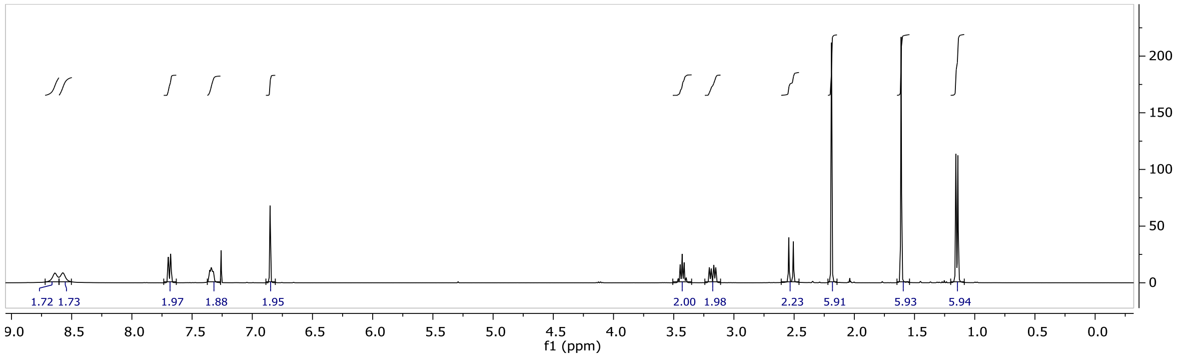

^{13}C-NMR (151 MHz, $CDCl_3$) δ (ppm) = 150.23, 147.72, 145.64, 141.04, 140.91, 137.97, 136.87, 136.84, 133.35, 129.99, 128.27, 123.05, 41.89, 39.55, 20.81, 20.70, 16.42.

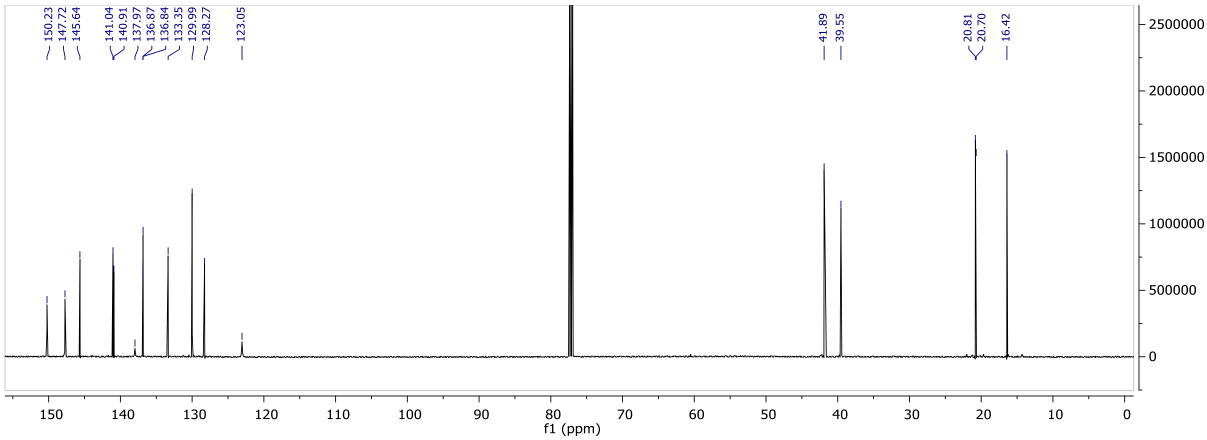

Mp 269 °C

HRMS (ESI+): calcd for $C_{34}H_{35}N_2^+$ (M+H$^+$): 471.2795, found 471.2763.

The enantiopure ligand (*S,S*)-*Z*-**1** was prepared according to the same procedure employing a mixture of enantiopure precursors (*S,S*)-*Z*-**7** and (*S,S*)-*E*-**7** with >99% ee as determined by chiral HPLC analysis, Chiralpak AD-H (90% heptane/10% iPrOH), 0.5 mL/min, retention times (min) 12.4 (major) and 15.5 (minor).

E-**1**: ^{1}H-NMR (400 MHz, $CDCl_3$) δ (ppm) = 8.66 (s, 2H), 8.59 (s, 2H), 7.73 (d, *J* = 7.7 Hz, 2H), 7.38 (s, 2H), 7.00 (s, 2H), 3.22 – 2.89 (m, 2H), 2.79 (dd, *J* = 14.7, 5.7 Hz, 2H), 2.50 (s, 6H), 2.34 (d, *J* = 14.6 Hz, 2H), 2.13 (s, 6H), 1.17 (d, *J* = 6.5 Hz, 6H).

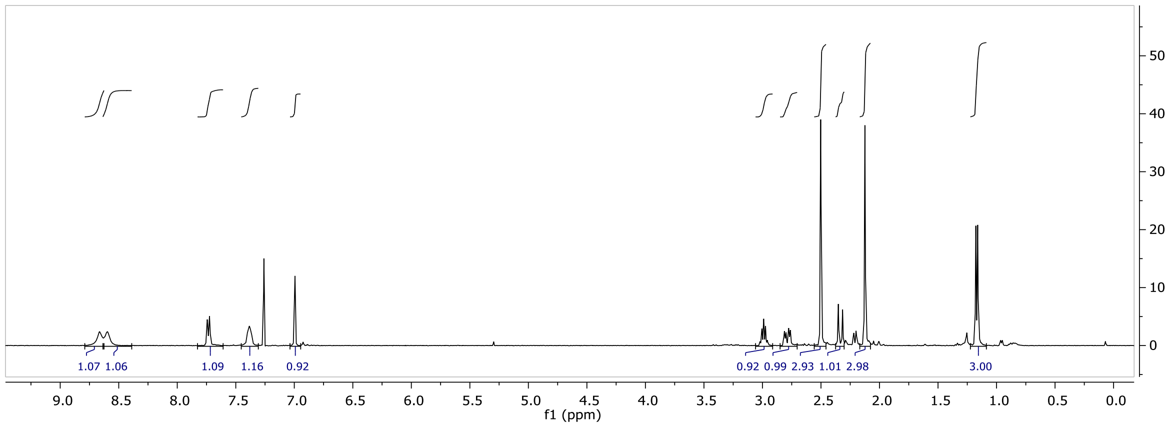

^{13}C-NMR (101 MHz, $CDCl_3$) δ (ppm) = 150.30, 147.90, 143.97, 141.70, 140.98, 137.82, 136.89, 136.81, 131.27, 130.48, 129.05, 123.03, 42.21, 39.76, 22.01, 19.69, 16.30.

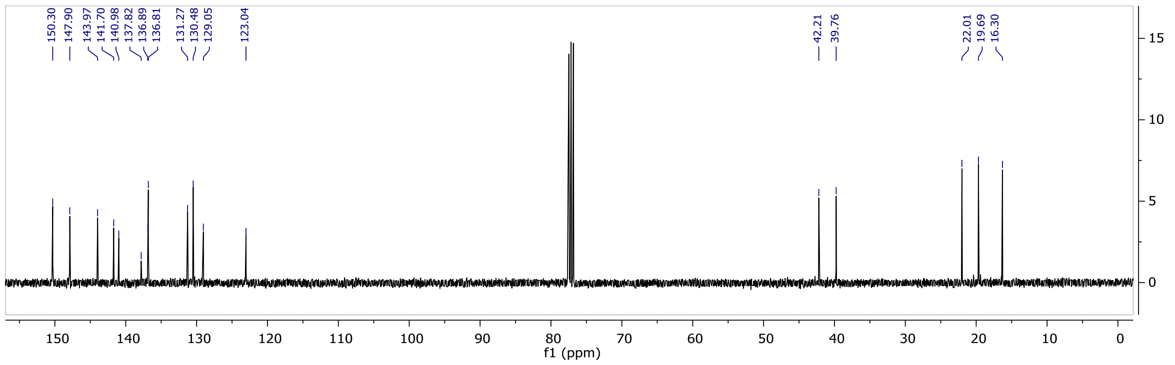

Mp 235-237 °C

HRMS (ESI+): calcd for $C_{34}H_{35}N_2^+$ (M+H$^+$): 471.2795, found 471.2764.

The enantiopure ligand (*S*,*S*)-*E*-**1** was prepared according to the same procedure employing a mixture of enantiopure precursors (*S*,*S*)-*Z*-**7** and (*S*,*S*)-*E*-**7** with >99% ee as determined by chiral HPLC analysis, Chiralpak AD-H (90% heptane/10% iPrOH), 0.5 mL/min, retention times (min) 11.3 (major) and 12.6 (minor).

Cage formation

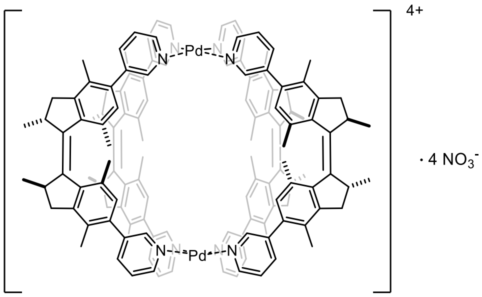

Pd$_2$(stable *Z*-**1**)$_4$

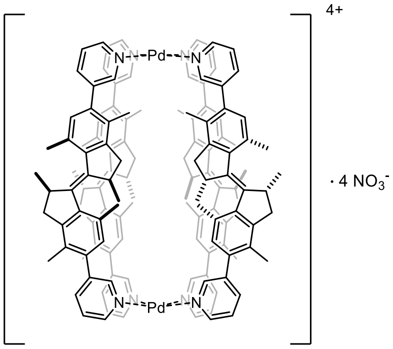

Pd$_2$(stable *E*-**1**)$_4$

A solution ($c \leq 2.5$ mM) of 1.0 equiv of $Pd(NO_3)_2$ in CD_3CN, alternatively in a mixture with CD_2Cl_2, was added to 2.0 equiv of either *Z*-**1** or *E*-**1** in a closed vial and the mixture was heated at reflux until a clear solution was obtained to yield either Pd$_2$(stable *Z*-**1**)$_4$ or Pd$_2$(stable *E*-**1**)$_4$ in solution.

Pd$_2$(stable *Z*-**1**)$_4$: ^{1}H NMR (500 MHz, CD_3CN) δ (ppm) = 8.81 (s, 8H), 8.67 (d, *J* = 5.8 Hz, 8H), 7.99 (d, *J* = 8.4 Hz, 8H), 7.55 (dd, *J* = 8.1, 5.7 Hz, 8H), 7.00 (s, 8H), 3.52 – 3.31 (m, 8H), 3.05 (dd, *J* = 14.8, 6.2 Hz, 8H), 2.48 (dd, *J* = 14.9, 7.7 Hz, 8H), 1.97 (s, 24H), 1.61 (s, 24H), 1.02 (d, *J* = 6.7 Hz, 24H). HRMS (ESI+): calcd for $C_{136}H_{136}N_{11}O_9Pd_2^{+}$ ([Pd$_2$(stable *Z*-**1**)$_4$(NO$_3$)$_3$]$^+$): 2280.8644, found 2280.8893; calcd for $C_{136}H_{136}N_{10}O_6Pd_2^{2+}$ ([Pd$_2$(stable *Z*-**1**)$_4$(NO$_3$)$_2$]$^{2+}$): 1109.4380, found 1109.4513; calcd for $C_{136}H_{136}N_9O_3Pd_2^{3+}$ ([Pd$_2$(stable *Z*-**1**)$_4$(NO$_3$)]$^{3+}$): 718.9625, found 718.9712; calcd for $C_{136}H_{136}N_8Pd_2^{4+}$ ([Pd$_2$(stable *Z*-**1**)$_4$]$^{4+}$): 523.7248, found 523.7303.

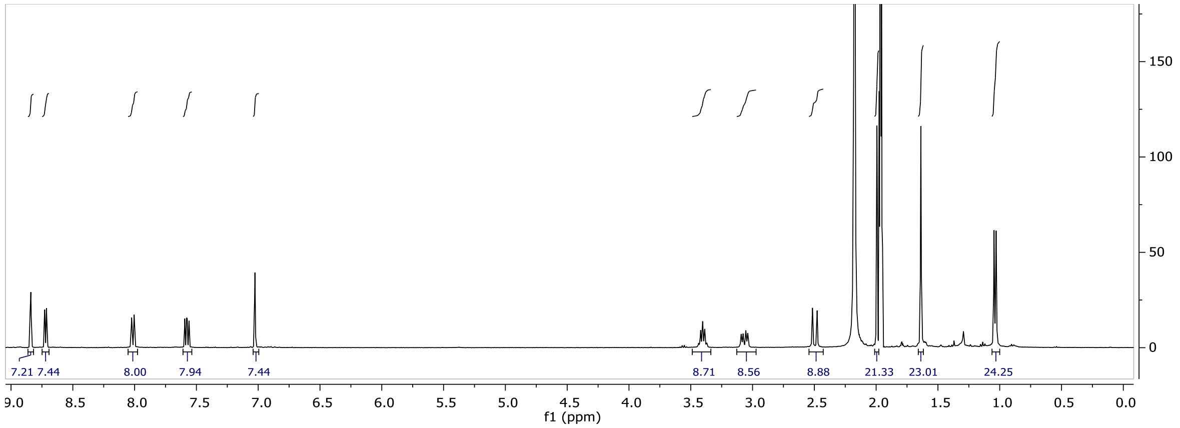

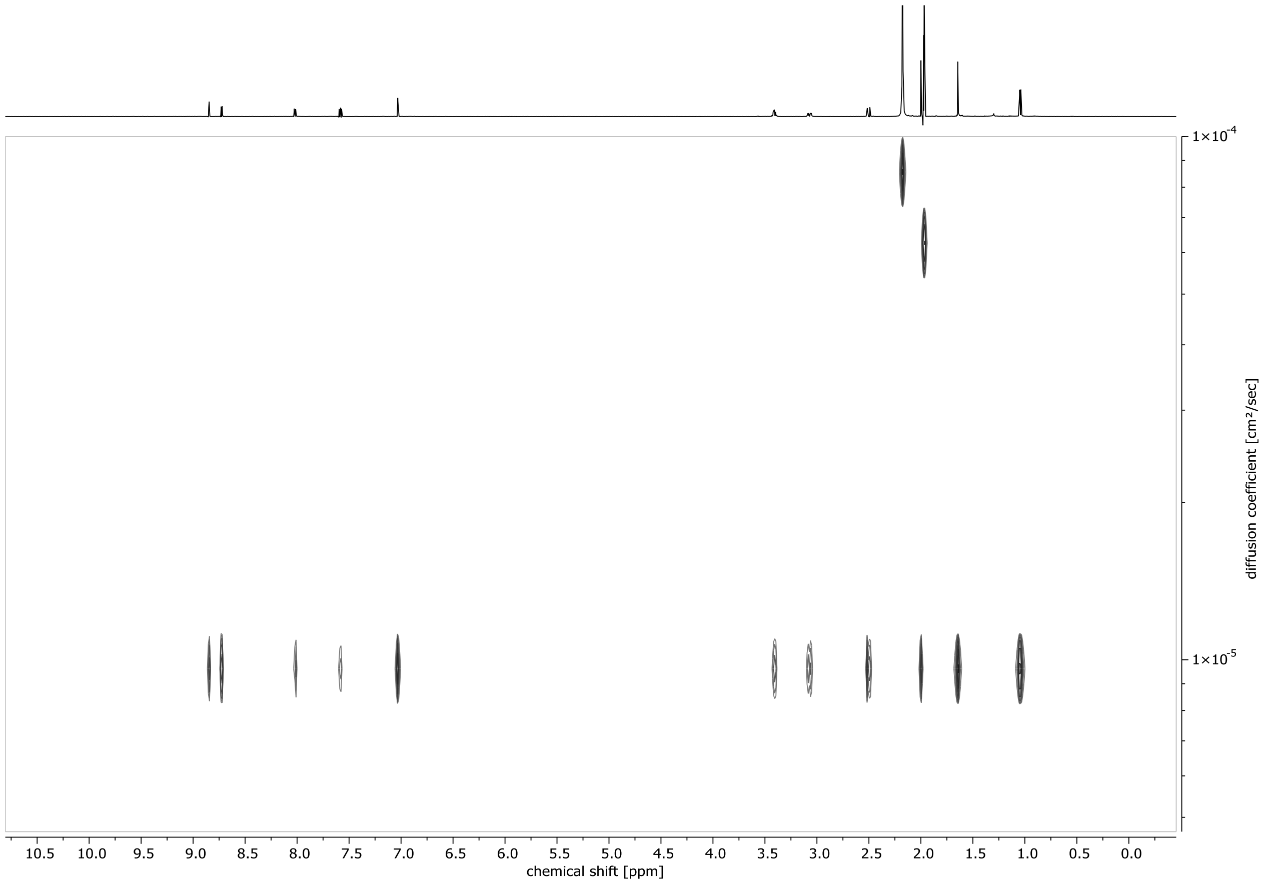

Pd_2(stable *E*-**1**)$_4$: ^{1}H NMR (500 MHz, CD_3CN) δ (ppm) = 9.56 (br, 8H), 9.16 (d, *J* = 5.7 Hz, 8H), 8.13 – 7.85 (m, 8H), 7.67 (dd, *J* = 7.7, 5.8 Hz, 8H), 6.88 (s, 8H), 2.93 (t, *J* = 6.3 Hz, 8H), 2.55 – 2.47 (m, 8H), 2.43 (s, 24H), 2.12 (d, *J* = 12.7 Hz, 8H, *covered by solvent signal), 1.52 (s, 24H), 1.08 (d, *J* = 6.4 Hz, 24H). HRMS (ESI+): calcd for $C_{136}H_{136}N_{10}O_6Pd_2^{2+}$ ([Pd_2(stable *E*-**1**)$_4$(NO_3)$_2$]$^{2+}$): 1109.4380, found 1109.4378; calcd for $C_{136}H_{136}N_9O_3Pd_2^{3+}$ ([Pd_2(stable *E*-**1**)$_4$(NO_3)]$^{3+}$): 718.9625, found 718.9613.

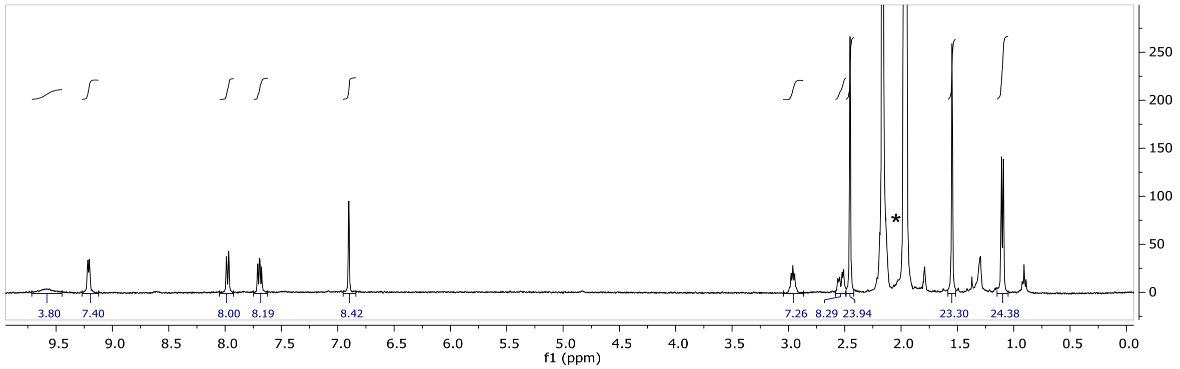

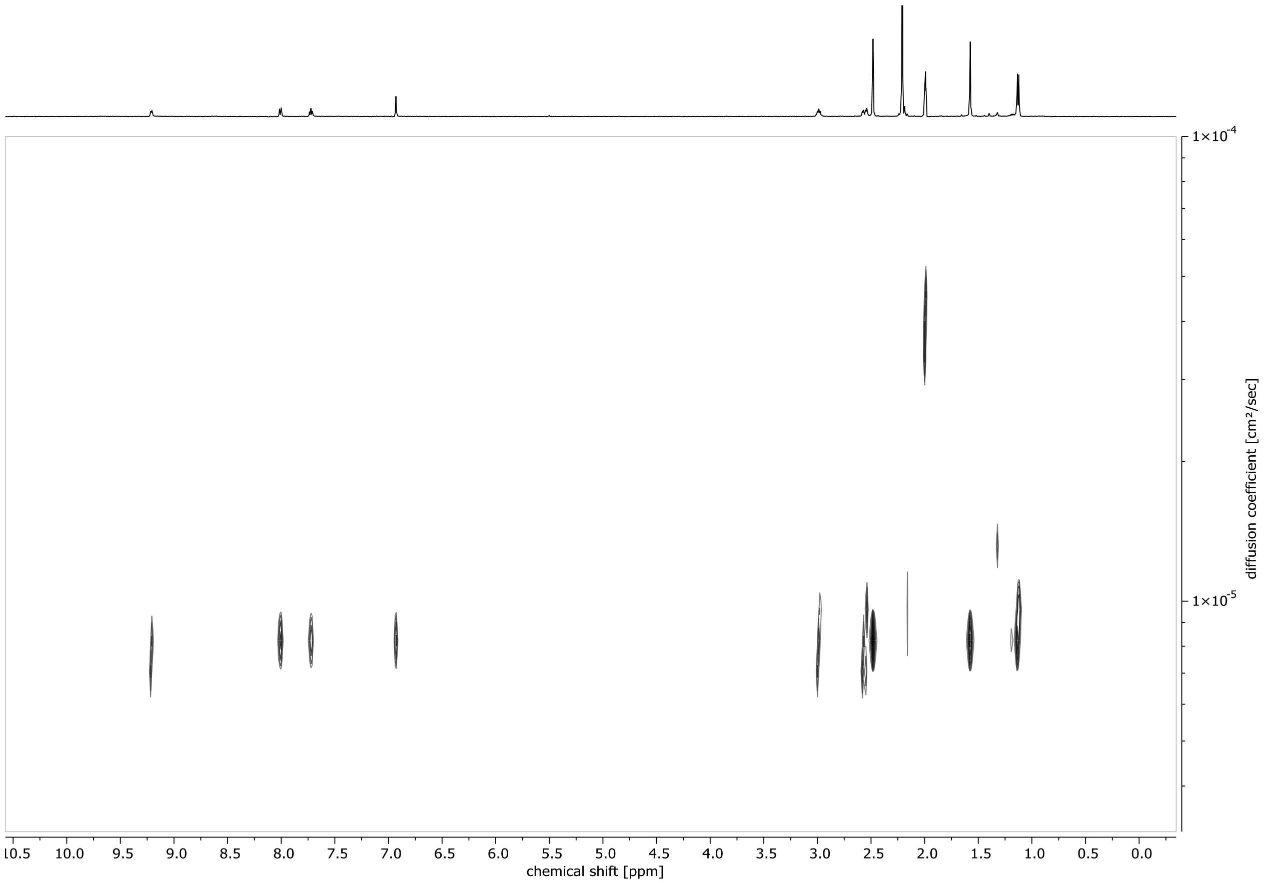

1×10⁻⁴
1×10⁻⁵
diffusion coefficient [cm²/sec]
10.5 10.0 9.5 9.0 8.5 8.0 7.5 7.0 6.5 6.0 5.5 5.0 4.5 4.0 3.5 3.0 2.5 2.0 1.5 1.0 0.5 0.0
chemical shift [ppm]

III. CD spectroscopy

Samples of cage Pd_2(stable *Z*-**1**)$_4$ in 0, 10, 20, 30, 40, 50, 60, 70, 80, 90 and 100% ee were prepared by mixing an enantiopure and a racemic stock solution (c = 1.1 · 10^{-5} M for both) and CD spectra were measured at 20 °C.

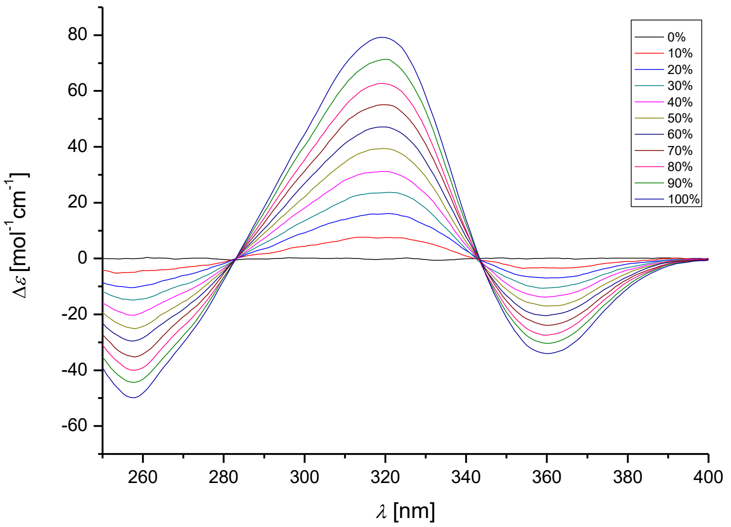

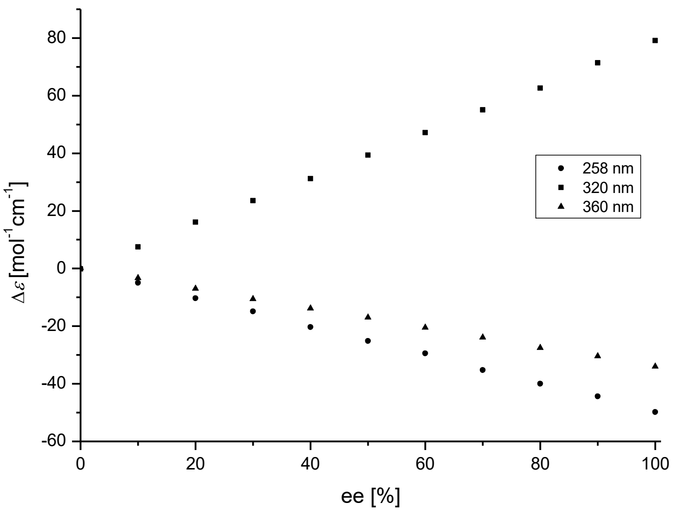

Figure S1: CD spectra of Pd_2(stable *Z*-**1**)$_4$ with varying ee (top). Plot of Δε versus the ee of Pd_2(stable *Z*-**1**)$_4$, showing a linear dependency (bottom).

IV. Binding studies

A Job plot analysis was performed by plotting the host-guest concentration ([HG]) versus the molar fraction of the host (χ). Different molar fractions were obtained by mixing stock solutions (c = $3.4 \cdot 10^{-4}$ M) of TBAOTs and Pd_2(stable *Z*-**1**)$_4$ or Pd_2(stable *E*-**1**)$_4$ in a 1:1 mixture of CD_3CN and CD_2Cl_2. The host guest concentration ([HG]) was then determined by equation 1, in which $[H]_0$ is the total concentration of host, δ_{obs} is the measured chemical shift of proton H_a in the host–guest mixture, δ_0 is the chemical shift of proton H_a for the pure host and $\delta_{complex}$ is the chemical shift of proton H_a in the host–guest complex which is assumed to be formed completely for a host–guest ratio of 1:9. Plotting [HG] *versus* the molar fraction of the host (*x*) yielded curves with maxima for *x* = 0.5, confirming a 1:1 binding stoichiometry (Figure S2).

$$[\mathrm{HG}] = [\mathrm{H}]_0 \cdot \frac{\delta_{\mathrm{obs}} - \delta_0}{\delta_{\mathrm{complex}} - \delta_0} \qquad (1)$$

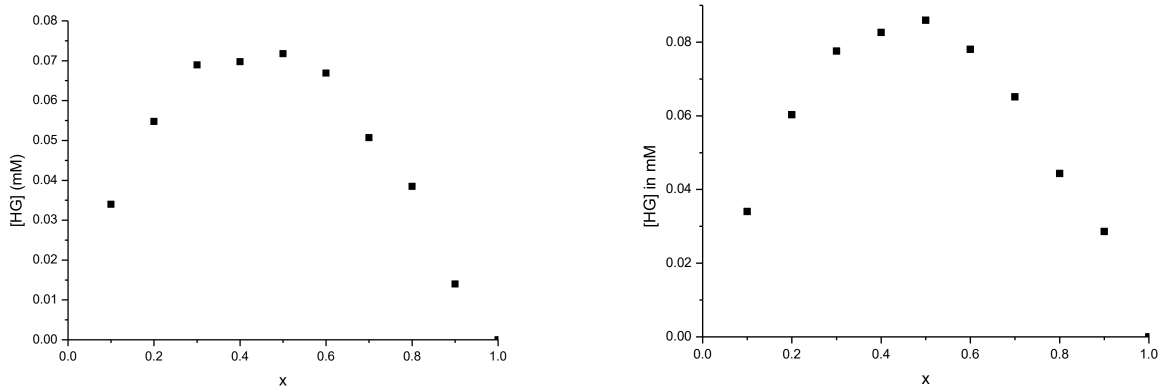

Figure S2: *Job plot of tosylate binding to Pd_2(stable Z-**1**)$_4$ (left) and Pd_2(stable E-**1**)$_4$ (right).*

The binding constants were determined by NMR titrations at 20 °C. Titration of a stock solution of Pd_2(stable *Z*-**1**)$_4$ (c = 3.0×10^{-4} M) or Pd_2(stable *E*-**1**)$_4$ (*c* = 2.7×10^{-4} M) with a stock solution of tetrabutylammonium tosylate (c = 4.0×10^{-3} M) in a 1:1 mixture of CD_2Cl_2 and CD_3CN that contained the guest in the same concentration to exclude dilution effects was performed. The chemical shifts of $H_{a\text{-}d}$ (Pd_2(stable *Z*-**1**)$_4$) and $H_{a,c\text{-}e}$ (Pd_2(stable *E*-**1**)$_4$) were plotted against the host to guest ratio and fitted against a 1:1 binding model using BindFit software (Figure S3).[2] Binding constants of 1604 ± 39 M^{-1} for Pd_2(stable *Z*-**1**)$_4$ and 1758 ± 39 M^{-1} for Pd_2(stable *E*-**1**)$_4$ were obtained.

[2] P. Thordarson, BindFit v0.5, apps.supramolecular.org

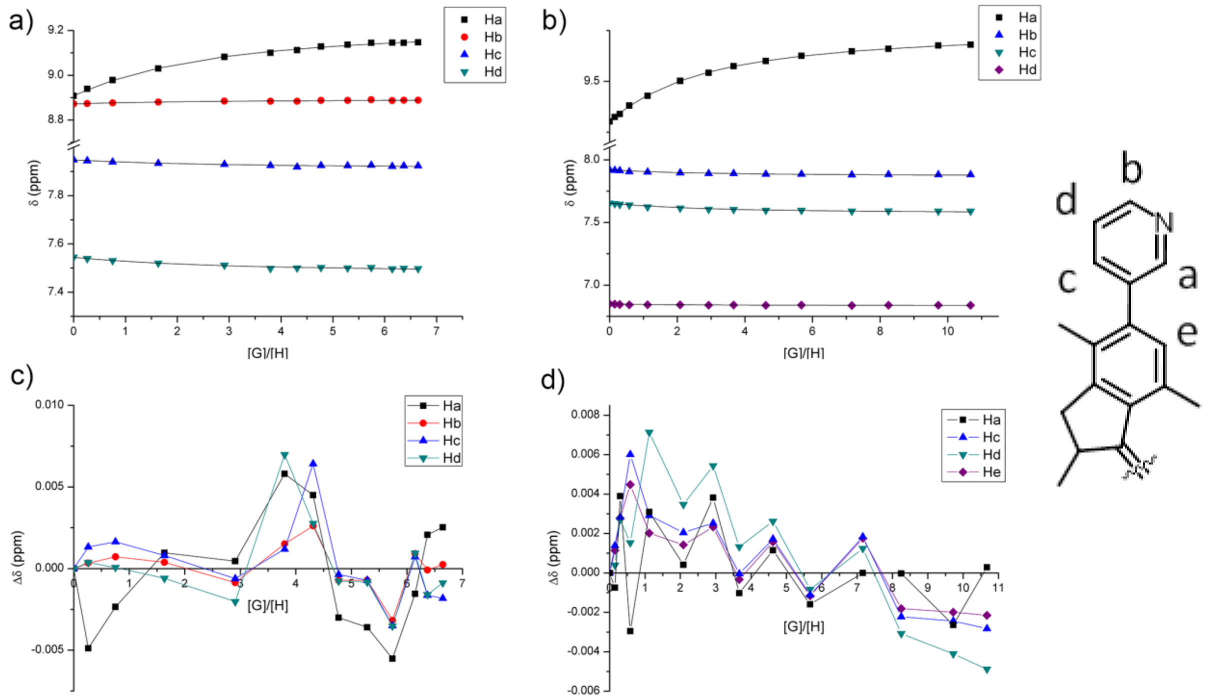

Figure S3: *Top: Fitting of ^{1}H-NMR titration data of Pd$_2$(stable Z-**1**)$_4$ (a) and Pd$_2$(stable Z-**1**)$_4$ (b) with tetrabutylammonium tosylate using protons H$_a$-H$_e$ as shown in figure 5.3. Bottom: residual plots of fitting of ^{1}H-NMR titration data of Pd$_2$(stable Z-**1**)$_4$ (c) and Pd$_2$(stable Z-**1**)$_4$ (d).*

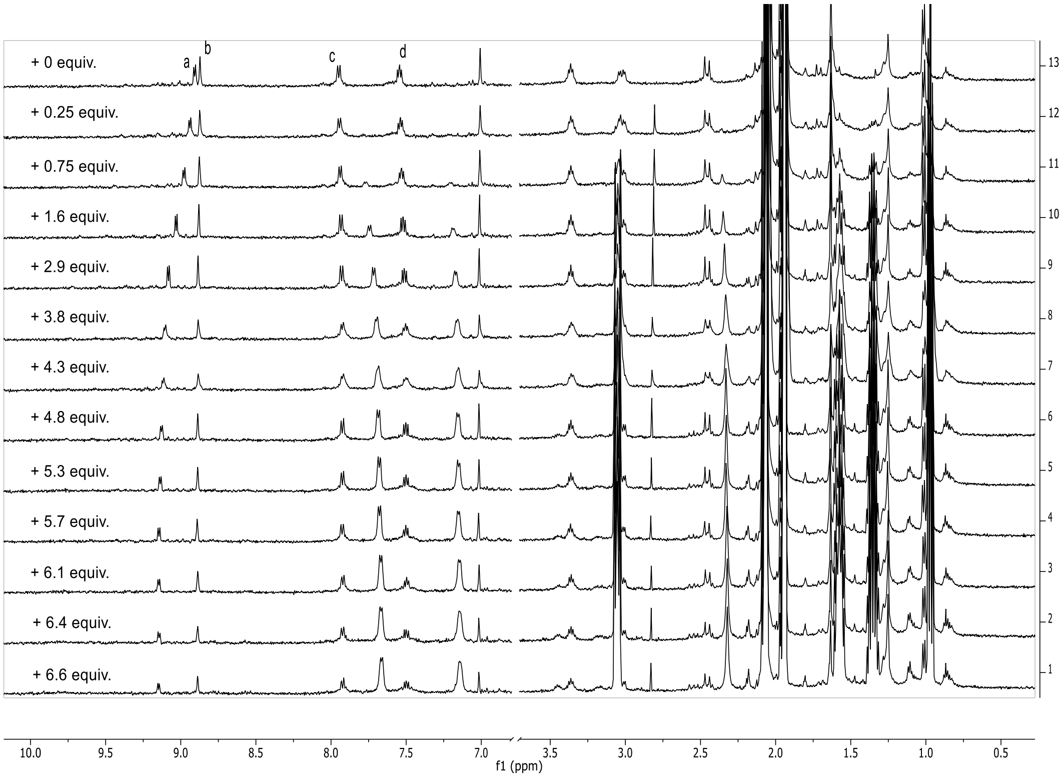

Figure S4: *Changes in the ^{1}H NMR signals of Pd$_2$(stable Z-**1**)$_4$ upon stepwise addition of TBAOTs*

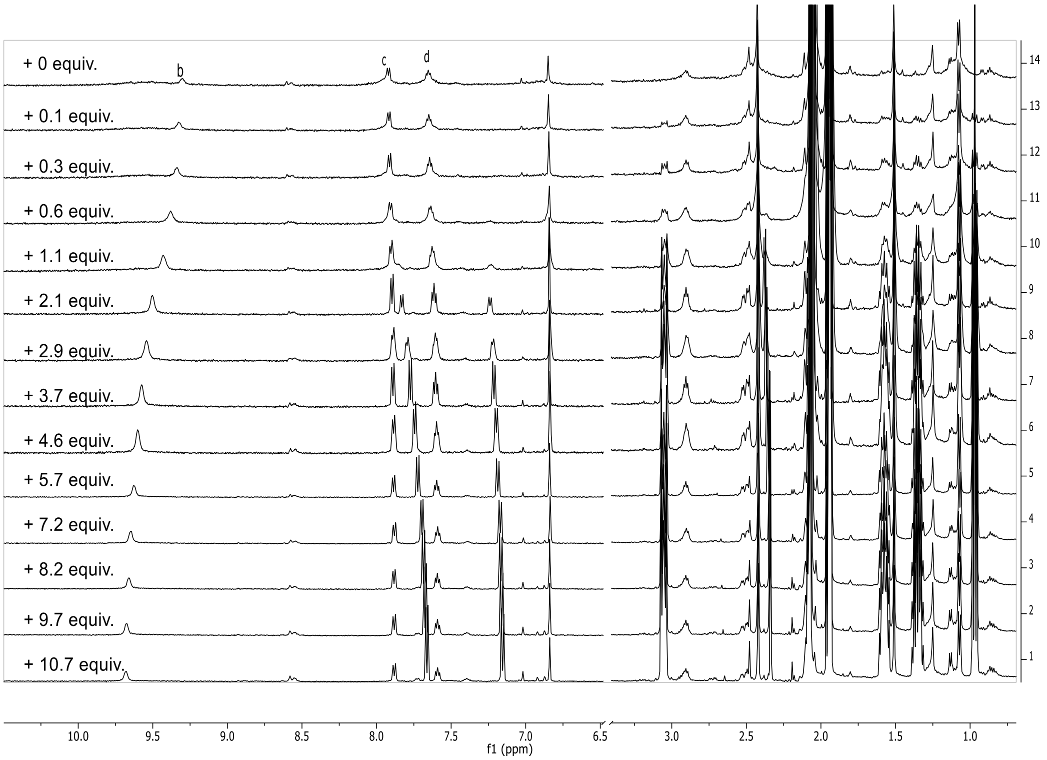

Figure S5: *Changes in the ^{1}H NMR signals of Pd$_2$(stable E-**1**)$_4$ upon stepwise addition of TBAOTs*

V. Photochemical and thermal isomerization studies

Switching cycle of ligand 1

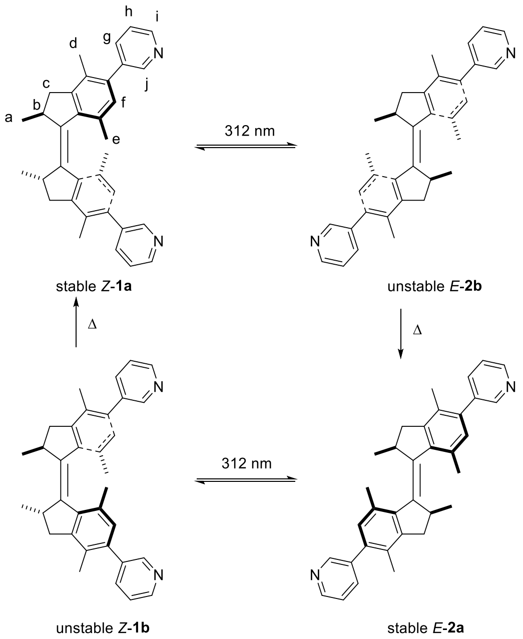

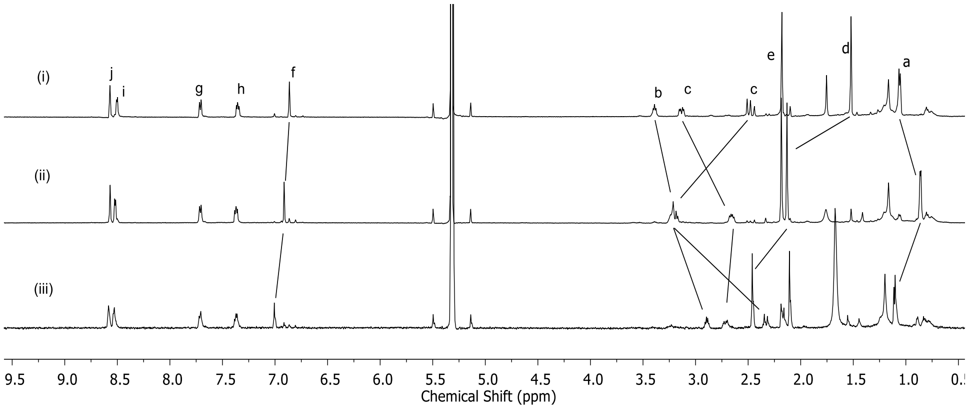

Figure S6: *^{1}H NMR spectrum of switching cycle of ligand stable Z-**1** in CD_2Cl_2 at −55 °C. i) Stable Z-**1**. ii) PSS 312, unstable E-**1**. iii) THI, stable E-**1**.*

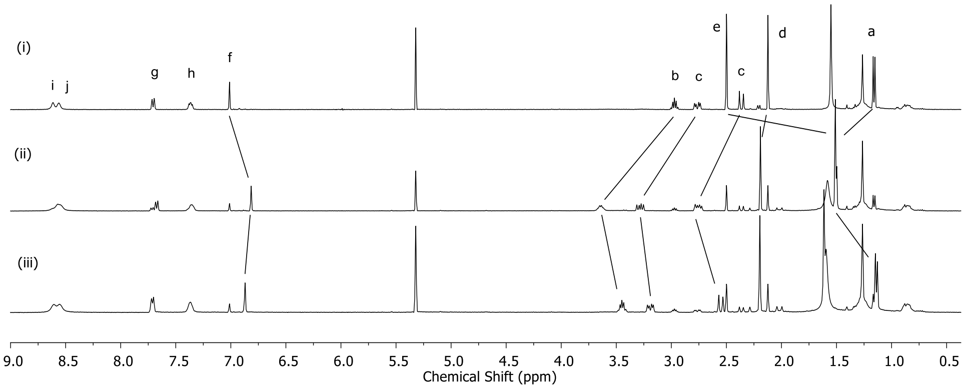

Figure S7: *^{1}H NMR spectra of switching cycle of ligand stable E-**1** in CD_2Cl_2. i) stable E-**1** ii) PSS 312 nm, unstable Z-**1**. iii) THI, stable Z-**1**.*

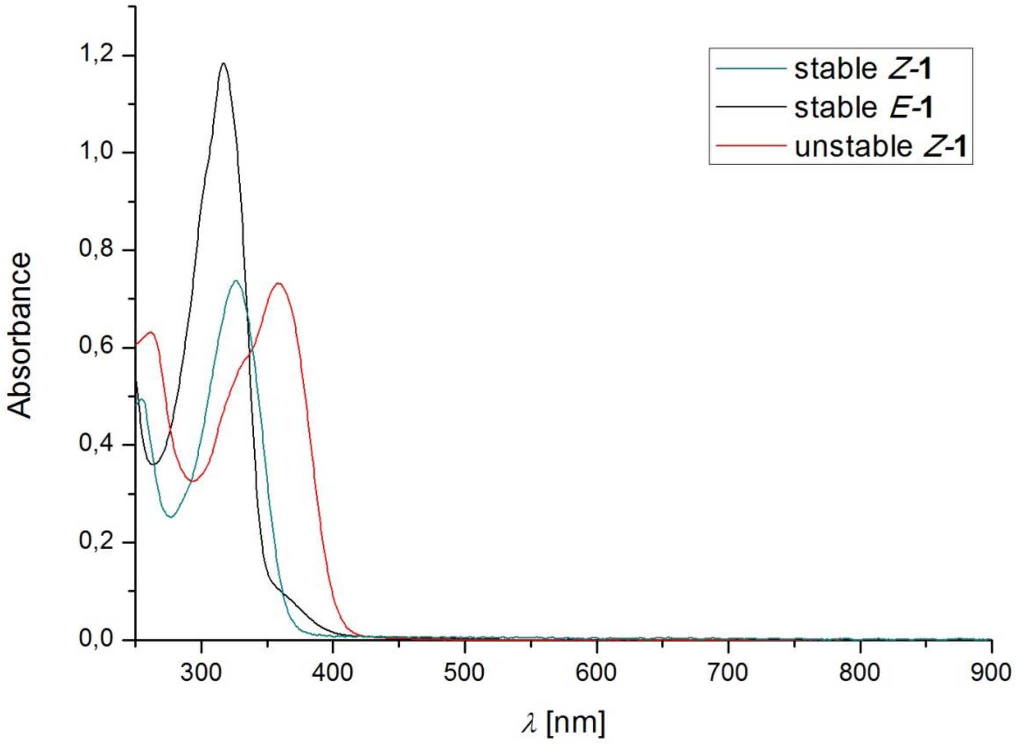

Figure S8: *UV–vis spectra of switching cycle of ligand isomers in heptane, c = 3 × 10^{-5} M.*

Isomerization of cage complexes

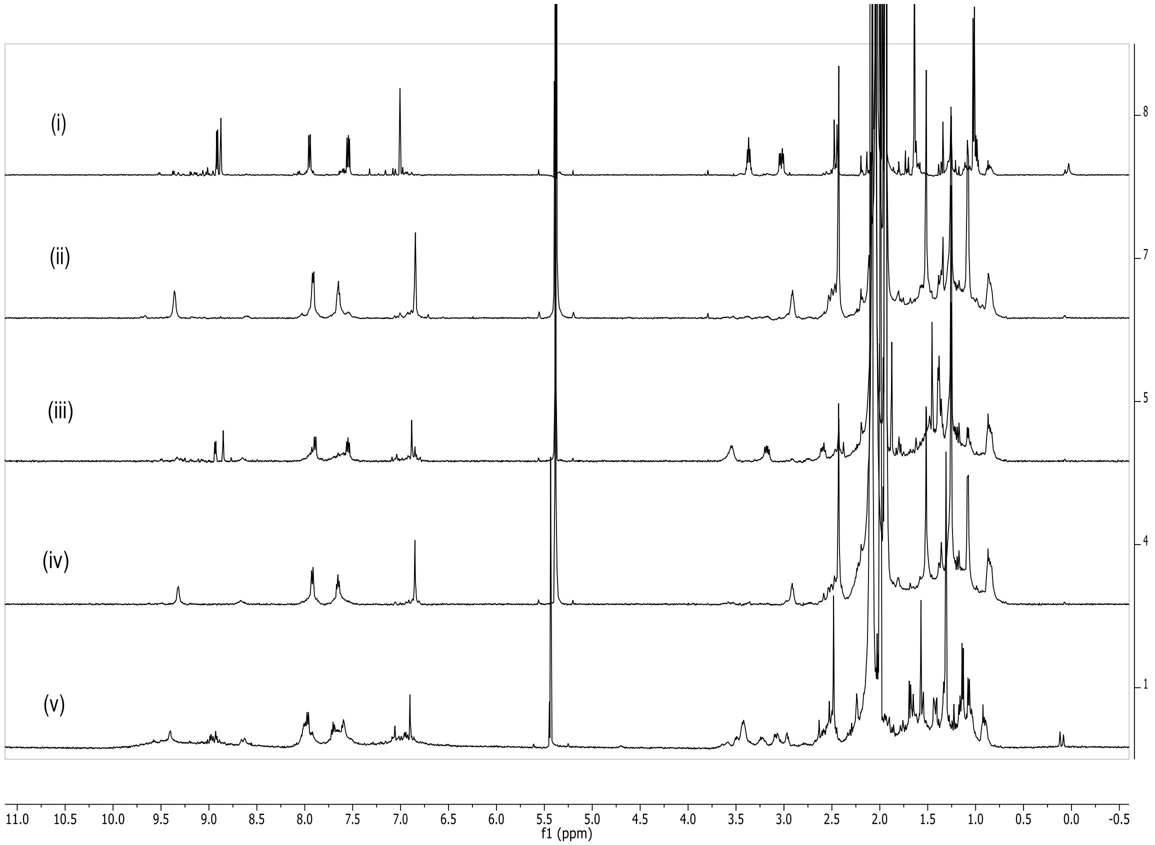

Figure S9: *Stacked ^{1}H-NMR spectra (CD_3CN/CD_2Cl_2 1:1, c = 5.7×10^{-4} M) of i: Pd_2(stable Z-**1**)$_4$, ii: Pd_2(stable E-**1**)$_4$ generated by irradiation of cage Pd_2(stable Z-**1**)$_4$ with 312 nm at −70 °C and subsequent warming to rt; iii: Pd_2(unstable Z-**1**)$_4$ generated by irradiation of Pd_2(stable E-**1**)$_4$ with 312 nm at −20 °C iv: Pd_2(stable E-**1**)$_4$ generated by irradiation of Pd_2(unstable Z-**1**)$_4$ with 365 nm at −20 °C. v: complex mixture obtained after thermal decomposition of Pd_2(unstable Z-**1**)$_4$.*

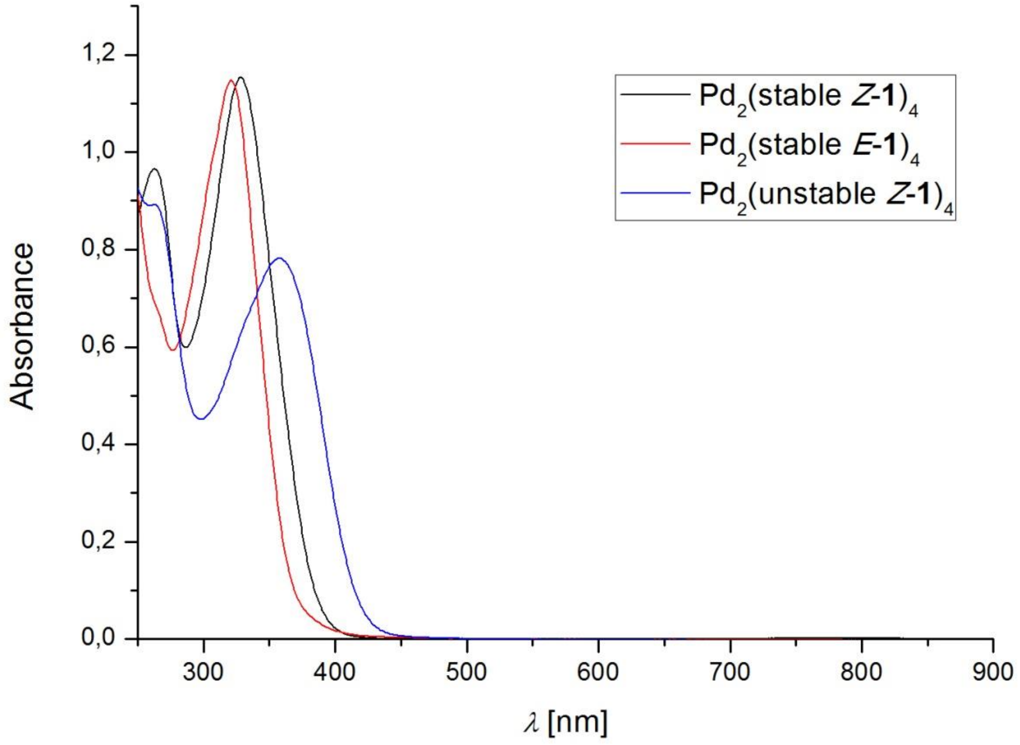

Figure S10: *UV–is spectra of switching cycle of ligand isomers in acetonitrile, c = 1.1 × 10⁻⁵ M.*

VI. X-ray crystallography

A single crystal of Pd_2(stable *E*-**1**)$_4$ formed from a racemic mixture of ligand *E*-**1** suitable for X-ray structure determination was grown by vapor diffusion of a 1:1 mixture of benzene and diethyl ether into a solution of the cage in a 1:1 mixture of acetonitrile and chloroform. Refinement of the structure indicated a significant amount of smeared out electron density, suggesting the presence of severely disordered solvent and/or anions. No satisfactory model was found to describe this density. Its contribution was removed using the PLATON/SQUEEZE routine,[3] which resulted in a solvent accessible volume of 2514 Å^3 containing 650 electrons. It is likely that this volume contains also disordered anions, as required for charge balance.

Formula	C_{69} $H_{69.5}$ $Cl_{0.5}$ N_5 $O_{1.5}$ $Pd_{0.5}$
M_r	1116.92
Cryst syst	Tetragonal
Color, habit	colourless, platelet
Size	.09 x 0.07 x 0.01
Space group	P 4/n
a (Å)	18.8017
b (Å)	18.8017
c (Å)	21.2886
V (Å^3)	7525.6
Z	4
ρ	0.986
μ	2.439
F(000)	2340
Temp (K)	100
θ range (deg)	3.136 - 65.212
Data collected (h,k,l)	-13:21, -22:22, -25:25
No. of rflns collected	40448
No. of indpndt reflns	6444
Observed rflns	5358
R(F) (%)	4.52
wR(F^2) (%)	11.23
GooF	1.093
Weighting a,b	0.0459, 12.4612
Params refined	365
Restraints	0
Min max resid dens	-0.668, 1.102

[3] Spek, A. L. (2015). Acta Cryst. C71, 9-18.

VII. Computational details

Computational studies were performed using the Gaussian09 program.[4] Geometries were initially optimized at the semi-empirical PM6 level to obtain the global minima. The geometries were further optimized at the B3LYP/6-31G(d) level for C,H,N and LANL2DZ with ECP for Pd in the gas phase without counterions, using a superfine grid and tight convergence criteria. Subsequent frequency analysis was performed to ensure a global minimum was achieved.

Isomer	Relative Gibbs free energy (kJ mol^{-1})
$(S,S)_4$	0
$(S,S)_3(R,R)$	+18.5
$(S,S)_2(R,R)_2$	+27.4
(*S,S*)(*R,R*)(*S,S*)(*R,R*)	+32.4

*Table S1: Relative energies of diastereomers of Pd_2(stable Z-**1**)$_4$*

Isomer	Relative Gibbs free energy (kJ mol^{-1})
$(S,S)_4$	0
$(S,S)_3(R,R)$	+60.9
$(S,S)_2(R,R)_2$	+75.8
(*S,S*)(*R,R*)(*S,S*)(*R,R*)	+113.8

*Table S2: Relative energies of diastereomers of Pd_2(stable E-**1**)$_4$*

[4] Gaussian 09, Revision D.01, Frisch, M. J.; Trucks, G. W.; Schlegel, H. B.; Scuseria, G. E.; Robb, M. A.; Cheeseman, J. R.; Scalmani, G.; Barone, V.; Mennucci, B.; Petersson, G. A.; Nakatsuji, H.; Caricato, M.; Li, X.; Hratchian, H. P.; Izmaylov, A. F.; Bloino, J.; Zheng, G.; Sonnenberg, J. L.; Hada, M.; Ehara, M.; Toyota, K.; Fukuda, R.; Hasegawa, J.; Ishida, M.; Nakajima, T.; Honda, Y.; Kitao, O.; Nakai, H.; Vreven, T.; Montgomery, J. A., Jr.; Peralta, J. E.; Ogliaro, F.; Bearpark, M.; Heyd, J. J.; Brothers, E.; Kudin, K. N.; Staroverov, Keith, T.; V. N.; Kobayashi, R.; Normand, J.; Raghavachari, K.; Rendell, A.; Burant, J. C.; Iyengar, S. S.; Tomasi, J.; Cossi, M.; Rega, N.; Millam, J. M.; Klene, M.; Knox, J. E.; Cross, J. B.; Bakken, V.; Adamo, C.; Jaramillo, J.; Gomperts, R.; Stratmann, R. E.; Yazyev, O.; Austin, A. J.; Cammi, R.; Pomelli, C.; Ochterski, J. W.; Martin, R. L.; Morokuma, K.; Zakrzewski, V. G.; Voth, G. A.; Salvador, P.; Dannenberg, J. J.; Dapprich, S.; Daniels, A. D.; Farkas, O.; Foresman, J. B.; Ortiz, J. V.; Cioslowski, J.; Fox, D. J. Gaussian, Inc., Wallingford CT, **2013**.

Pd_2(stable *Z*-1)$_4$

(*S*,*S*)$_4$
0 kJ mol^{-1}

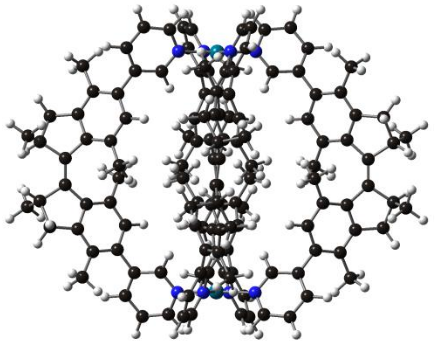

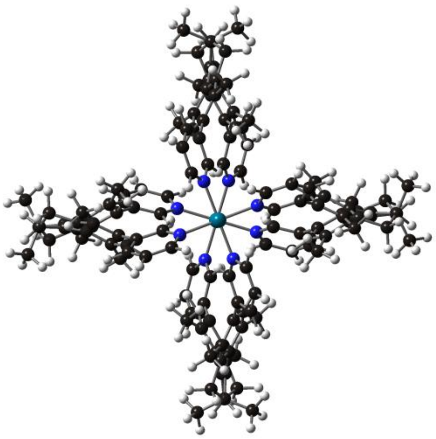

(*S*,*S*)$_3$(*R*,*R*)
+18.5 kJ mol^{-1}

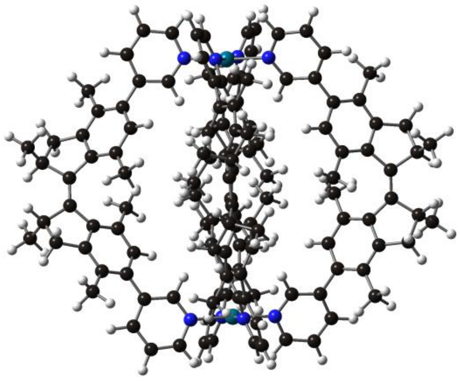

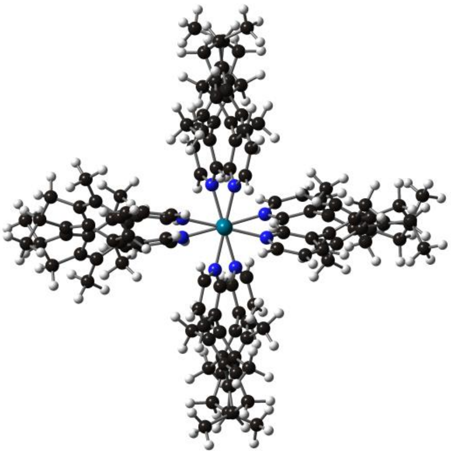

$(S,S)_2(R,R)_2$
+27.4 kJ mol^{-1}

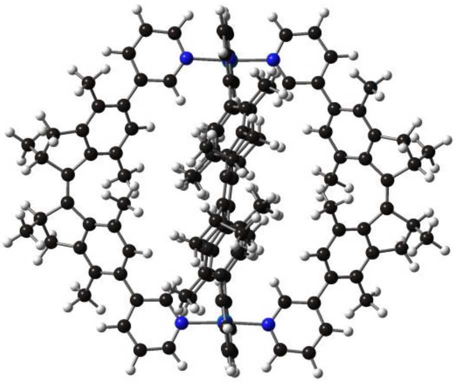

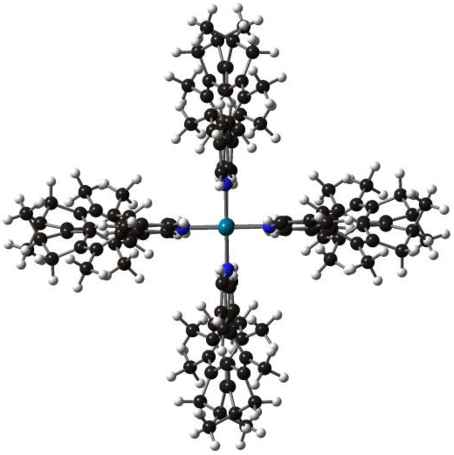

$(S,S)(R,R)(S,S)(R,R)$
+32.4 kJ mol^{-1}

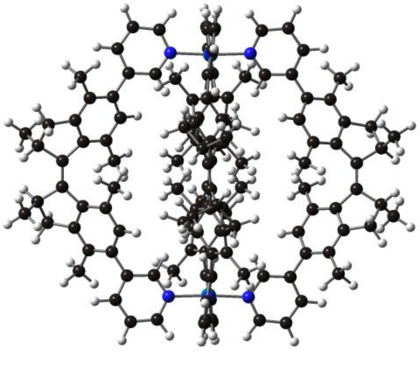

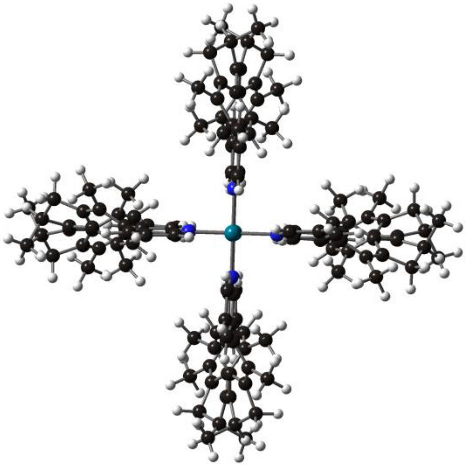

Pd_2(stable *E*-1)$_4$

(*S*,*S*)$_4$

0 kJ mol^{-1}

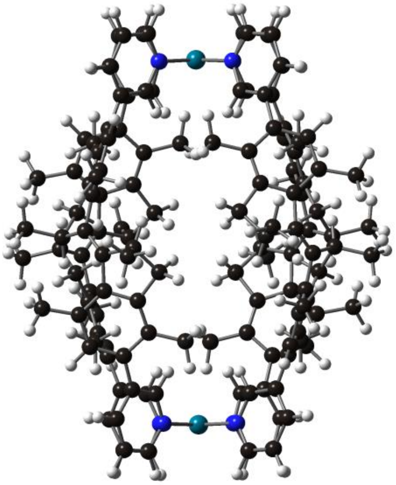

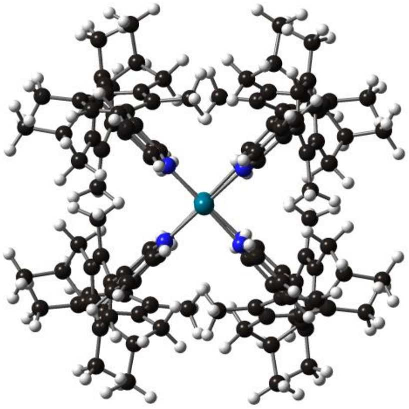

(*S*,*S*)$_3$(*R*,*R*)

+60.9 kJ mol^{-1}

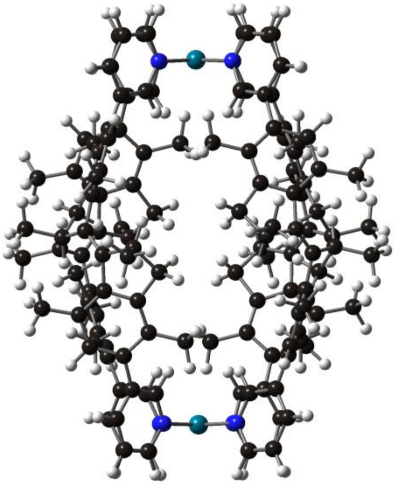

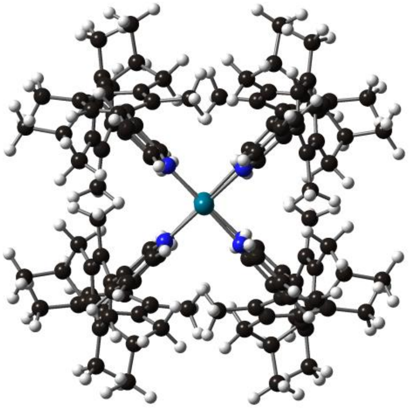

$(S,S)_2(R,R)_2$
+75.8 kJ mol^{-1}

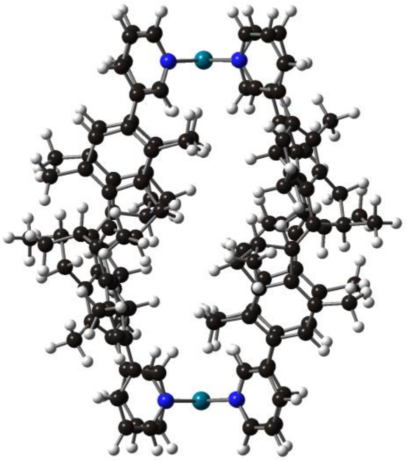

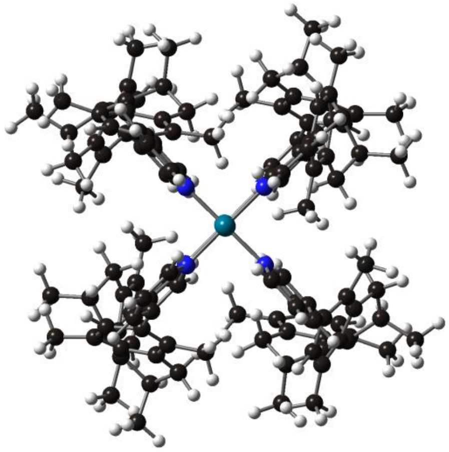

$(S,S)(R,R)(S,S)(R,R)$
+113.8 kJ mol^{-1}

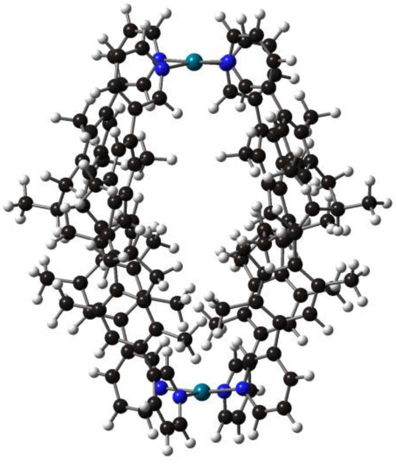

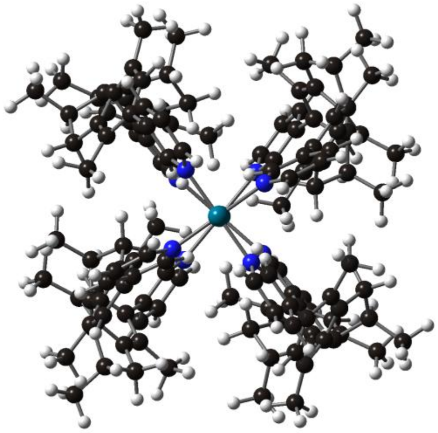

Pd_2(unstable *Z*-1)$_4$

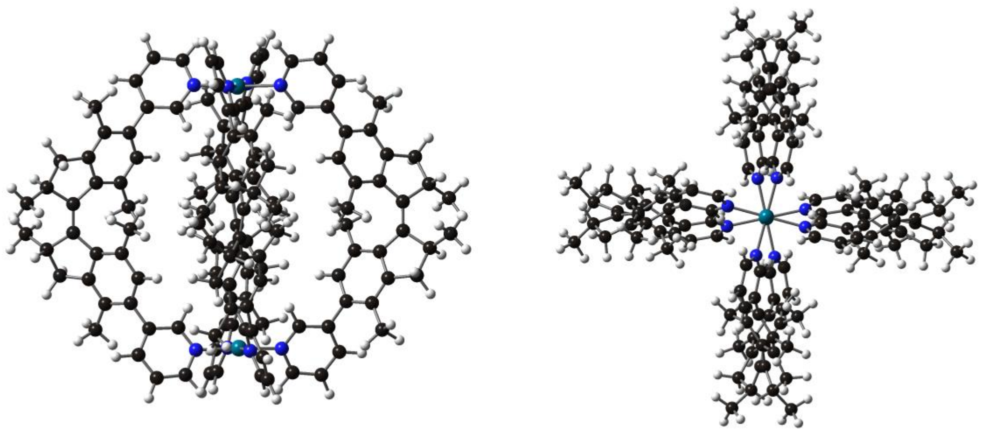

VIII. Carthesian coordinates of DFT optimized structures

Cartesian coordinates of all the optimized structures are given, including the sum of electronic and thermal free energies in hartree.

Pd_2(stable *Z*-1)$_4$

(*S,S*)$_4$

Sum of electronic and thermal free energies: -5952.794672

H	-0.417613000	2.408444000	3.939692000
C	7.362026000	1.467379000	3.841119000
H	8.266592000	1.350112000	4.428528000
C	0.316766000	2.297502000	4.745649000
C	7.243875000	0.808008000	2.623362000
H	8.034382000	0.171939000	2.241854000
H	2.679973000	1.455005000	3.805282000
C	6.306585000	2.255771000	4.291550000
C	2.722108000	2.514278000	4.052053000
N	6.134084000	0.929175000	1.864896000
H	6.375145000	2.759300000	5.250227000

H	-0.188806000	2.589525000	5.671625000
C	1.557843000	3.128699000	4.528953000
C	5.137376000	2.382854000	3.519991000
C	5.111410000	1.694595000	2.299162000
H	0.577070000	1.236074000	4.819314000
C	3.962214000	3.170963000	3.971652000
H	1.018349000	7.357545000	6.429288000
H	4.251872000	1.774467000	1.643220000
C	1.657636000	4.489163000	4.894442000
C	0.674651000	5.408833000	5.506801000
C	4.079361000	4.515780000	4.401129000
C	2.919805000	5.126746000	4.879933000
C	1.508367000	6.384279000	6.351806000
H	5.186728000	6.333621000	4.045977000
C	5.372319000	5.301734000	4.359970000
C	2.813251000	6.494620000	5.515093000
H	0.880383000	5.705866000	8.341766000
H	3.682877000	6.750355000	6.129152000
H	6.101477000	4.875345000	3.665765000
C	1.801580000	5.858691000	7.770434000
H	2.420875000	6.574899000	8.321323000
H	5.849110000	5.357550000	5.347693000
H	2.714347000	7.279620000	4.751288000
H	2.335844000	4.902200000	7.736237000
H	-1.018615000	6.463737000	7.326981000
H	-5.186851000	4.075404000	6.313544000
H	-2.714407000	4.785208000	7.256418000
H	-2.421365000	8.352195000	6.536156000
C	-1.508658000	6.381964000	6.354081000
C	-5.372438000	4.384916000	5.280301000
C	-1.802039000	7.798256000	5.822331000
H	-0.880905000	8.369012000	5.666991000
C	-2.813447000	5.545581000	6.468110000
H	-6.101548000	3.688831000	4.856901000
H	-4.251608000	1.652247000	1.766038000
C	-2.919985000	4.904455000	5.103018000
C	-5.111231000	2.307681000	1.682965000
C	-4.079476000	4.422785000	4.494187000
N	-6.133863000	1.869549000	0.919694000
C	-0.674898000	5.532795000	5.382295000
C	-1.657857000	4.916395000	4.465316000
C	-3.962276000	3.987398000	3.151276000
C	-5.137357000	3.531836000	2.365283000
H	-5.849293000	5.372841000	5.331827000
C	-2.722232000	4.065223000	2.494160000
C	-1.558052000	4.545078000	3.106439000
H	0.417668000	3.953427000	2.388892000
H	-2.336332000	7.759842000	4.866016000

H	-3.683135000	6.160641000	6.721208000
C	-6.306654000	4.302626000	2.234455000
C	-7.362007000	3.848272000	1.448193000
C	-0.317059000	4.758548000	2.274290000
H	-2.680101000	3.813804000	1.435980000
H	0.188108000	5.686047000	2.562166000
H	-6.375340000	5.263697000	2.733389000
H	-8.266630000	4.435009000	1.328035000
H	-0.577360000	4.827333000	1.212536000
H	-8.034146000	-2.242656000	-0.160641000
C	-7.243695000	-2.627338000	-0.794865000
N	-6.133848000	-1.869562000	-0.919773000
C	-7.361982000	-3.848287000	-1.448290000
C	-5.111207000	-2.307692000	-1.683033000
H	-8.266605000	-4.435025000	-1.328143000
H	-4.251585000	-1.652253000	-1.766097000
C	-6.306619000	-4.302637000	-2.234542000
C	-5.137323000	-3.531844000	-2.365354000
H	-2.680074000	-3.813814000	-1.436030000
H	-6.375298000	-5.263705000	-2.733480000
N	6.134114000	-0.929198000	-1.864822000
C	-2.722196000	-4.065228000	-2.494211000
C	-3.962233000	-3.987400000	-3.151338000
H	4.251888000	-1.774470000	-1.643187000
C	5.111441000	-1.694607000	-2.299111000
H	8.034428000	-0.171982000	-2.241739000
H	0.417705000	-3.953429000	-2.388915000
C	7.243923000	-0.808043000	-2.623264000
H	-0.417553000	-2.408404000	-3.939770000
H	-0.577334000	-4.827342000	-1.212573000
H	-6.101496000	-3.688838000	-4.856973000
C	-0.317023000	-4.758551000	-2.274324000
H	2.680040000	-1.454997000	-3.805289000
C	-1.558010000	-4.545078000	-3.106483000
C	-4.079421000	-4.422782000	-4.494252000
C	0.316848000	-2.297474000	-4.745709000
C	-5.372377000	-4.384912000	-5.280375000
H	0.577165000	-1.236049000	-4.819373000
C	2.722171000	-2.514270000	-4.052054000
C	5.137428000	-2.382869000	-3.519938000
C	7.362095000	-1.467418000	-3.841017000
H	0.188147000	-5.686049000	-2.562200000
C	1.557910000	-3.128683000	-4.528976000
H	-0.188703000	-2.589496000	-5.671696000
H	-5.186786000	-4.075387000	-6.313613000
C	-1.657801000	-4.916386000	-4.465363000
H	-5.849225000	-5.372840000	-5.331915000
C	-2.919924000	-4.904446000	-5.103076000

C	3.962269000	-3.170967000	-3.971623000
C	6.306656000	-2.255800000	-4.291470000
H	8.266676000	-1.350163000	-4.428406000
C	1.657698000	-4.489150000	-4.894455000
C	4.079414000	-4.515787000	-4.401094000
C	-0.674833000	-5.532778000	-5.382337000
C	-2.813373000	-5.545565000	-6.468170000
H	6.375233000	-2.759333000	-5.250144000
H	-2.336265000	-7.759834000	-4.866084000
C	2.919862000	-5.126743000	-4.879919000
C	0.674718000	-5.408816000	-5.506827000
H	-2.714329000	-4.785189000	-7.256473000
H	6.101509000	-4.875366000	-3.665684000
H	-3.683057000	-6.160626000	-6.721278000
C	5.372365000	-5.301749000	-4.359908000
C	-1.508582000	-6.381943000	-6.354134000
C	-1.801964000	-7.798241000	-5.822395000
H	5.186760000	-6.333636000	-4.045923000
C	2.813311000	-6.494618000	-5.515080000
H	2.714385000	-7.279617000	-4.751277000
C	1.508442000	-6.384267000	-6.351818000
H	-0.880828000	-8.368993000	-5.667050000
H	-2.421282000	-8.352177000	-6.536229000
H	5.849179000	-5.357563000	-5.347619000
H	-1.018530000	-6.463710000	-7.327031000
H	2.335954000	-4.902192000	-7.736233000
C	1.801686000	-5.858679000	-7.770440000
H	1.018420000	-7.357530000	-6.429311000
H	3.682945000	-6.750359000	-6.129123000
H	0.880499000	-5.705849000	-8.341789000
H	2.420986000	-6.574891000	-8.321319000
H	5.186767000	-4.046018000	6.333632000
H	2.714376000	-4.751349000	7.279622000
C	5.372356000	-4.360011000	5.301745000
H	6.101518000	-3.665810000	4.875356000
H	5.849140000	-5.347736000	5.357559000
H	3.682902000	-6.129210000	6.750345000
C	2.813280000	-5.515145000	6.494614000
H	-0.880908000	-5.667053000	8.368979000
H	1.018370000	-6.429340000	7.357524000
C	4.079398000	-4.401161000	4.515790000
C	-1.802034000	-5.822395000	7.798211000
C	1.508391000	-6.351853000	6.384261000
C	2.919840000	-4.879971000	5.126746000
H	-2.421364000	-6.536227000	8.352137000
H	-2.336333000	-4.866083000	7.759796000
H	6.375216000	-5.250199000	2.759349000
H	2.420892000	-8.321375000	6.574871000

H	8.266667000	-4.428463000	1.350185000
C	6.306644000	-4.291524000	2.255816000
C	7.362088000	-3.841072000	1.467440000
H	0.188122000	-2.562203000	5.686043000
H	-0.577350000	-1.212572000	4.827334000
C	1.801602000	-7.770479000	5.858665000
C	0.674683000	-5.506839000	5.408816000
C	3.962256000	-3.971668000	3.170978000
C	5.137419000	-3.519986000	2.382885000
C	7.243922000	-2.623315000	0.808067000
C	-0.317039000	-2.274323000	4.758541000
C	1.657674000	-4.894473000	4.489157000
C	-1.508633000	-6.354134000	6.381918000
C	-0.674867000	-5.382337000	5.532767000
H	-1.018585000	-7.327031000	6.463690000
H	8.034430000	-2.241793000	0.172010000
C	5.111440000	-2.299157000	1.694629000
N	6.134115000	-1.864869000	0.929222000
H	2.335868000	-7.736278000	4.902176000
H	0.880402000	-8.341806000	5.705834000
H	0.417697000	-2.388910000	3.953426000
C	-1.657824000	-4.465360000	4.916360000
C	-1.558024000	-3.106480000	4.545052000
C	2.722153000	-4.052065000	2.514285000
C	1.557886000	-4.528971000	3.128697000
H	4.251889000	-1.643230000	1.774491000
C	-2.813411000	-6.468164000	5.545519000
H	-3.683105000	-6.721272000	6.160568000
C	-2.919948000	-5.103069000	4.904403000
C	-2.722202000	-2.494204000	4.065190000
H	8.034459000	2.241746000	-0.171933000
H	-2.680075000	-1.436022000	3.813783000
H	2.680022000	-3.805278000	1.455017000
C	7.243957000	2.623278000	-0.807993000
C	0.316807000	-4.745650000	2.297498000
H	8.266726000	4.428412000	-1.350109000
C	7.362140000	3.841032000	-1.467366000
H	-2.714358000	-7.256466000	4.785142000
C	-4.079439000	-4.494240000	4.422728000
N	6.134141000	1.864846000	-0.929150000
C	-3.962242000	-3.151324000	3.987352000
H	-0.188770000	-5.671626000	2.589510000
H	-0.417567000	-3.939689000	2.408451000
H	-5.849256000	-5.331899000	5.372763000
C	5.111474000	2.299146000	-1.694560000
C	6.306705000	4.291497000	-2.255745000
H	0.577195000	4.819384000	-1.235999000
C	-5.372395000	-5.280360000	4.384841000

H	-6.375300000	-2.733446000	5.263661000
C	-5.137326000	-2.365330000	3.531795000
H	2.680081000	3.805316000	-1.454945000
H	0.577110000	-4.819304000	1.236069000
C	-6.306620000	-2.234508000	4.302592000
C	5.137470000	3.519975000	-2.382816000
H	6.375290000	5.250172000	-2.759277000
H	4.251914000	1.643231000	-1.774423000
C	2.722213000	4.052092000	-2.514217000
C	-5.111207000	-1.683009000	2.307644000
C	0.316883000	4.745724000	-2.297425000
H	-5.186802000	-6.313599000	4.075321000
H	-4.251588000	-1.766078000	1.652204000
C	-7.361977000	-1.448248000	3.848245000
C	3.962313000	3.971672000	-3.170911000
H	-6.101504000	-4.856958000	3.688757000
H	-0.417508000	3.939777000	-2.408363000
C	1.557952000	4.529010000	-3.128630000
H	-8.266597000	-1.328093000	4.434986000
H	-0.188678000	5.671707000	-2.589443000
N	-6.133843000	-0.919741000	1.869517000
C	-7.243688000	-0.794823000	2.627296000
H	2.335967000	7.736286000	-4.902121000
C	4.079460000	4.401153000	-4.515726000
H	-4.251565000	1.766073000	-1.652247000
C	1.657741000	4.894497000	-4.489095000
C	1.801702000	7.770491000	-5.858611000
H	-8.034134000	-0.160593000	2.242618000
H	6.101562000	3.665759000	-4.875300000
N	-6.133813000	0.919728000	-1.869595000
C	-5.111172000	1.683001000	-2.307703000
H	0.880508000	8.341828000	-5.705782000
C	5.372415000	4.359979000	-5.301684000
C	2.919907000	4.879975000	-5.126684000
H	5.849223000	5.347695000	-5.357492000
H	-5.186755000	6.313596000	-4.075355000
H	2.420999000	8.321380000	-6.574817000
H	-2.714304000	7.256479000	-4.785130000
H	0.417757000	2.388927000	-3.953397000
C	-7.243644000	0.794808000	-2.627394000
C	0.674757000	5.506864000	-5.408761000
C	-5.137273000	2.365325000	-3.531853000
H	-8.034096000	0.160575000	-2.242730000
C	-3.962185000	3.151326000	-3.987387000
H	-6.101459000	4.856949000	-3.688816000
C	-2.722141000	2.494210000	-4.065210000
C	-5.372340000	5.280358000	-4.384887000
C	-4.079379000	4.494243000	-4.422756000

C	-1.557957000	3.106495000	-4.545047000
C	1.508477000	6.351868000	-6.384205000
C	-1.657756000	4.465377000	-4.916344000
C	-2.919883000	5.103080000	-4.904406000
C	2.813355000	5.515144000	-6.494555000
H	-2.680013000	1.436026000	-3.813809000
C	-0.674791000	5.382362000	-5.532728000
C	-0.316965000	2.274344000	-4.758525000
H	3.682985000	6.129197000	-6.750289000
C	-2.813339000	6.468181000	-5.545513000
H	5.186817000	4.045998000	-6.333573000
C	-7.361914000	1.448235000	-3.848344000
C	-6.306552000	2.234500000	-4.302671000
H	0.188209000	2.562235000	-5.686016000
C	-1.508544000	6.354161000	-6.381887000
H	-1.018498000	7.327062000	-6.463644000
H	-5.849184000	5.331902000	-5.372815000
H	1.018457000	6.429359000	-7.357470000
H	-3.683023000	6.721288000	-6.160577000
H	-8.266524000	1.328079000	-4.435100000
H	-0.577270000	1.212593000	-4.827332000
H	2.714442000	4.751345000	-7.279560000
H	-6.375218000	2.733440000	-5.263739000
C	-1.801915000	5.822433000	-7.798191000
H	-2.336209000	4.866117000	-7.759795000
H	-0.880776000	5.667100000	-8.368941000
H	-2.421236000	6.536266000	-8.352124000
Pd	-6.086900000	-0.000007000	-0.000039000
Pd	6.087186000	-0.000011000	0.000036000
C	-7.243711000	2.627323000	0.794772000
H	-8.034153000	2.242640000	0.160539000

(*S,S*)$_3$(*R,R*)
Sum of electronic and thermal free energies: -5952.787609

C	4.777713000	-5.817628000	-0.257635000
C	4.569209000	-4.582629000	0.405015000
C	3.321424000	-4.302727000	0.989151000
C	2.237234000	-5.187484000	0.946746000
C	2.426897000	-6.395730000	0.240601000
C	3.700963000	-6.704241000	-0.289606000
H	3.215346000	-3.389163000	1.571239000
C	0.994977000	-4.877546000	1.745918000
H	1.215125000	-4.137993000	2.523258000
H	0.612021000	-5.780250000	2.232832000
H	0.178336000	-4.484568000	1.129571000
C	6.081278000	-6.207846000	-0.921093000
H	6.673248000	-6.888842000	-0.295059000
H	6.709393000	-5.343852000	-1.154147000

H	5.891463000	-6.736315000	-1.860465000
C	3.715143000	-8.127757000	-0.798901000
C	1.555625000	-7.580384000	0.086724000
C	2.517062000	-8.771511000	-0.046945000
H	4.659620000	-8.644508000	-0.600070000
H	3.549330000	-8.162408000	-1.885497000
C	2.960466000	-9.349513000	1.310993000
H	2.073484000	-9.571446000	-0.644196000
H	3.453876000	-8.586859000	1.924447000
H	3.665446000	-10.174518000	1.161520000
H	2.110020000	-9.737013000	1.881019000
C	5.663952000	-3.598854000	0.599845000
C	6.913269000	-3.955491000	1.139510000
C	7.891262000	-2.986992000	1.346341000
C	7.614551000	-1.664761000	1.020354000
N	6.424207000	-1.306530000	0.496194000
C	5.478309000	-2.244777000	0.287455000
H	4.547800000	-1.907545000	-0.155548000
H	7.105260000	-4.987247000	1.414215000
H	8.856730000	-3.244238000	1.769008000
H	8.340679000	-0.876044000	1.180128000
H	0.744973000	-5.798906000	-2.181354000
H	-3.987565000	-2.841053000	0.131300000
C	-4.808495000	-3.391478000	-0.314389000
N	-5.944169000	-2.700679000	-0.545276000
H	-2.595413000	-9.427755000	0.703668000
H	-1.425187000	-9.153485000	-1.824233000
C	0.172429000	-4.993777000	-1.709624000
H	-4.933152000	-8.168615000	0.364896000
H	-0.199409000	-4.332559000	-2.499717000
C	-0.975794000	-5.554013000	-0.906343000
C	-2.227047000	-4.928345000	-0.966268000
C	0.206058000	-7.728458000	-0.006702000
H	-2.320154000	-4.023594000	-1.564288000
C	-0.900666000	-6.762935000	-0.179779000
C	-3.387043000	-5.464794000	-0.381049000
C	-4.672864000	-4.756538000	-0.604789000
C	-7.011489000	-3.334209000	-1.073817000
C	-3.323583000	-6.702886000	0.305368000
C	-2.079342000	-7.331829000	0.355786000
C	-4.513606000	-7.357171000	0.974474000
H	-5.320587000	-6.649494000	1.182423000
C	-1.785889000	-8.714941000	0.891147000
C	-0.779929000	-9.780586000	-1.198269000
H	-7.899983000	-2.739803000	-1.251992000
C	-0.475239000	-9.096968000	0.148768000
H	0.881401000	-4.421938000	-1.100240000
C	-5.798124000	-5.400544000	-1.151063000

C	-6.970880000	-4.688360000	-1.382867000
H	0.135541000	-9.984714000	-1.762693000
H	-1.290058000	-10.735887000	-1.033487000
H	-1.618771000	-8.692789000	1.977860000
H	-4.219300000	-7.806109000	1.928239000
H	-5.741830000	-6.452481000	-1.410143000
H	-7.845045000	-5.167759000	-1.810626000
H	0.129543000	-9.771500000	0.759178000
H	-4.547797000	1.907506000	-0.156045000
N	-6.424202000	1.306663000	0.495862000
C	-5.478305000	2.244855000	0.286874000
H	-1.215116000	4.138667000	2.522178000
H	-8.340672000	0.876357000	1.179914000
C	-7.614544000	1.665031000	1.019932000
H	7.845038000	5.167279000	-1.812016000
H	-3.215339000	3.389580000	1.570357000
C	-0.994970000	4.878012000	1.744640000
C	6.970875000	4.687994000	-1.384124000
H	5.741829000	6.452111000	-1.411858000
C	-3.321418000	4.302991000	0.988028000
H	-0.178332000	4.484869000	1.128396000
C	-5.663946000	3.599014000	0.598911000
C	5.798122000	5.400242000	-1.152502000
H	-0.612011000	5.780847000	2.231312000
C	-2.237229000	5.187738000	0.945389000
C	-7.891253000	2.987348000	1.345574000
C	-4.569204000	4.582738000	0.403820000
C	7.011483000	3.333925000	-1.074718000
H	7.899975000	2.739470000	-1.252742000
H	5.320591000	6.649812000	1.180660000
H	-3.453874000	8.587368000	1.922194000
C	-6.913261000	3.955792000	1.138486000
H	1.618780000	8.693321000	1.975562000
C	4.672864000	4.756382000	-0.606051000
C	4.513610000	7.357434000	0.972525000
H	-2.110018000	9.737510000	1.878463000
C	-2.960464000	9.349861000	1.308540000
C	-2.426893000	6.395798000	0.238925000
H	4.933156000	8.168716000	0.362730000
H	-8.856721000	3.244705000	1.768175000
C	-4.777709000	5.817561000	-0.259156000
C	3.323585000	6.702972000	0.303594000
C	3.387044000	5.464698000	-0.382494000
N	5.944166000	2.700537000	-0.546003000
C	2.079345000	7.331928000	0.353849000
C	4.808494000	3.391398000	-0.315292000
C	-3.700962000	6.704167000	-0.291362000
C	1.785894000	8.715182000	0.888843000

C	2.227045000	4.928092000	-0.967565000
H	-3.665444000	10.174827000	1.158851000
H	-6.673248000	6.888760000	-0.296853000
C	-1.555623000	7.580411000	0.084734000
C	0.900666000	6.762891000	-0.181559000
C	-6.081277000	6.207601000	-0.922711000
C	0.975792000	5.553775000	-0.907802000
H	-5.891468000	6.735827000	-1.862222000
H	2.320150000	4.023182000	-1.565346000
C	-0.206057000	7.728460000	-0.008736000
H	-7.105251000	4.987620000	1.412920000
H	-0.881401000	4.421646000	-1.101386000
H	4.219306000	7.806626000	1.926171000
C	-2.517062000	8.771502000	-0.049247000
H	2.595417000	9.427946000	0.701170000
C	-0.172435000	4.993324000	-1.710927000
C	0.475241000	9.097012000	0.146367000
H	-0.129539000	9.771706000	0.756600000
C	-3.715143000	8.127549000	-0.801032000
H	3.987565000	2.841092000	0.130547000
H	0.199401000	4.331896000	-2.500846000
H	-4.659620000	8.644351000	-0.602336000
H	-6.709392000	5.343546000	-1.155537000
H	-2.073485000	9.571280000	-0.646709000
H	-0.744982000	5.798326000	-2.182868000
H	-3.549332000	8.161914000	-1.887637000
C	0.779926000	9.780271000	-1.200852000
H	1.425184000	9.153004000	-1.826651000
H	1.290053000	10.735617000	-1.036327000
H	-0.135545000	9.984247000	-1.765329000
H	8.353309000	0.238268000	-1.925058000
H	8.677585000	-0.021612000	-4.387069000
C	7.507794000	0.147895000	-2.597914000
C	7.677097000	-0.005661000	-3.967766000
H	7.961291000	2.152210000	1.778129000
N	6.275884000	0.199272000	-2.049541000
C	6.554908000	-0.109501000	-4.783918000
H	5.630951000	-2.318283000	-5.859145000
C	7.177838000	1.887523000	2.478991000
H	6.682204000	-0.169350000	-5.857744000
H	8.125988000	2.767643000	4.189323000
C	5.008740000	-1.959508000	-6.683596000
C	5.189777000	0.051644000	-2.832209000
H	4.543437000	-2.836510000	-7.143097000
C	7.258980000	2.227518000	3.823866000
C	5.260486000	-0.109551000	-4.227284000
N	6.114588000	1.217967000	1.986910000
H	5.675130000	-1.529143000	-7.443353000

H	4.230007000	0.069171000	-2.328802000
C	3.944557000	-0.982785000	-6.227377000
C	6.212615000	1.882449000	4.674344000
C	4.029027000	-0.192537000	-5.049141000
C	5.102475000	0.877469000	2.810956000
H	6.145355000	-0.455137000	6.012177000
H	6.249154000	2.163639000	5.721519000
C	5.091654000	1.193111000	4.177066000
H	2.150988000	-2.428466000	-8.341721000
H	4.282088000	0.322760000	2.369425000
C	5.402469000	-0.321721000	6.803141000
H	5.835444000	0.352268000	7.554369000
C	2.787308000	-0.848327000	-6.995708000
C	2.544314000	-1.402426000	-8.383052000
C	2.940623000	0.625750000	-4.687885000
H	3.051869000	1.292705000	-3.835720000
H	5.271356000	-1.290781000	7.294622000
H	3.448381000	-1.426467000	-8.999724000
C	3.927268000	0.849113000	5.031798000
C	4.078857000	0.191819000	6.277808000
C	1.675487000	-0.075293000	-6.593954000
C	1.753024000	0.706577000	-5.420471000
C	1.465225000	-0.433095000	-8.949496000
C	2.657007000	1.289848000	4.621512000
H	2.581946000	1.872680000	3.705352000
H	3.700258000	-0.219129000	9.072225000
C	2.924616000	0.017579000	7.041887000
C	0.674738000	-0.088238000	-7.678746000
H	1.099630000	2.436181000	-4.316304000
C	0.689410000	1.693828000	-5.009613000
H	-1.099656000	-2.437348000	-4.315663000
C	2.846166000	-0.515244000	8.454599000
H	2.220347000	2.149735000	8.904508000
H	2.348840000	1.383630000	10.499543000
C	1.497084000	1.106287000	5.383917000
H	0.291458000	2.224779000	-5.880665000
H	2.806146000	-1.614195000	8.462618000
C	1.637617000	0.403397000	6.600848000
C	-0.689435000	-1.695181000	-5.009170000
C	1.723475000	1.467721000	9.604097000
H	-0.291490000	-2.226366000	-5.880083000
C	-0.674758000	0.086190000	-7.678768000
H	-0.165021000	1.212634000	-4.519517000
H	0.165001000	-1.213863000	-4.519205000
C	1.506428000	0.079935000	8.970363000
H	0.417504000	2.577119000	4.242716000
C	0.210876000	1.764057000	4.946909000
H	0.775625000	1.927550000	9.901545000

C	0.668783000	0.126481000	7.683069000
C	-1.753045000	-0.708033000	-5.420285000
H	-0.321057000	2.184442000	5.806497000
C	-1.465245000	0.430716000	-8.949608000
C	-1.675507000	0.073530000	-6.593973000
H	1.052737000	-0.580966000	9.712484000
H	0.321072000	-2.182898000	5.807079000
H	0.481420000	-1.065506000	4.461838000
C	-0.210866000	-1.762747000	4.947379000
H	-0.481413000	1.066684000	4.461560000
C	-2.940642000	-0.627010000	-4.687718000
C	-2.544331000	1.400197000	-8.383417000
H	-3.051890000	-1.293740000	-3.835378000
H	-3.448398000	1.424081000	-9.000095000
C	-0.668766000	-0.124442000	7.683104000
C	-2.787325000	0.846462000	-6.995928000
H	-0.417497000	-2.576001000	4.243408000
C	-1.497072000	-1.104858000	5.384215000
C	-1.637601000	-0.401645000	6.600959000
H	-1.052714000	0.583547000	9.712331000
C	-1.506407000	-0.077551000	8.970387000
C	-4.029044000	0.191187000	-5.049187000
H	-0.775602000	-1.924918000	9.902062000
H	-2.151002000	2.426248000	-8.342354000
C	-3.944573000	0.981125000	-6.227631000
C	-1.723452000	-1.465168000	9.604493000
C	-2.656996000	-1.288620000	4.621861000
H	-2.581938000	-1.871696000	3.705855000
H	-4.230017000	-0.069795000	-2.328779000
C	-2.924599000	-0.015710000	7.041898000
H	-2.806127000	1.616443000	8.462195000
C	-2.846146000	0.517490000	8.454468000
C	-5.260501000	0.108423000	-4.227305000
H	-2.220326000	-2.147369000	8.905087000
C	-5.189788000	-0.052401000	-2.832188000
C	-5.008753000	1.957732000	-6.684105000
H	-5.675143000	1.527170000	-7.443750000
H	-2.348816000	-1.380838000	10.499918000
C	-3.927257000	-0.847775000	5.032033000
C	-6.554925000	0.108226000	-4.783935000
H	-4.543448000	2.834613000	-7.143834000
C	-4.078842000	-0.190150000	6.277869000
H	-6.682223000	0.167787000	-5.857778000
H	-3.700237000	0.221538000	9.072175000
N	-6.275894000	-0.199816000	-2.049477000
H	-5.630963000	2.316725000	-5.859748000
H	-4.282080000	-0.322127000	2.369519000
C	-5.091643000	-1.192000000	4.177393000

C	-5.102466000	-0.876720000	2.811199000
C	-7.677112000	0.004607000	-3.967753000
C	-7.507805000	-0.148583000	-2.597860000
C	-5.402453000	0.323530000	6.803067000
H	-6.145338000	0.456739000	6.012067000
C	-6.212604000	-1.881207000	4.674855000
H	-6.249141000	-2.162118000	5.722104000
N	-6.114579000	-1.217440000	1.987245000
H	-8.677601000	0.020449000	-4.387057000
H	-5.271338000	1.292719000	7.294293000
C	-7.258968000	-2.226503000	3.824470000
H	-5.835429000	-0.350260000	7.554471000
C	-7.177828000	-1.886866000	2.479504000
H	-8.125976000	-2.766531000	4.190072000
H	-7.961281000	-2.151741000	1.778713000
C	-2.115470000	-0.792107000	-9.624492000
H	-1.363618000	-1.513261000	-9.960458000
H	-2.692328000	-0.480024000	-10.501834000
H	-2.793770000	-1.311467000	-8.937885000
H	-0.831802000	0.942418000	-9.678350000
C	2.115446000	0.789553000	-9.624700000
H	2.692301000	0.477243000	-10.501963000
H	2.793747000	1.309093000	-8.938231000
H	1.363592000	1.510618000	-9.960851000
H	0.831783000	-0.944989000	-9.678103000
Pd	6.133346000	0.686248000	-0.027924000
Pd	-6.133347000	-0.686255000	-0.027732000
H	-8.353318000	-0.238773000	-1.924978000

(*S*,*S*)$_2$(*R*,*R*)$_2$

Sum of electronic and thermal free energies: -5952.784253

C	1.128608000	-4.727036000	4.745265000
C	2.275198000	-5.548696000	4.654025000
C	3.507903000	-5.093888000	4.185941000
C	3.607965000	-3.711147000	3.883215000
C	2.478211000	-2.884260000	4.023306000
C	1.230472000	-3.348441000	4.454812000
H	2.597775000	-1.813522000	3.869398000
C	4.642008000	-6.084272000	4.022546000
H	5.139284000	-6.307851000	4.975924000
H	5.406766000	-5.736549000	3.322947000
H	4.262224000	-7.037147000	3.641362000
C	0.119194000	-2.361837000	4.715103000
H	0.517910000	-1.344560000	4.792240000
H	-0.397621000	-2.596472000	5.651518000
H	-0.644017000	-2.364062000	3.928498000
C	0.039290000	-5.509118000	5.366100000
C	0.744188000	-6.637301000	6.135196000

C	1.969553000	-6.917541000	5.219734000
H	1.698587000	-7.626875000	4.424335000
H	2.810654000	-7.352714000	5.768791000
C	1.192093000	-6.227841000	7.551540000
H	0.107136000	-7.521914000	6.207180000
H	1.871303000	-5.368183000	7.520303000
H	1.715113000	-7.057420000	8.039629000
H	0.338934000	-5.955983000	8.181165000
C	4.901553000	-3.072865000	3.537748000
C	4.993725000	-2.111550000	2.518466000
N	6.139964000	-1.470589000	2.214629000
C	7.268063000	-1.766445000	2.891820000
C	6.086001000	-3.349800000	4.246664000
C	7.273285000	-2.705796000	3.914854000
H	8.169853000	-1.236389000	2.607293000
H	6.068047000	-4.054430000	5.070204000
H	4.123290000	-1.853665000	1.925641000
H	-4.312787000	-5.527133000	6.808379000
C	-1.316682000	-5.410789000	5.307961000
C	-2.230986000	-6.115333000	6.322239000
C	-3.370066000	-5.070245000	6.490839000
H	-0.577084000	-5.687061000	2.455678000
C	-2.229728000	-4.652155000	4.427931000
H	-1.236394000	-4.701068000	1.137031000
C	-3.447290000	-4.445446000	5.115764000
H	-3.096602000	-4.325932000	7.252706000
C	-0.953298000	-4.692274000	2.194875000
H	-6.400056000	-4.450935000	5.526422000
C	-2.135525000	-4.324379000	3.057255000
C	-4.557402000	-3.828461000	4.538950000
C	-5.775874000	-3.556693000	5.396215000
H	-0.111453000	-4.000098000	2.311762000
C	-4.458766000	-3.486891000	3.165487000
H	-5.472461000	-3.242893000	6.399702000
H	-6.410290000	-2.766157000	4.986593000
H	-7.065785000	-4.381250000	3.172906000
C	-5.618638000	-2.977170000	2.393951000
C	-6.901332000	-3.547723000	2.499574000
C	-5.474598000	-1.933091000	1.466784000
C	-7.952473000	-3.068433000	1.724623000
H	-4.517170000	-1.439708000	1.340377000
H	-8.945685000	-3.498742000	1.797052000
N	-6.488937000	-1.486728000	0.699791000
C	-7.714138000	-2.036033000	0.826409000
H	-8.500543000	-1.642121000	0.192948000
C	-2.784438000	-7.461929000	5.817515000
H	-1.981747000	-8.180545000	5.623865000
H	-3.350527000	-7.334947000	4.887595000

H	-3.452611000	-7.902658000	6.565363000
H	-1.714156000	-6.278100000	7.270912000
H	8.193610000	-2.912307000	4.450843000
C	-1.128607000	4.727031000	-4.745267000
C	-0.039290000	5.509120000	-5.366096000
C	1.316683000	5.410798000	-5.307950000
C	2.230988000	6.115350000	-6.322223000
C	3.370074000	5.070269000	-6.490820000
C	3.447295000	4.445466000	-5.115745000
C	4.557408000	3.828485000	-4.538928000
C	5.618636000	2.977189000	-2.393929000
C	5.474597000	1.933103000	-1.466771000
H	4.517171000	1.439713000	-1.340374000
N	6.488934000	1.486739000	-0.699775000
C	7.714133000	2.036052000	-0.826381000
H	8.500536000	1.642142000	-0.192918000
C	7.952467000	3.068461000	-1.724586000
H	8.945677000	3.498776000	-1.797006000
C	6.901328000	3.547750000	-2.499540000
H	7.065781000	4.381282000	-3.172865000
C	2.135521000	4.324388000	-3.057243000
C	5.775885000	3.556727000	-5.396187000
H	5.472480000	3.242931000	-6.399678000
H	6.400065000	4.450973000	-5.526387000
H	6.410302000	2.766192000	-4.986565000
H	4.312794000	5.527163000	-6.808353000
H	3.096619000	4.325956000	-7.252690000
C	2.229729000	4.652168000	-4.427918000
C	-0.744190000	6.637299000	-6.135196000
C	-1.969562000	6.917531000	-5.219742000
H	-1.698605000	7.626868000	-4.424341000
H	-2.810663000	7.352699000	-5.768803000
C	-1.230465000	3.348436000	-4.454814000
C	-2.478203000	2.884249000	-4.023312000
H	-2.597762000	1.813510000	-3.869403000
C	-3.607962000	3.711130000	-3.883227000
C	-4.901549000	3.072841000	-3.537767000
C	-6.085993000	3.349764000	-4.246694000
H	-6.068036000	4.054389000	-5.070238000
C	-7.273275000	2.705755000	-3.914890000
H	-8.193596000	2.912256000	-4.450888000
C	-7.268056000	1.766411000	-2.891850000
H	-8.169844000	1.236350000	-2.607327000
N	-6.139960000	1.470567000	-2.214646000
C	-4.993723000	2.111533000	-2.518478000
H	-4.123291000	1.853659000	-1.925644000
C	-3.507907000	5.093870000	-4.185955000
C	-4.642020000	6.084247000	-4.022569000

H	-5.139290000	6.307823000	-4.975951000
H	-5.406781000	5.736519000	-3.322976000
H	-4.262244000	7.037125000	-3.641382000
C	-2.275202000	5.548685000	-4.654034000
C	-1.192084000	6.227836000	-7.551543000
H	-1.871289000	5.368175000	-7.520310000
H	-0.338919000	5.955983000	-8.181162000
H	-1.715106000	7.057412000	-8.039635000
H	-0.107143000	7.521916000	-6.207176000
C	2.784429000	7.461947000	-5.817493000
H	1.714161000	6.278115000	-7.270898000
H	3.350514000	7.334967000	-4.887571000
H	1.981733000	8.180558000	-5.623846000
H	3.452602000	7.902683000	-6.565338000
C	-0.119180000	2.361837000	-4.715099000
H	0.397639000	2.596477000	-5.651510000
H	-0.517892000	1.344559000	-4.792242000
H	0.644025000	2.364064000	-3.928489000
C	0.953289000	4.692275000	-2.194867000
H	1.236382000	4.701071000	-1.137022000
H	0.111449000	4.000095000	-2.311756000
H	0.577070000	5.687060000	-2.455671000
H	-3.350554000	7.334938000	4.887589000
H	-1.236413000	4.701062000	1.137032000
H	3.096623000	-4.325902000	-7.252718000
H	5.472474000	-3.242852000	-6.399717000
H	6.400081000	-4.450886000	-5.526439000
H	-1.981777000	8.180535000	5.623864000
C	-2.784469000	7.461920000	5.817511000
H	-0.338901000	-5.955968000	-8.181177000
H	-3.452644000	7.902650000	6.565357000
C	5.775892000	-3.556649000	-5.396230000
H	-0.517893000	-1.344553000	-4.792242000
H	-8.945703000	3.498710000	1.797032000
C	-7.952489000	3.068404000	1.724606000
H	-1.871272000	-5.368175000	-7.520314000
C	-1.192059000	-6.227830000	-7.551553000
C	-6.901351000	3.547700000	2.499559000
H	-7.065810000	4.381228000	3.172887000
C	-0.953319000	4.692268000	2.194877000
C	3.370092000	-5.070215000	-6.490853000
C	-7.714148000	2.036001000	0.826397000
H	-1.715076000	-7.057410000	-8.039644000
H	-3.237664000	3.552399000	1.397074000
H	0.397643000	-2.596460000	-5.651520000
C	-3.263136000	3.740986000	2.468572000
H	-8.500551000	1.642085000	0.192935000
H	4.312815000	-5.527097000	-6.808394000

C	-5.618655000	2.977151000	2.393941000
C	-0.119174000	-2.361828000	-4.715105000
H	-0.577107000	5.687055000	2.455682000
C	-2.135547000	4.324370000	3.057253000
C	-4.458787000	3.486877000	3.165480000
N	-6.488945000	1.486700000	0.699785000
H	-6.400089000	4.450922000	5.526403000
H	1.714188000	-6.278078000	-7.270926000
H	6.410302000	-2.766108000	-4.986609000
C	-5.474610000	1.933069000	1.466779000
H	1.698567000	7.626863000	4.424357000
C	4.557424000	-3.828427000	-4.538963000
C	3.447315000	-4.445418000	-5.115776000
C	-7.273264000	-2.705807000	-3.914877000
C	-2.229755000	4.652146000	4.427929000
C	-4.557429000	3.828449000	4.538941000
C	-7.268048000	-1.766461000	-2.891838000
C	2.231019000	-6.115310000	-6.322253000
H	-8.193585000	-2.912317000	-4.450871000
H	-0.111473000	4.000094000	2.311765000
H	-8.169840000	-1.236407000	-2.607314000
C	-2.231020000	6.115324000	6.322238000
C	-3.447320000	4.445436000	5.115758000
C	-6.085977000	-3.349808000	-4.246683000
H	0.644035000	-2.364052000	-3.928499000
H	-4.312823000	5.527124000	6.808369000
C	-5.775903000	3.556681000	5.396202000
H	4.262211000	7.037130000	3.641399000
N	-6.139953000	-1.470606000	-2.214640000
C	-1.316712000	5.410780000	5.307963000
H	-4.517180000	1.439688000	1.340376000
C	-1.230449000	-3.348436000	-4.454818000
C	6.901356000	-3.547672000	-2.499594000
C	7.952495000	-3.068375000	-1.724645000
C	-0.744153000	-6.637291000	-6.135209000
C	1.316712000	-5.410773000	-5.307972000
H	-6.068017000	-4.054434000	-5.070226000
H	7.065816000	-4.381195000	-3.172929000
C	-4.901533000	-3.072874000	-3.537760000
C	-0.039260000	-5.509108000	-5.366111000
H	8.945712000	-3.498674000	-1.797079000
C	-3.370100000	5.070236000	6.490834000
C	-4.993712000	-2.111564000	-2.518474000
H	0.107105000	7.521902000	6.207192000
C	1.969526000	6.917526000	5.219757000
C	4.458788000	-3.486861000	-3.165499000
C	5.618657000	-2.977132000	-2.393965000
C	7.714154000	-2.035980000	-0.826426000

C	2.229755000	-4.652137000	-4.427942000
C	-1.128581000	-4.727031000	-4.745274000
C	0.039260000	5.509107000	5.366108000
C	5.474610000	-1.933059000	-1.466793000
C	0.744155000	6.637287000	6.135210000
H	-1.714193000	6.278091000	7.270913000
N	6.488947000	-1.486688000	-0.699802000
H	8.500557000	-1.642063000	-0.192967000
H	-0.107099000	-7.521902000	-6.207195000
H	-5.472494000	3.242886000	6.399692000
H	-4.123280000	-1.853680000	-1.925644000
C	4.641989000	6.084253000	4.022586000
C	2.275172000	5.548682000	4.654047000
H	3.452652000	-7.902628000	-6.565382000
C	2.784478000	-7.461903000	-5.817532000
H	2.810625000	7.352697000	5.768819000
H	-6.410315000	2.766142000	4.986580000
C	-2.275168000	-5.548695000	-4.654038000
C	1.128580000	4.727024000	4.745277000
C	3.507879000	5.093873000	4.185968000
H	4.517178000	-1.439685000	-1.340381000
C	-3.607942000	-3.711151000	-3.883224000
C	3.263141000	-3.740978000	-2.468587000
C	-1.969518000	-6.917537000	-5.219749000
C	2.135552000	-4.324365000	-3.057265000
H	-2.810618000	-7.352712000	-5.768807000
H	5.406753000	5.736529000	3.322995000
C	-3.507875000	-5.093891000	-4.185953000
C	3.607939000	3.711133000	3.883238000
H	-3.096640000	4.325924000	7.252702000
C	1.230443000	3.348430000	4.454821000
H	5.139256000	6.307830000	4.975969000
H	-5.139251000	-6.307858000	-4.975943000
H	1.981792000	-8.180524000	-5.623882000
H	3.350568000	-7.334920000	-4.887613000
H	-0.644043000	2.364052000	3.928494000
H	6.068008000	4.054394000	5.070259000
C	1.192050000	6.227825000	7.551557000
C	0.119162000	2.361826000	4.715104000
H	3.237673000	-3.552395000	-1.397088000
C	-4.641978000	-6.084278000	-4.022564000
H	-0.397659000	2.596462000	5.651516000
C	6.085969000	3.349772000	4.246712000
C	0.953329000	-4.692270000	-2.194884000
H	0.111480000	-4.000099000	-2.311766000
H	1.715069000	7.057402000	8.039650000
C	4.901529000	3.072850000	3.537779000
H	-1.698551000	-7.626872000	-4.424351000

H	0.338887000	5.955967000	8.181175000
H	0.577121000	-5.687058000	-2.455689000
H	-5.406739000	-5.736558000	-3.322966000
C	7.273254000	2.705766000	3.914910000
H	8.193573000	2.912266000	4.450913000
H	1.871258000	5.368166000	7.520322000
C	4.993709000	2.111547000	2.518486000
H	-4.262192000	-7.037153000	-3.641379000
C	7.268041000	1.766427000	2.891866000
H	0.517877000	1.344550000	4.792245000
H	1.236428000	-4.701065000	-1.137040000
N	6.139949000	1.470584000	2.214656000
H	4.123280000	1.853674000	1.925647000
H	8.169831000	1.236368000	2.607345000
Pd	-6.248387000	-0.000017000	-0.737052000
Pd	6.248386000	0.000011000	0.737051000
C	-2.478190000	-2.884261000	-4.023311000
H	-2.597758000	-1.813524000	-3.869401000
C	2.478183000	2.884248000	4.023319000
H	2.597746000	1.813510000	3.869408000
C	4.458766000	3.486910000	-3.165468000
C	3.263112000	3.741009000	-2.468562000
H	3.237640000	3.552419000	-1.397064000
C	-3.263116000	-3.740998000	2.468577000
H	-3.237648000	-3.552412000	1.397078000

(*S,S*)(*R,R*)(*S,S*)(*R,R*)
Sum of electronic and thermal free energies: -5952.78232

C	-1.685040000	-3.237044000	5.739404000
C	-2.810639000	-2.809066000	6.479404000
C	-3.978942000	-2.329333000	5.887304000
C	-4.045567000	-2.376141000	4.470469000
C	-2.942749000	-2.852173000	3.737610000
C	-1.754350000	-3.293876000	4.329815000
H	-3.045330000	-2.961465000	2.659668000
C	-5.081404000	-1.788123000	6.773578000
H	-5.693703000	-2.587929000	7.211322000
H	-5.753372000	-1.109133000	6.241910000
H	-4.654426000	-1.231353000	7.613311000
C	-0.683484000	-3.920425000	3.471603000
H	-1.090607000	-4.206078000	2.495700000
H	-0.279714000	-4.819796000	3.948158000
H	0.166744000	-3.251100000	3.297165000
C	-0.676126000	-3.763302000	6.681961000
C	-1.465330000	-4.136705000	7.946334000
C	-2.571809000	-3.043868000	7.954305000
H	-2.205907000	-2.132833000	8.449656000
H	-3.471587000	-3.364142000	8.489207000

C	-2.080446000	-5.548416000	7.891692000
H	-0.839395000	-4.057404000	8.838262000
H	-2.752869000	-5.655679000	7.032830000
H	-2.656356000	-5.747701000	8.801958000
H	-1.309360000	-6.321063000	7.810077000
C	-5.287956000	-2.049675000	3.728881000
C	-5.253003000	-1.312557000	2.534274000
N	-6.353731000	-1.042547000	1.804841000
C	-7.559195000	-1.468143000	2.234359000
C	-6.552955000	-2.506703000	4.145105000
C	-7.690981000	-2.203903000	3.404963000
H	-8.419319000	-1.215403000	1.625146000
H	-6.638461000	-3.115855000	5.037581000
H	-4.313891000	-0.924235000	2.156000000
H	3.472442000	-5.652565000	7.171281000
C	0.676811000	-3.889421000	6.609259000
C	1.466146000	-4.795332000	7.566954000
C	2.572616000	-5.350831000	6.625785000
H	0.280035000	-0.994791000	6.150186000
C	1.685613000	-3.338441000	5.680810000
H	1.090742000	-0.046803000	4.890065000
C	2.811299000	-4.193179000	5.682185000
H	2.206748000	-6.236655000	6.086619000
C	0.683758000	-1.034239000	5.133106000
H	5.694293000	-4.936966000	5.858505000
C	1.754745000	-2.090893000	5.022161000
C	3.979550000	-3.921868000	4.969983000
C	5.082143000	-4.959924000	4.946932000
H	-0.166480000	-1.219600000	4.466738000
C	4.046003000	-2.673073000	4.299054000
H	4.655301000	-5.965735000	4.886846000
H	5.754232000	-4.840850000	4.092910000
H	6.638841000	-2.791596000	5.223641000
C	5.288334000	-2.195460000	3.644393000
C	6.553316000	-2.325648000	4.248720000
C	5.253318000	-1.532528000	2.407082000
C	7.691269000	-1.837325000	3.615323000
H	4.314218000	-1.400633000	1.881239000
H	8.672326000	-1.936034000	4.067745000
N	6.353979000	-1.037010000	1.807412000
C	7.559419000	-1.194425000	2.391257000
H	8.419484000	-0.794188000	1.866920000
C	2.081265000	-4.039237000	8.760362000
H	1.310178000	-3.580720000	9.387582000
H	2.753663000	-3.242617000	8.421873000
H	2.657201000	-4.726365000	9.389746000
H	0.840290000	-5.606550000	7.946224000
H	-8.672049000	-2.545398000	3.717674000

C	1.684974000	5.739472000	3.236835000
C	0.676042000	6.682061000	3.763004000
C	-0.676897000	6.609364000	3.889099000
C	-1.466253000	7.567131000	4.794917000
C	-2.572732000	6.626009000	5.350473000
C	-2.811386000	5.682303000	4.192901000
C	-3.979626000	4.970068000	3.921631000
C	-5.288360000	3.644285000	2.195333000
C	-5.253329000	2.406854000	1.532630000
H	-4.314231000	1.880959000	1.400919000
N	-6.353966000	1.807123000	1.037129000
C	-7.559398000	2.391054000	1.194325000
H	-8.419445000	1.866687000	0.794091000
C	-7.691265000	3.615226000	1.837010000
H	-8.672316000	4.067695000	1.935558000
C	-6.553332000	4.248669000	2.325331000
H	-6.638866000	5.223664000	2.791124000
C	-1.754795000	5.022106000	2.090688000
C	-5.082256000	4.947115000	4.959652000
H	-4.655451000	4.887150000	5.965485000
H	-5.694424000	5.858672000	4.936568000
H	-5.754325000	4.093067000	4.840645000
H	-3.472567000	7.171530000	5.652135000
H	-2.206886000	6.086924000	6.236356000
C	-1.685685000	5.680863000	3.338181000
C	1.465231000	7.946464000	4.136338000
C	2.571742000	7.954363000	3.043534000
H	2.205866000	8.449655000	2.132455000
H	3.471509000	8.489288000	3.363800000
C	1.754287000	4.329888000	3.293762000
C	2.942686000	3.737656000	2.852095000
H	3.045267000	2.659721000	2.961449000
C	4.045509000	4.470486000	2.376034000
C	5.287900000	3.728876000	2.049621000
C	6.552876000	4.145067000	2.506732000
H	6.638355000	5.037530000	3.115909000
C	7.690905000	3.404893000	2.204020000
H	8.671951000	3.717561000	2.545615000
C	7.559136000	2.234307000	1.468222000
H	8.419261000	1.625060000	1.215570000
N	6.353701000	1.804840000	1.042516000
C	5.252968000	2.534285000	1.312470000
H	4.313860000	2.156013000	0.924136000
C	3.978894000	5.887320000	2.329157000
C	5.081379000	6.773560000	1.787942000
H	5.693640000	7.211344000	2.587755000
H	5.753381000	6.241851000	1.109017000
H	4.654431000	7.613262000	1.231104000

C	2.810580000	6.479447000	2.808830000
C	2.080306000	7.891923000	5.548071000
H	2.752728000	7.033071000	5.655413000
H	1.309199000	7.810359000	6.320702000
H	2.656208000	8.802204000	5.747309000
H	0.839296000	8.838385000	4.056957000
C	-2.081361000	8.760473000	4.038707000
H	-0.840414000	7.946472000	5.606115000
H	-2.753745000	8.421911000	3.242106000
H	-1.310268000	9.387653000	3.580146000
H	-2.657310000	9.389916000	4.725769000
C	0.683428000	3.471716000	3.920379000
H	0.279678000	3.948325000	4.819730000
H	1.090554000	2.495828000	4.206082000
H	-0.166814000	3.297242000	3.251083000
C	-0.683810000	5.132991000	1.034027000
H	-1.090786000	4.889850000	0.046614000
H	0.166450000	4.466668000	1.219446000
H	-0.280121000	6.150081000	0.994490000
H	2.753620000	-7.032189000	-5.656047000
H	1.090780000	-2.495415000	-4.205936000
H	-2.206553000	2.132533000	-8.449499000
H	-4.654919000	1.230720000	-7.613129000
H	-5.694379000	2.587139000	-7.211087000
H	1.310158000	-7.809642000	-6.321290000
C	2.081355000	-7.891169000	-5.548745000
H	1.309445000	3.580888000	-9.387413000
H	2.657393000	-8.801328000	-5.748152000
C	-5.081960000	1.787408000	-6.773374000
H	1.090540000	0.046796000	-4.890050000
H	8.672573000	-3.716069000	-2.545922000
C	7.691473000	-3.403612000	-2.204289000
H	2.752965000	3.242989000	-8.421686000
C	2.080450000	4.039512000	-8.760171000
C	6.553565000	-4.143912000	-2.507143000
H	6.639189000	-5.036251000	-3.116480000
C	0.683861000	-3.471416000	-3.920327000
C	-2.572552000	3.043517000	-7.954123000
C	7.559524000	-2.233184000	-1.468274000
H	2.656291000	4.726741000	-9.389532000
H	3.045647000	-2.659124000	-2.961480000
H	0.279693000	0.994687000	-6.150151000
C	2.943264000	-3.737093000	-2.852271000
H	8.419555000	-1.623870000	-1.215472000
H	-3.472377000	3.363697000	-8.489003000
C	5.288525000	-3.728021000	-2.049932000
C	0.683389000	1.034169000	-5.133061000
H	0.280113000	-3.947978000	-4.819705000

C	1.754933000	-4.329476000	-3.293924000
C	4.046253000	-4.469801000	-2.376399000
N	6.354011000	-1.803981000	-1.042496000
H	5.694859000	-7.210338000	-2.588621000
H	-0.840268000	4.057266000	-8.838090000
H	-5.753822000	1.108308000	-6.241712000
C	5.253399000	-2.533565000	-1.312573000
H	-2.206041000	-6.086798000	-6.236542000
C	-3.979559000	2.328753000	-5.887109000
C	-2.811333000	2.808655000	-6.479222000
C	7.690745000	1.838193000	-3.615353000
C	1.685872000	-5.739078000	-3.237186000
C	3.979890000	-5.886654000	-2.329720000
C	7.559029000	1.195287000	-2.391276000
C	-1.466198000	4.136482000	-7.946151000
H	8.671762000	1.937009000	-4.067836000
H	-0.166351000	-3.297188000	-3.250928000
H	8.419173000	0.795158000	-1.866989000
C	1.466425000	-7.945991000	-4.136962000
C	2.811643000	-6.478917000	-2.809384000
C	6.552698000	2.326386000	-4.248686000
H	-0.166895000	1.219355000	-4.466704000
H	3.472870000	-8.488567000	-3.364681000
C	5.082569000	-6.772775000	-1.788708000
H	-4.654794000	-4.887447000	-5.965467000
N	6.353642000	1.037743000	-1.807354000
C	0.677057000	-6.681767000	-3.763397000
H	4.314252000	-2.155563000	-0.924075000
C	1.754193000	2.091002000	-5.022058000
C	-6.553569000	2.505682000	-4.144822000
C	-7.691517000	2.202684000	-3.404639000
C	1.465199000	4.795487000	-7.566753000
C	-0.676934000	3.763149000	-6.681793000
H	6.638113000	2.792346000	-5.223612000
H	-6.639209000	3.114837000	-5.037282000
C	5.287768000	2.196052000	-3.644291000
C	0.675987000	3.889435000	-6.609093000
H	-8.672654000	2.544016000	-3.717308000
C	2.573039000	-7.953842000	-3.044262000
C	5.252894000	1.533131000	-2.406969000
H	-0.839288000	-7.946225000	-5.606545000
C	-2.571783000	-6.626035000	-5.350709000
C	-4.046162000	2.375528000	-4.470269000
C	-5.288479000	2.048860000	-3.728649000
C	-7.559565000	1.466936000	-2.234046000
C	-1.685774000	3.236758000	-5.739234000
C	1.684866000	3.338567000	-5.680659000
C	-0.675902000	-6.609266000	-3.889401000

C	-5.253361000	1.311740000	-2.534046000
C	-1.465152000	-7.567054000	-4.795287000
H	0.840650000	-8.838028000	-4.057634000
N	-6.354016000	1.041538000	-1.804573000
H	-8.419629000	1.214050000	-1.624807000
H	0.839227000	5.606622000	-7.946011000
H	4.655806000	-7.612636000	-1.231967000
H	4.313829000	1.401122000	-1.881090000
C	-5.081551000	-4.947585000	-4.959624000
C	-2.810537000	-5.682492000	-4.193027000
H	-2.657416000	5.747359000	-8.801729000
C	-2.081468000	5.548123000	-7.891477000
H	-3.471547000	-7.171654000	-5.652406000
H	5.754515000	-6.241047000	-1.109743000
C	2.810423000	4.193476000	-5.681994000
C	-1.684810000	-5.680978000	-3.338339000
C	-3.978878000	-4.970464000	-3.921646000
H	-4.314171000	0.923568000	-2.155812000
C	4.045356000	2.673505000	-4.298917000
C	-2.943397000	2.851707000	-3.737424000
C	2.571576000	5.351124000	-6.625557000
C	-1.755069000	3.293575000	-4.329647000
H	3.471360000	5.653011000	-7.171036000
H	-5.753756000	-4.093660000	-4.840503000
C	3.978716000	3.922311000	-4.969804000
C	-4.045358000	-4.299538000	-2.672854000
H	2.207333000	-8.449316000	-2.133214000
C	-1.753985000	-5.022358000	-2.090775000
H	-5.693570000	-5.859243000	-4.936608000
H	5.693308000	4.937686000	-5.858304000
H	-1.310462000	6.320850000	-7.809867000
H	-2.753885000	5.655303000	-7.032601000
H	0.167188000	-4.466721000	-1.219494000
H	-6.638014000	-5.224639000	-2.791013000
C	-2.080070000	-8.760556000	-4.039175000
C	-0.682966000	-5.133198000	-1.034142000
H	-3.045976000	2.960984000	-2.659481000
C	5.081167000	4.960521000	-4.946723000
H	-0.279118000	-6.150229000	-0.994725000
C	-6.552635000	-4.249651000	-2.325177000
C	-0.684267000	3.920267000	-3.471460000
H	0.166070000	3.251069000	-3.297069000
H	-2.655951000	-9.390013000	-4.726282000
C	-5.287764000	-3.645054000	-2.195193000
H	2.205571000	6.236876000	-6.086366000
H	-1.308879000	-9.387675000	-3.580696000
H	-0.280653000	4.819710000	-3.948011000
H	5.753285000	4.841497000	-4.092717000

C	-7.690663000	-3.616429000	-1.836793000
H	-8.671637000	-4.069066000	-1.935326000
H	-2.752479000	-8.422165000	-3.242522000
C	-5.252923000	-2.407649000	-1.532434000
H	4.654188000	5.966270000	-4.886586000
C	-7.558987000	-2.392258000	-1.194063000
H	-1.089966000	-4.890234000	-0.046693000
H	-1.091399000	4.205836000	-2.495537000
N	-6.353651000	-1.808128000	-1.036880000
H	-4.313910000	-1.881606000	-1.400711000
H	-8.419111000	-1.868052000	-0.793783000
Pd	6.284063000	0.000383000	0.000055000
Pd	-6.284069000	-0.000499000	0.000131000
C	2.942569000	1.800675000	-4.342665000
H	3.045165000	0.813598000	-3.895920000
C	-2.942395000	-4.343110000	-1.800239000
H	-3.044858000	-3.896356000	-0.813153000
C	-4.046044000	4.299008000	2.672906000
C	-2.943114000	4.342656000	1.800253000
H	-3.045538000	3.895789000	0.813213000
C	2.943083000	-1.800407000	4.342767000
H	3.045530000	-0.813324000	3.896004000

Pd_2(stable *E*-1)$_4$

(*S,S*)$_4$

Sum of electronic and thermal free energies: -5952.759457

C	-3.650872000	2.602609000	-3.600718000
C	-3.939247000	3.067049000	-2.316882000
C	-3.679280000	4.392543000	-1.899181000
C	-3.268554000	5.352548000	-2.843079000
C	-2.974192000	4.885740000	-4.131670000
C	-3.105038000	3.540563000	-4.513022000
H	-2.617890000	5.604260000	-4.866651000
C	-3.987160000	1.173177000	-3.970594000
H	-4.979470000	0.902861000	-3.594954000
H	-3.284922000	0.451154000	-3.530314000
H	-4.000142000	1.011979000	-5.051324000
C	-3.210693000	6.836392000	-2.566140000
H	-3.541730000	7.400016000	-3.444350000
H	-2.199087000	7.183204000	-2.325306000
H	-3.861258000	7.105875000	-1.730715000
C	-4.564001000	2.280587000	-1.184415000
C	-3.971606000	4.466921000	-0.437367000
C	-2.652875000	3.154339000	-5.874722000
C	-3.008929000	3.863774000	-7.034989000

C	-2.506904000	3.472903000	-8.275241000
C	-1.654666000	2.376167000	-8.353288000
N	-1.301764000	1.689099000	-7.247545000
C	-1.783420000	2.069319000	-6.048978000
H	-1.453885000	1.493115000	-5.192315000
H	-3.688139000	4.707824000	-6.963423000
H	-2.777086000	4.004544000	-9.181547000
H	-1.244816000	2.037655000	-9.298185000
C	-3.196582000	5.042843000	0.518849000
C	-1.869222000	5.760272000	0.272205000
C	-0.925559000	5.002029000	1.246772000
C	-1.844322000	4.605685000	2.382599000
H	-0.083066000	5.622260000	1.573730000
H	-0.499817000	4.111354000	0.760309000
C	-3.193545000	4.725358000	1.977302000
C	-1.910062000	7.258068000	0.635205000
H	-1.540332000	5.637319000	-0.762507000
H	-2.149415000	7.395673000	1.695383000
H	-2.659176000	7.801933000	0.055247000
H	-0.935130000	7.723173000	0.449068000
C	-4.221723000	4.584573000	2.928240000
C	-3.843440000	4.158999000	4.209181000
C	-2.511159000	3.910219000	4.576866000
C	-1.466702000	4.186593000	3.658869000
H	-4.626219000	4.006317000	4.949022000
C	-5.666333000	4.940579000	2.667090000
H	-5.753061000	5.649367000	1.840112000
H	-6.109727000	5.404310000	3.554143000
H	-6.280341000	4.066293000	2.421643000
C	-5.024883000	3.385847000	-0.193858000
H	-3.821110000	1.620715000	-0.711248000
H	-5.395002000	1.645119000	-1.511526000
C	-6.456405000	3.841574000	-0.540104000
H	-4.988772000	3.024511000	0.836699000
H	-7.170551000	3.030456000	-0.356950000
H	-6.531470000	4.121165000	-1.596698000
H	-6.768047000	4.705102000	0.051939000
C	0.003066000	4.112226000	4.015829000
H	0.170526000	4.068506000	5.094798000
H	0.533138000	4.995794000	3.645439000
H	0.500073000	3.243188000	3.561895000
C	-2.253204000	3.354374000	5.930506000
C	-1.448192000	2.217958000	6.085767000
N	-1.205482000	1.637197000	7.276116000
C	-1.759669000	2.153539000	8.392152000
C	-2.579890000	3.275580000	8.333079000
C	-2.827578000	3.879307000	7.101333000
H	-3.452495000	4.765378000	7.044565000

H	-0.992090000	1.751883000	5.220193000
H	-1.539358000	1.656054000	9.329899000
H	-3.009093000	3.671786000	9.247320000
H	3.010432000	-3.828735000	9.183005000
C	2.581097000	-3.417017000	8.275708000
H	1.540711000	-1.814705000	9.300118000
H	3.453514000	-4.884651000	6.961880000
C	1.760885000	-2.296147000	8.354001000
C	2.828605000	-3.999676000	7.033822000
H	-0.169788000	-4.154676000	5.024790000
C	-0.002487000	-4.180026000	3.945209000
H	-0.499563000	-3.303390000	3.506205000
N	1.206534000	-1.760872000	7.247002000
H	-0.532607000	-5.057165000	3.559912000
C	2.254061000	-3.454880000	5.872189000
C	1.449072000	-2.321276000	6.046899000
C	1.467230000	-4.248295000	3.586821000
C	2.511820000	-3.987589000	4.509240000
H	0.083294000	-5.648249000	1.477736000
H	0.935196000	-7.729701000	0.317339000
C	0.925738000	-5.022536000	1.161265000
C	1.844665000	-4.645590000	2.303545000
H	0.992845000	-1.840527000	5.189454000
H	2.149663000	-7.423467000	1.568872000
C	1.910156000	-7.267830000	0.511227000
C	3.844048000	-4.230064000	4.137178000
H	0.499924000	-4.123708000	0.690102000
H	4.626935000	-4.089999000	4.879397000
C	1.869261000	-5.764069000	0.173792000
C	3.193829000	-4.758336000	1.896072000
H	2.659185000	-7.801738000	-0.078018000
C	4.222146000	-4.633759000	2.849120000
H	1.540222000	-5.623516000	-0.858630000
C	3.196656000	-5.050940000	0.432424000
H	6.110246000	-5.464024000	3.460696000
C	5.666720000	-4.985257000	2.581734000
H	2.198711000	-7.142541000	-2.447611000
H	3.860968000	-7.075359000	-1.852031000
C	3.971540000	-4.458816000	-0.513958000
C	3.210286000	-6.791686000	-2.682653000
H	5.753329000	-5.679858000	1.742793000
H	6.280687000	-4.106914000	2.351120000
H	4.988904000	-3.038314000	0.784337000
C	3.678997000	-4.359553000	-1.974249000
C	3.268120000	-5.303343000	-2.934296000
H	3.821013000	-1.608358000	-0.739305000
C	5.024859000	-3.382046000	-0.252230000
H	3.541191000	-7.340279000	-3.570379000

C	4.563831000	-2.260077000	-1.223753000
C	2.973572000	-4.814660000	-4.214707000
C	3.938909000	-3.027139000	-2.369356000
H	2.617153000	-5.520557000	-4.961764000
C	6.456326000	-3.831812000	-0.606404000
H	6.768054000	-4.705298000	-0.029203000
C	3.104371000	-3.463183000	-4.573118000
C	3.650345000	-2.540902000	-3.645054000
H	5.394786000	-1.619130000	-1.540116000
H	3.284422000	-0.390958000	-3.537961000
H	1.453125000	-1.404462000	-5.217202000
C	2.652009000	-3.053826000	-5.927976000
C	1.782533000	-1.965994000	-6.083602000
H	6.531230000	-4.093366000	-1.667619000
C	3.986584000	-1.105379000	-3.990585000
H	7.170504000	-3.023931000	-0.409571000
C	3.007892000	-3.743400000	-7.100208000
N	1.300703000	-1.565418000	-7.275450000
H	3.687109000	-4.588546000	-7.043126000
C	1.653440000	-2.233557000	-8.392784000
C	2.505687000	-3.331463000	-8.333550000
H	3.999399000	-0.925799000	-5.068415000
H	4.978956000	-0.841506000	-3.610551000
H	1.243451000	-1.879003000	-9.331719000
H	2.775733000	-3.847593000	-9.248819000
H	3.751062000	2.931185000	9.240527000
H	-1.734763000	-1.619303000	9.302027000
H	1.736116000	1.460657000	9.328001000
H	-3.749723000	-3.088132000	9.189817000
C	3.347021000	2.509687000	8.326131000
C	-2.224314000	-1.831517000	8.358256000
C	2.225529000	1.688914000	8.387910000
C	-3.345815000	-2.651123000	8.282671000
C	3.940146000	2.767809000	7.091381000
N	-1.698570000	-1.267802000	7.251418000
H	4.825697000	3.393248000	7.032285000
N	1.699624000	1.144128000	7.271709000
C	-3.939120000	-2.888181000	7.043791000
H	-4.824679000	-3.512526000	6.974181000
C	3.405181000	2.203300000	5.920298000
C	-2.269262000	-1.500242000	6.054170000
H	4.048661000	4.584566000	4.953317000
C	2.270142000	1.396923000	6.078510000
C	-3.404324000	-2.303811000	5.882414000
C	4.194989000	3.808072000	4.205612000
H	5.434613000	6.079906000	3.559055000
H	-1.795896000	-1.036758000	5.196508000
H	4.086919000	6.259964000	2.439563000

C	3.949380000	2.472716000	4.564161000
H	-4.111398000	0.127052000	5.061553000
H	1.796651000	0.948110000	5.213149000
C	4.963285000	5.643976000	2.672305000
H	-3.273188000	0.469706000	3.538550000
C	-4.146016000	-0.031132000	3.980855000
C	-3.948720000	-2.550095000	4.521964000
C	4.609544000	4.197185000	2.924302000
C	-4.217346000	-1.497776000	3.610670000
H	-4.047931000	-4.668268000	4.875114000
H	4.112115000	-0.213232000	5.058058000
C	4.217862000	1.436066000	3.635040000
H	5.664946000	5.737736000	1.840034000
C	-4.194373000	-3.879155000	4.140762000
H	-5.026438000	0.502151000	3.607595000
C	4.146566000	-0.036669000	3.980205000
H	3.031586000	4.981759000	0.852788000
C	4.742160000	3.177080000	1.963544000
H	3.027290000	7.173537000	-0.322408000
H	4.705384000	6.767708000	0.068672000
C	4.625977000	1.824477000	2.358451000
C	3.836802000	6.461017000	-0.518542000
C	-4.625644000	-1.864395000	2.327711000
C	3.384058000	5.026593000	-0.180492000
H	5.026918000	-0.563531000	3.597778000
H	3.273658000	-0.529889000	3.529575000
C	-4.609111000	-4.246395000	2.853072000
H	-5.434086000	-6.139657000	3.455788000
H	-5.635373000	-0.096195000	1.525446000
C	5.047109000	3.192455000	0.502497000
C	-4.741875000	-3.210079000	1.909845000
H	7.409962000	2.135565000	1.649889000
C	-5.012393000	-0.935886000	1.196511000
H	-7.409726000	-2.163402000	1.614366000
C	-4.962880000	-5.688688000	2.576525000
H	1.632080000	5.407705000	-1.479994000
C	2.270329000	4.574014000	-1.165378000
H	4.107309000	6.545018000	-1.576828000
C	4.462996000	3.975482000	-0.442160000
C	5.012551000	0.915364000	1.211545000
H	1.614541000	3.827112000	-0.692850000
H	-4.086544000	-6.300618000	2.333210000
H	-4.117648000	-0.506035000	0.721261000
C	-7.263177000	-1.914961000	0.557485000
C	7.263251000	1.905160000	0.588951000
H	-7.726704000	-0.938436000	0.375808000
C	5.762393000	1.867276000	0.238502000
H	4.117731000	0.493677000	0.729174000

H	5.635570000	0.070187000	1.526039000
C	-5.762370000	-1.871097000	0.207509000
H	7.802114000	2.659180000	0.010685000
H	-5.664655000	-5.768268000	1.742874000
C	-5.047036000	-3.200572000	0.448791000
C	4.376065000	3.695500000	-1.905697000
C	3.047035000	3.958895000	-2.309791000
H	7.726740000	0.931865000	0.390602000
H	-7.802118000	-2.659032000	-0.033459000
H	-3.031444000	-4.995558000	0.768265000
H	0.871960000	5.009727000	-3.560404000
H	7.090741000	3.876208000	-1.758991000
H	5.630554000	1.547124000	-0.797857000
H	-5.630688000	-1.533340000	-0.823267000
C	-4.463054000	-3.967389000	-0.509149000
C	-3.384067000	-5.022792000	-0.265578000
C	1.139032000	4.020642000	-3.946716000
C	2.571585000	3.681336000	-3.591980000
C	5.327925000	3.292815000	-2.861236000
C	6.814092000	3.232713000	-2.597544000
H	0.968561000	4.042738000	-5.025874000
H	-1.614636000	-3.814736000	-0.757690000
H	-4.705339000	-6.767915000	-0.045911000
H	7.370159000	3.571203000	-3.477728000
C	-2.270486000	-4.553491000	-1.242775000
C	-3.836845000	-6.451256000	-0.627943000
H	-7.090995000	-3.845745000	-1.823727000
H	0.420811000	3.314666000	-3.506195000
H	7.162952000	2.219130000	-2.368258000
H	-3.027297000	-7.167003000	-0.444091000
C	-4.376337000	-3.662521000	-1.967718000
C	4.850070000	3.009311000	-4.148209000
C	3.501668000	3.143283000	-4.516878000
H	-1.632274000	-5.381695000	-1.571636000
C	-6.814471000	-3.188046000	-2.651228000
H	-7.163302000	-2.178522000	-2.404645000
C	-3.047365000	-3.918982000	-2.376432000
C	-5.328341000	-3.243633000	-2.916121000
H	-4.107506000	-6.517224000	-1.687466000
H	4.647845000	3.747133000	-6.972200000
H	5.562247000	2.659269000	-4.892313000
C	3.103763000	2.702604000	-5.878981000
H	-7.370663000	-3.511485000	-3.536974000
C	3.803210000	3.068492000	-7.042249000
H	-0.421322000	-3.254438000	-3.562071000
C	-2.572104000	-3.619621000	-3.653778000
C	-4.850676000	-2.938247000	-4.198150000
C	-1.139600000	-3.952815000	-4.014451000

C	-3.502327000	-3.065907000	-4.569244000
H	-0.872461000	-4.948336000	-3.645085000
C	2.017285000	1.834575000	-6.051249000
H	-5.562966000	-2.575592000	-4.936081000
C	3.401703000	2.576929000	-8.283293000
H	1.448458000	1.497777000	-5.192482000
H	3.925543000	2.854790000	-9.191817000
H	-1.449218000	-1.409127000	-5.217026000
H	-0.969284000	-3.956520000	-5.093853000
C	-3.104623000	-2.602091000	-5.923702000
C	-2.018171000	-1.731251000	-6.081321000
N	1.626787000	1.363028000	-7.250531000
C	2.304336000	1.725301000	-8.359113000
C	-3.804240000	-2.948118000	-7.092931000
H	-4.648864000	-3.627856000	-7.034327000
N	-1.627848000	-1.239346000	-7.272455000
H	1.957723000	1.323418000	-9.304495000
C	-3.402916000	-2.435488000	-8.325481000
C	-2.305561000	-1.582690000	-8.386947000
H	-3.926888000	-2.697838000	-9.238529000
H	-1.959086000	-1.164763000	-9.325398000
Pd	-0.000535000	0.062302000	-7.315727000
Pd	0.000531000	-0.062300000	7.315811000

(*S*,*S*)$_3$(*R*,*R*)

Sum of electronic and thermal free energies: -5952.736258

C	4.139081000	-4.757915000	-0.953241000
C	2.902383000	-5.147512000	-1.471078000
C	2.564153000	-5.030221000	-2.838088000
C	3.567078000	-4.691583000	-3.768603000
C	4.797464000	-4.265761000	-3.246452000
C	5.077532000	-4.229304000	-1.871189000
H	5.567922000	-3.943992000	-3.944287000
C	4.450299000	-5.011344000	0.506121000
H	3.655685000	-4.649008000	1.167339000
H	5.391527000	-4.556589000	0.824696000
H	4.541582000	-6.089928000	0.689108000
C	3.420946000	-4.845121000	-5.265124000
H	4.348889000	-5.236480000	-5.694848000
H	3.205840000	-3.898933000	-5.775195000
H	2.619757000	-5.545372000	-5.511466000
C	1.747304000	-5.732457000	-0.690759000
C	1.106136000	-5.340766000	-2.964666000
C	6.352112000	-3.595697000	-1.430413000
C	7.620980000	-4.016505000	-1.858258000
C	8.759734000	-3.326587000	-1.441069000
C	8.622992000	-2.220524000	-0.609597000

N	7.408342000	-1.798406000	-0.197002000
C	6.310549000	-2.468423000	-0.597101000
H	5.357432000	-2.084641000	-0.253061000
H	7.712490000	-4.884995000	-2.503702000
H	9.750524000	-3.640988000	-1.751881000
H	9.483716000	-1.656366000	-0.268385000
C	0.191915000	-4.641802000	-3.690189000
C	0.520337000	-3.455188000	-4.596703000
C	-0.478732000	-2.384170000	-4.091487000
C	-1.668056000	-3.206121000	-3.645515000
H	-0.725482000	-1.651418000	-4.867884000
H	-0.054556000	-1.824150000	-3.244060000
C	-1.287501000	-4.560037000	-3.479171000
C	0.263003000	-3.749076000	-6.088821000
H	1.550919000	-3.123463000	-4.455107000
H	-0.793171000	-3.983158000	-6.262056000
H	0.850565000	-4.595774000	-6.450322000
H	0.516766000	-2.875150000	-6.700415000
C	-2.282919000	-5.524449000	-3.224515000
C	-3.583123000	-5.052161000	-2.993446000
C	-3.944169000	-3.695930000	-3.057156000
C	-2.967837000	-2.740771000	-3.443582000
H	-4.349950000	-5.785155000	-2.753618000
C	-2.051926000	-7.016514000	-3.281683000
H	-1.199808000	-7.256455000	-3.921693000
H	-2.931741000	-7.520940000	-3.693609000
H	-1.862484000	-7.457152000	-2.296249000
C	0.799350000	-6.274826000	-1.794810000
H	1.246350000	-4.953936000	-0.097079000
H	2.059519000	-6.514503000	0.010590000
C	1.138221000	-7.747888000	-2.101367000
H	-0.244587000	-6.190196000	-1.484895000
H	0.907163000	-8.379968000	-1.236644000
H	2.204756000	-7.862189000	-2.324592000
H	0.579455000	-8.130365000	-2.958746000
C	-3.280533000	-1.284842000	-3.716640000
H	-4.340871000	-1.117288000	-3.920497000
H	-2.727898000	-0.926097000	-4.589648000
H	-2.989916000	-0.630061000	-2.882123000
C	-5.352736000	-3.324378000	-2.758780000
C	-5.662576000	-2.199145000	-1.979645000
N	-6.923281000	-1.810463000	-1.698540000
C	-7.960413000	-2.546552000	-2.145297000
C	-7.750191000	-3.696034000	-2.900444000
C	-6.449084000	-4.078324000	-3.218637000
H	-6.279229000	-4.950909000	-3.841524000
H	-4.871627000	-1.586208000	-1.565512000
H	-8.957115000	-2.202331000	-1.893642000

H	-8.602706000	-4.268336000	-3.250715000
H	-9.248359000	4.340863000	1.291784000
C	-8.331674000	3.775386000	1.161547000
H	-9.287537000	2.162498000	0.070917000
H	-7.094444000	5.164818000	2.243840000
C	-8.366557000	2.562637000	0.479566000
C	-7.124192000	4.235195000	1.683898000
H	-5.180165000	1.423594000	3.080810000
C	-4.101787000	1.590522000	3.023508000
H	-3.692468000	0.888835000	2.281657000
N	-7.245260000	1.836660000	0.300562000
H	-3.679101000	1.294351000	3.986343000
C	-5.946023000	3.485839000	1.510012000
C	-6.075019000	2.291603000	0.787854000
C	-3.751016000	3.027468000	2.706504000
C	-4.624365000	3.919958000	2.030989000
H	-1.895513000	2.203144000	4.754267000
H	-1.092666000	3.718961000	6.617873000
C	-1.473666000	2.820227000	3.952699000
C	-2.528155000	3.540197000	3.137522000
H	-5.204810000	1.679648000	0.587002000
H	-2.250202000	4.731893000	5.739320000
C	-1.186988000	4.488500000	5.843039000
C	-4.250565000	5.264942000	1.874448000
H	-0.869945000	2.155527000	3.316897000
H	-4.951206000	5.950172000	1.402572000
C	-0.605535000	3.980866000	4.508860000
C	-2.111239000	4.865630000	2.873008000
H	-0.676702000	5.387090000	6.197500000
C	-3.010962000	5.775350000	2.286989000
H	0.426676000	3.656224000	4.654565000
C	-0.711574000	4.999240000	3.373456000
H	-3.667449000	7.825894000	2.314358000
C	-2.744528000	7.256977000	2.163309000
H	1.774317000	4.792619000	6.150768000
H	1.242052000	6.305133000	5.404064000
C	0.345066000	5.549459000	2.720435000
C	2.084323000	5.615823000	5.496615000
H	-2.019084000	7.589123000	2.910128000
H	-2.354741000	7.533965000	1.176826000
H	-0.659198000	6.023464000	0.849390000
C	1.781329000	5.234525000	2.966849000
C	2.550612000	5.139126000	4.140949000
H	1.009942000	4.474725000	0.130951000
C	0.309863000	6.181110000	1.329762000
H	2.894349000	6.144393000	6.010029000
C	1.425427000	5.380001000	0.598581000
C	3.847261000	4.627382000	4.002425000

C	2.387732000	5.016102000	1.709638000
H	4.440754000	4.488532000	4.903393000
C	0.650413000	7.684265000	1.338340000
H	-0.052632000	8.260567000	1.944685000
C	4.401724000	4.260919000	2.765318000
C	3.683305000	4.517227000	1.570170000
H	1.897734000	5.964238000	-0.199916000
H	3.840483000	3.464452000	-0.337652000
H	5.097768000	1.974289000	1.471336000
C	5.721364000	3.580412000	2.775879000
C	5.905097000	2.385423000	2.066191000
H	1.653817000	7.854323000	1.743971000
C	4.267177000	4.331948000	0.185461000
H	0.627947000	8.087430000	0.319426000
C	6.814114000	4.032046000	3.536530000
N	7.052772000	1.682081000	2.086104000
H	6.734674000	4.959282000	4.095680000
C	8.092218000	2.124577000	2.823983000
C	7.999507000	3.299905000	3.561680000
H	5.353497000	4.213258000	0.202174000
H	4.049745000	5.204298000	-0.440004000
H	8.994940000	1.524308000	2.816878000
H	8.853061000	3.634992000	4.141483000
H	-8.388048000	2.181807000	-5.186902000
H	-9.703874000	-0.655056000	0.860720000
H	-8.766010000	0.890302000	-3.085704000
H	-10.298482000	-1.647913000	3.064899000
C	-7.561996000	1.994054000	-4.509136000
C	-8.937967000	-1.041673000	1.524149000
C	-7.784991000	1.275310000	-3.339884000
C	-9.258976000	-1.594298000	2.758834000
C	-6.283128000	2.474658000	-4.782248000
N	-7.659039000	-0.960939000	1.098753000
H	-6.101567000	3.056808000	-5.680389000
N	-6.782770000	1.029161000	-2.470438000
C	-8.237849000	-2.069356000	3.584632000
H	-8.472025000	-2.501259000	4.553052000
C	-5.226979000	2.224960000	-3.887951000
C	-6.676859000	-1.435063000	1.889618000
H	-3.956801000	2.019401000	-6.201329000
C	-5.544609000	1.484376000	-2.739491000
C	-6.906761000	-1.997057000	3.152508000
C	-3.307936000	2.443670000	-5.438349000
H	-2.384633000	2.862421000	-7.902950000
H	-5.665134000	-1.361371000	1.506878000
H	-1.267764000	1.564849000	-7.490757000
C	-3.831987000	2.662331000	-4.153228000
H	-6.208118000	-0.734401000	6.449905000

H	-4.774369000	1.237594000	-2.018957000
C	-1.560386000	2.589977000	-7.235950000
H	-4.962362000	0.284191000	5.742783000
C	-5.705692000	-0.482553000	5.506492000
C	-5.717433000	-2.495122000	3.910431000
C	-1.984021000	2.737020000	-5.793201000
C	-5.073520000	-1.713677000	4.898048000
H	-5.763856000	-4.372715000	2.868117000
H	-4.639942000	3.763408000	-1.779077000
C	-3.028462000	3.294064000	-3.170269000
H	-0.715119000	3.243078000	-7.465222000
C	-5.189706000	-3.739425000	3.541523000
H	-6.458447000	-0.030948000	4.853703000
C	-3.548361000	3.729060000	-1.816667000
H	0.154760000	1.064710000	-5.794608000
C	-1.149392000	3.235310000	-4.774714000
H	1.588828000	0.396622000	-7.713017000
H	1.172367000	2.105437000	-7.913221000
C	-1.706836000	3.574100000	-3.519786000
C	1.706534000	1.395577000	-7.277473000
C	-3.812455000	-2.150494000	5.316328000
C	1.189943000	1.412736000	-5.824951000
H	-3.188797000	4.732953000	-1.569938000
H	-3.202979000	3.073413000	-1.004663000
C	-3.962671000	-4.213761000	4.023051000
H	-4.409146000	-6.310336000	3.945888000
H	-2.453170000	-0.544672000	5.828312000
C	0.307311000	3.557911000	-4.720869000
C	-3.210140000	-3.322341000	4.810617000
H	-0.917582000	6.108521000	-4.663219000
C	-2.872217000	-1.455979000	6.281479000
C	-3.562747000	-5.644309000	3.745313000
H	2.512085000	-0.327578000	-5.473246000
C	2.096768000	0.527088000	-4.926681000
H	2.770505000	1.654028000	-7.315274000
C	1.333464000	2.777685000	-5.151954000
C	-0.671465000	4.272768000	-2.665001000
H	1.525611000	0.119710000	-4.079021000
H	-3.263440000	-5.811511000	2.704037000
H	-3.364798000	-1.152726000	7.211894000
C	0.105426000	6.072913000	-4.272746000
C	0.421588000	4.680925000	-3.689972000
H	-0.259984000	3.584001000	-1.911586000
H	-1.073732000	5.136496000	-2.123376000
C	-1.749213000	-2.508246000	6.530310000
H	0.779225000	6.339733000	-5.090113000
H	-2.738050000	-5.956120000	4.389205000
C	-1.779742000	-3.311788000	5.232208000

C	2.747890000	2.823529000	-4.675748000
C	3.158544000	1.490898000	-4.443591000
H	0.194417000	6.841606000	-3.496376000
H	4.528152000	-0.858636000	-4.582531000
H	2.686645000	5.328814000	-5.740066000
H	1.409056000	4.689310000	-3.222798000
C	-0.733428000	-3.579970000	4.405539000
C	-0.891006000	-3.981812000	2.937538000
C	4.748018000	-0.294810000	-3.669783000
C	4.376331000	1.162240000	-3.847910000
C	3.659324000	3.876973000	-4.471296000
C	3.428258000	5.295017000	-4.938579000
H	5.810786000	-0.433393000	-3.457085000
H	0.338270000	-3.261250000	1.254595000
H	4.358848000	5.722769000	-5.325460000
C	0.008559000	-2.929826000	2.244839000
H	0.553522000	-4.596176000	6.877029000
H	4.176537000	-0.778622000	-2.865306000
H	3.081417000	5.955652000	-4.135500000
C	0.703610000	-3.185258000	4.522298000
C	4.877901000	3.548877000	-3.860241000
C	5.228922000	2.238838000	-3.496052000
H	-0.538968000	-1.985332000	2.108102000
C	1.318509000	-3.817607000	6.940234000
H	0.949641000	-3.048855000	7.628631000
C	1.139261000	-2.765942000	3.240337000
C	1.636487000	-3.261279000	5.573854000
H	7.803176000	3.207587000	-3.962354000
H	5.576885000	4.355061000	-3.648050000
C	6.478534000	2.036000000	-2.718662000
H	2.210505000	-4.256357000	7.397521000
C	7.713457000	2.616901000	-3.055686000
H	2.057351000	-2.257228000	0.837137000
C	2.442525000	-2.352634000	2.963911000
C	2.956552000	-2.888515000	5.282566000
C	2.778665000	-1.881706000	1.564990000
C	3.392552000	-2.447025000	4.020973000
H	2.757401000	-0.784829000	1.488221000
C	6.447869000	1.277528000	-1.541054000
H	3.683787000	-2.962772000	6.086837000
C	8.824485000	2.419656000	-2.236510000
H	5.521844000	0.818777000	-1.215025000
H	9.786589000	2.855304000	-2.484817000
H	4.756271000	-0.552750000	2.477295000
H	3.766088000	-2.218931000	1.240471000
C	4.853920000	-2.261602000	3.807166000
C	5.389997000	-1.295919000	2.940213000
N	7.521665000	1.083086000	-0.752165000

C	8.701239000	1.644016000	-1.087610000
C	5.794935000	-3.110388000	4.427438000
H	5.453944000	-3.890141000	5.100195000
N	6.700001000	-1.226816000	2.620111000
H	9.540582000	1.466727000	-0.424657000
C	7.154310000	-2.985714000	4.157960000
C	7.579711000	-2.052154000	3.218308000
H	7.880731000	-3.634152000	4.636212000
H	8.620065000	-1.964718000	2.926854000
Pd	7.218382000	-0.061628000	0.964589000
Pd	-7.187487000	0.004503000	-0.696244000
C	-0.376346000	-5.402648000	2.630635000
H	0.699284000	-5.478756000	2.824685000
H	-0.872594000	-6.160966000	3.239399000
H	-0.546700000	-5.651849000	1.576571000
H	-1.926502000	-3.884704000	2.608130000
C	-2.087625000	-3.357676000	7.771677000
H	-3.087733000	-3.795999000	7.682081000
H	-1.378954000	-4.177009000	7.915802000
H	-2.073460000	-2.735336000	8.673105000
H	-0.783250000	-2.016087000	6.668857000

$(S,S)_2(R,R)_2$

Sum of electronic and thermal free energies: -5952.730598

C	4.997119000	2.032553000	-4.182062000
C	3.816062000	2.706126000	-4.504501000
C	3.468394000	3.956658000	-3.942709000
C	4.427786000	4.666489000	-3.193570000
C	5.580391000	3.962074000	-2.814257000
C	5.841977000	2.648541000	-3.228407000
H	6.301145000	4.460623000	-2.169034000
C	5.391592000	0.770503000	-4.917674000
H	4.527005000	0.154240000	-5.176325000
H	6.093397000	0.151600000	-4.350820000
H	5.891036000	1.027998000	-5.861330000
C	4.327962000	6.137867000	-2.861387000
H	5.294251000	6.623789000	-3.033633000
H	4.055746000	6.327641000	-1.816620000
H	3.590714000	6.634762000	-3.495068000
C	2.729368000	2.234453000	-5.448091000
C	2.047279000	4.237678000	-4.313850000
C	6.979351000	1.925662000	-2.584389000
C	8.328509000	2.278558000	-2.734223000
C	9.309830000	1.586711000	-2.022083000
C	8.935280000	0.563548000	-1.158341000

N	7.639838000	0.218687000	-0.997163000
C	6.694893000	0.877626000	-1.696835000
H	5.667506000	0.571985000	-1.533098000
H	8.605124000	3.084524000	-3.407433000
H	10.360369000	1.835576000	-2.129659000
H	9.668260000	0.012347000	-0.580133000
C	1.085246000	4.713106000	-3.476630000
C	1.339072000	5.155447000	-2.034592000
C	0.257649000	4.361828000	-1.264234000
C	-0.881457000	4.294696000	-2.257000000
H	-0.015497000	4.846228000	-0.320453000
H	0.617143000	3.351310000	-1.016597000
C	-0.402447000	4.558316000	-3.564519000
C	1.123681000	6.668513000	-1.823422000
H	2.339618000	4.872035000	-1.703887000
H	0.086951000	6.947099000	-2.042093000
H	1.767760000	7.272302000	-2.466092000
H	1.331254000	6.942797000	-0.782237000
C	-1.331835000	4.694944000	-4.615646000
C	-2.677737000	4.432978000	-4.317901000
C	-3.145017000	4.096602000	-3.036433000
C	-2.223530000	4.060741000	-1.957788000
H	-3.396055000	4.507950000	-5.130977000
C	-0.982989000	5.200883000	-5.995726000
H	-0.107446000	5.853453000	-5.963119000
H	-1.814865000	5.778112000	-6.411240000
H	-0.768779000	4.392867000	-6.704377000
C	1.822962000	3.484703000	-5.623654000
H	2.159216000	1.405073000	-5.002570000
H	3.123652000	1.873562000	-6.404424000
C	2.278048000	4.292927000	-6.856166000
H	0.777474000	3.190154000	-5.737097000
H	2.104912000	3.718975000	-7.773265000
H	3.348873000	4.517539000	-6.798253000
H	1.746383000	5.243174000	-6.946314000
C	-2.635577000	3.872344000	-0.514543000
H	-3.677284000	4.152754000	-0.342303000
H	-2.027966000	4.490192000	0.151477000
H	-2.503123000	2.834603000	-0.174150000
C	-4.604564000	3.880797000	-2.844138000
C	-5.106081000	2.843958000	-2.039590000
N	-6.420259000	2.661009000	-1.795859000
C	-7.322957000	3.474012000	-2.381095000
C	-6.922978000	4.499694000	-3.230593000
C	-5.564620000	4.712587000	-3.450689000
H	-5.241325000	5.540848000	-4.073177000
H	-4.431669000	2.135560000	-1.574396000
H	-8.368357000	3.297884000	-2.154244000

H	-7.671365000	5.138576000	-3.687423000
H	-10.360368000	-1.835575000	2.129660000
C	-9.309830000	-1.586710000	2.022083000
H	-9.668260000	-0.012345000	0.580133000
H	-8.605123000	-3.084524000	3.407431000
C	-8.935279000	-0.563546000	1.158341000
C	-8.328508000	-2.278558000	2.734221000
H	-5.891038000	-1.028003000	5.861329000
C	-5.391594000	-0.770507000	4.917674000
H	-4.527008000	-0.154244000	5.176325000
N	-7.639837000	-0.218687000	0.997161000
H	-6.093399000	-0.151605000	4.350820000
C	-6.979350000	-1.925663000	2.584386000
C	-6.694891000	-0.877627000	1.696832000
C	-4.997120000	-2.032556000	4.182061000
C	-5.580387000	-3.962074000	2.814249000
H	-2.159219000	-1.405077000	5.002578000
C	-2.729372000	-2.234459000	5.448095000
C	-3.816063000	-2.706130000	4.504501000
H	-5.667505000	-0.571987000	1.533094000
C	-5.841976000	-2.648543000	3.228404000
H	-3.123658000	-1.873571000	6.404428000
H	-6.301139000	-4.460621000	2.169022000
C	-1.822965000	-3.484709000	5.623655000
C	-3.468393000	-3.956659000	3.942705000
C	-4.427782000	-4.666489000	3.193561000
C	-2.047278000	-4.237680000	4.313848000
H	-4.055738000	-6.327634000	1.816604000
C	-4.327954000	-6.137865000	2.861372000
H	0.107449000	-5.853468000	5.963111000
H	0.768777000	-4.392884000	6.704378000
C	-1.085244000	-4.713104000	3.476628000
C	0.982990000	-5.200895000	5.995722000
H	-0.086949000	-6.947091000	2.042083000
H	-5.294243000	-6.623790000	3.033616000
C	0.402449000	-4.558315000	3.564519000
C	1.331836000	-4.694948000	4.615646000
H	0.015499000	-4.846211000	0.320451000
C	-1.339070000	-5.155439000	2.034588000
H	1.814867000	-5.778125000	6.411234000
C	-0.257647000	-4.361816000	1.264235000
C	2.677739000	-4.432981000	4.317901000
C	0.881458000	-4.294688000	2.257001000
H	3.396056000	-4.507958000	5.130977000
C	3.145019000	-4.096600000	3.036435000
C	2.223532000	-4.060732000	1.957791000
H	-0.617141000	-3.351296000	1.016602000
H	2.027974000	-4.490169000	-0.151477000

H	4.431669000	-2.135559000	1.574397000
C	4.604566000	-3.880794000	2.844140000
C	5.106082000	-2.843957000	2.039591000
C	2.635582000	-3.872324000	0.514547000
C	5.564622000	-4.712585000	3.450690000
N	6.420260000	-2.661007000	1.795859000
H	5.241327000	-5.540847000	4.073177000
C	7.322958000	-3.474010000	2.381094000
C	6.922979000	-4.499693000	3.230593000
H	3.677290000	-4.152729000	0.342309000
H	2.503125000	-2.834581000	0.174161000
H	8.368358000	-3.297882000	2.154243000
H	7.671367000	-5.138575000	3.687422000
H	-8.204035000	4.650179000	3.282210000
H	-9.298769000	0.556874000	-2.375217000
H	-8.668642000	3.093210000	1.384427000
H	-9.657710000	-1.063357000	-4.237231000
C	-7.389100000	4.114947000	2.806460000
C	-8.536489000	-0.165394000	-2.645793000
C	-7.660157000	3.253906000	1.747892000
C	-8.723959000	-1.070600000	-3.684825000
C	-6.078849000	4.253018000	3.258213000
N	-7.390879000	-0.148127000	-1.932753000
H	-5.865983000	4.890136000	4.110772000
N	-6.668389000	2.577534000	1.134926000
C	-7.709881000	-1.975857000	-3.996452000
H	-7.844240000	-2.691676000	-4.801859000
C	-5.031392000	3.547530000	2.635290000
C	-6.414016000	-1.024550000	-2.230151000
H	-3.799448000	5.775306000	3.391469000
C	-5.397603000	2.734208000	1.552856000
C	-6.514380000	-1.970409000	-3.259309000
C	-3.140421000	4.915214000	3.483312000
H	-2.309483000	7.065191000	4.811048000
H	-5.508064000	-0.958995000	-1.638576000
H	-1.034154000	7.121331000	3.597394000
C	-3.623793000	3.651875000	3.101276000
H	-5.249580000	-4.922010000	-0.982278000
H	-4.646397000	2.195038000	0.992932000
C	-1.444656000	6.521416000	4.418127000
H	-4.648175000	-3.370401000	-0.417065000
C	-5.246548000	-3.848749000	-1.204854000
C	-5.342001000	-2.827020000	-3.573777000
C	-1.844424000	5.135298000	3.972153000
C	-4.682677000	-3.601385000	-2.587544000
H	-5.406431000	-2.252156000	-5.645218000
H	-4.277513000	0.943798000	3.002218000
C	-2.786845000	2.513075000	3.252359000

H	-0.686973000	6.477904000	5.204943000
C	-4.843200000	-2.790163000	-4.885609000
H	-6.276803000	-3.498117000	-1.104322000
C	-3.204537000	1.107124000	2.884840000
H	0.556540000	5.748477000	2.632827000
C	-1.011941000	4.008040000	4.094378000
H	2.044131000	7.730176000	2.883640000
H	1.352114000	7.341936000	4.466447000
C	-1.522277000	2.722432000	3.800144000
C	2.011143000	6.973814000	3.675907000
C	-3.471051000	-4.187315000	-2.955821000
C	1.532109000	5.623192000	3.110345000
H	-2.695801000	0.369390000	3.508345000
H	-2.939427000	0.864196000	1.844648000
C	-3.626252000	-3.378319000	-5.253929000
H	-4.054625000	-3.629495000	-7.345765000
H	-2.072407000	-4.452869000	-1.324971000
C	0.392191000	3.830385000	4.564936000
C	-2.888178000	-3.991373000	-4.225855000
H	-1.188796000	2.718564000	6.642600000
C	-2.572582000	-5.054613000	-2.098464000
C	-3.203994000	-3.374613000	-6.704582000
H	3.121807000	5.849668000	1.579923000
C	2.572581000	5.054615000	2.098468000
H	3.018309000	6.885704000	4.098016000
C	1.493963000	4.510813000	4.156122000
C	-0.514506000	1.665635000	4.206600000
H	2.072406000	4.452872000	1.324974000
H	-2.837260000	-2.395240000	-7.033801000
H	-3.121808000	-5.849666000	-1.579917000
C	-0.146892000	2.380643000	6.617205000
C	0.416704000	2.432360000	5.184193000
H	0.054513000	1.302551000	3.337874000
H	-0.975017000	0.791487000	4.680717000
C	-1.532110000	-5.623192000	-3.110341000
H	0.421569000	3.014121000	7.302312000
H	-2.415658000	-4.107784000	-6.889174000
C	-1.493964000	-4.510814000	-4.156118000
C	2.888176000	3.991370000	4.225857000
C	3.471050000	4.187315000	2.955823000
H	-0.116590000	1.354884000	7.002871000
H	5.249575000	4.922014000	0.982281000
H	2.415654000	4.107774000	6.889176000
H	1.423441000	2.010320000	5.169472000
C	-0.392192000	-3.830387000	-4.564933000
C	-0.416705000	-2.432362000	-5.184191000
C	5.246547000	3.848753000	1.204856000
C	4.682676000	3.601386000	2.587546000

C	3.626250000	3.378314000	5.253930000
C	3.203990000	3.374604000	6.704582000
H	6.276805000	3.498126000	1.104326000
H	0.975016000	-0.791489000	-4.680715000
H	4.054621000	3.629486000	7.345767000
C	0.514505000	-1.665637000	-4.206598000
H	0.686971000	-6.477906000	-5.204939000
H	4.648178000	3.370403000	0.417067000
H	2.837258000	2.395230000	7.033799000
C	1.011939000	-4.008042000	-4.094376000
C	4.843199000	2.790160000	4.885609000
C	5.342000000	2.827019000	3.573778000
H	-0.054514000	-1.302553000	-3.337872000
C	1.444655000	-6.521419000	-4.418124000
H	1.034153000	-7.121334000	-3.597390000
C	1.522275000	-2.722434000	-3.800142000
C	1.844422000	-5.135301000	-3.972149000
H	7.844240000	2.691677000	4.801858000
H	5.406430000	2.252151000	5.645217000
C	6.514379000	1.970409000	3.259309000
H	2.309481000	-7.065193000	-4.811046000
C	7.709881000	1.975859000	3.996451000
H	2.695800000	-0.369392000	-3.508340000
C	2.786844000	-2.513078000	-3.252356000
C	3.140419000	-4.915217000	-3.483308000
C	3.204535000	-1.107126000	-2.884836000
C	3.623792000	-3.651878000	-3.101273000
H	2.939425000	-0.864199000	-1.844644000
C	6.414016000	1.024552000	2.230150000
H	3.799447000	-5.775308000	-3.391466000
C	8.723960000	1.070602000	3.684823000
H	5.508063000	0.958996000	1.638576000
H	9.657711000	1.063360000	4.237228000
H	4.646397000	-2.195038000	-0.992930000
H	4.277511000	-0.943801000	-3.002212000
C	5.031390000	-3.547532000	-2.635286000
C	5.397602000	-2.734209000	-1.552854000
N	7.390879000	0.148129000	1.932751000
C	8.536490000	0.165396000	2.645790000
C	6.078847000	-4.253020000	-3.258210000
H	5.865981000	-4.890139000	-4.110769000
N	6.668389000	-2.577534000	-1.134926000
H	9.298770000	-0.556871000	2.375214000
C	7.389098000	-4.114948000	-2.806458000
C	7.660157000	-3.253907000	-1.747892000
H	8.204034000	-4.650181000	-3.282208000
H	8.668641000	-3.093209000	-1.384428000
Pd	7.055670000	-1.227026000	0.405745000

Pd	-7.055670000	1.227027000	-0.405746000
C	0.146891000	-2.380646000	-6.617202000
H	1.188795000	-2.718567000	-6.642597000
H	-0.421570000	-3.014124000	-7.302309000
H	0.116589000	-1.354887000	-7.002868000
H	-1.423442000	-2.010322000	-5.169470000
C	-2.011144000	-6.973815000	-3.675901000
H	-3.018311000	-6.885705000	-4.098010000
H	-1.352115000	-7.341938000	-4.466441000
H	-2.044133000	-7.730176000	-2.883632000
H	-0.556541000	-5.748476000	-2.632822000
C	-2.278052000	-4.292937000	6.856164000
H	-3.348877000	-4.517549000	6.798248000
H	-1.746387000	-5.243184000	6.946309000
H	-2.104917000	-3.718988000	7.773265000
H	-0.777477000	-3.190159000	5.737101000
H	-2.339616000	-4.872025000	1.703884000
C	-1.123679000	-6.668503000	1.823411000
H	-3.590706000	-6.634762000	3.495051000
H	-1.767759000	-7.272296000	2.466077000
H	-1.331250000	-6.942782000	0.782225000

(*S,S*)(*R,R*)(*S,S*)(*R,R*)
Sum of electronic and thermal free energies: -5952.716101

C	4.131317000	-2.414031000	-4.927223000
C	2.852011000	-2.224512000	-5.459759000
C	2.385531000	-0.962876000	-5.885252000
C	3.280237000	0.119071000	-5.985147000
C	4.539361000	-0.053009000	-5.395608000
C	4.950557000	-1.265346000	-4.824802000
H	5.234982000	0.783744000	-5.403122000
C	4.582566000	-3.793047000	-4.501588000
H	3.793005000	-4.331987000	-3.967288000
H	5.463971000	-3.765496000	-3.855062000
H	4.842330000	-4.403557000	-5.376285000
C	2.987809000	1.390297000	-6.748315000
H	3.830811000	1.631095000	-7.405191000
H	2.833993000	2.256341000	-6.093891000
H	2.101249000	1.279228000	-7.375870000
C	1.761346000	-3.264941000	-5.615433000
C	0.915143000	-1.079737000	-6.104915000
C	6.233356000	-1.270293000	-4.056998000
C	7.454014000	-1.763560000	-4.538024000
C	8.592960000	-1.694849000	-3.733944000
C	8.494130000	-1.154527000	-2.457039000

N	7.323036000	-0.682050000	-1.978677000
C	6.230193000	-0.726980000	-2.765034000
H	5.313502000	-0.324055000	-2.349803000
H	7.511754000	-2.187868000	-5.535927000
H	9.552669000	-2.057126000	-4.087196000
H	9.354905000	-1.094849000	-1.800270000
C	-0.005456000	-0.193189000	-5.641453000
C	0.349311000	1.079476000	-4.869666000
C	-0.497574000	0.891908000	-3.587370000
C	-1.743158000	0.210100000	-4.115308000
H	-0.697277000	1.844196000	-3.084578000
H	0.030439000	0.248210000	-2.867988000
C	-1.459445000	-0.398805000	-5.362249000
C	-0.065218000	2.375355000	-5.594606000
H	1.412968000	1.114845000	-4.629769000
H	-1.150869000	2.413513000	-5.737311000
H	0.397578000	2.461969000	-6.579908000
H	0.228071000	3.252946000	-5.005963000
C	-2.512207000	-0.957533000	-6.111467000
C	-3.793868000	-0.912101000	-5.542621000
C	-4.075665000	-0.352065000	-4.284817000
C	-3.009398000	0.216718000	-3.532178000
H	-4.614129000	-1.330073000	-6.120941000
C	-2.349491000	-1.498760000	-7.511114000
H	-1.547310000	-0.978600000	-8.041415000
H	-3.272369000	-1.371842000	-8.085264000
H	-2.110474000	-2.568383000	-7.519691000
C	0.677822000	-2.542712000	-6.472179000
H	1.350533000	-3.550650000	-4.634960000
H	2.118200000	-4.185516000	-6.090759000
C	0.904417000	-2.832226000	-7.969229000
H	-0.324431000	-2.866059000	-6.179662000
H	0.744192000	-3.895357000	-8.179557000
H	1.929882000	-2.582649000	-8.263344000
H	0.227989000	-2.256243000	-8.605782000
C	-3.184348000	0.868603000	-2.177754000
H	-4.152449000	1.363918000	-2.083559000
H	-2.419277000	1.627230000	-2.001407000
H	-3.097930000	0.140454000	-1.357478000
C	-5.496258000	-0.245626000	-3.854916000
C	-5.892776000	-0.396652000	-2.517852000
N	-7.145744000	-0.152657000	-2.077499000
C	-8.111725000	0.168255000	-2.958585000
C	-7.833176000	0.254206000	-4.319800000
C	-6.525591000	0.074896000	-4.763512000
H	-6.292045000	0.212938000	-5.814299000
H	-5.184442000	-0.716363000	-1.765766000
H	-9.101145000	0.364512000	-2.561553000

H	-8.629473000	0.501349000	-5.013914000
H	-9.569727000	-4.618825000	-0.276585000
C	-8.626403000	-4.126604000	-0.064566000
H	-9.417086000	-2.133292000	-0.366336000
H	-7.528078000	-5.946925000	0.304371000
C	-8.554181000	-2.738984000	-0.113320000
C	-7.485985000	-4.862764000	0.258868000
H	-4.992771000	-4.739706000	4.018055000
C	-4.821658000	-3.998879000	3.226216000
H	-4.138107000	-3.250169000	3.641597000
N	-7.399869000	-2.087086000	0.146281000
H	-5.782376000	-3.514187000	3.034318000
C	-6.281514000	-4.198564000	0.536762000
C	-6.300970000	-2.798671000	0.459426000
C	-4.250327000	-4.674003000	1.998317000
C	-4.482960000	-5.743339000	-0.180411000
H	-1.576591000	-4.059776000	3.255063000
C	-1.966071000	-5.085188000	3.174285000
C	-2.956044000	-5.197179000	2.035356000
H	-5.394315000	-2.234605000	0.640817000
C	-4.991958000	-4.891981000	0.812310000
H	-2.408858000	-5.332116000	4.146054000
H	-5.106642000	-5.973420000	-1.041864000
C	-0.832703000	-6.074199000	2.776676000
C	-2.383374000	-5.903146000	0.954764000
C	-3.195842000	-6.296084000	-0.126478000
C	-0.928827000	-6.068201000	1.252075000
H	-2.519990000	-6.865113000	-2.125340000
C	-2.789158000	-7.317190000	-1.163649000
H	1.510987000	-7.924279000	1.919309000
H	1.898769000	-7.034987000	3.394941000
C	0.086449000	-5.833278000	0.378203000
C	2.250326000	-7.266616000	2.383068000
H	1.491101000	-6.642326000	-1.951576000
H	-3.618518000	-8.006209000	-1.355444000
C	1.511127000	-5.486806000	0.672984000
C	2.473402000	-6.029192000	1.546437000
H	1.087310000	-3.947596000	-2.191048000
C	-0.110262000	-5.431902000	-1.084376000
H	3.182170000	-7.830729000	2.489202000
C	0.753041000	-4.148973000	-1.167387000
C	3.739130000	-5.424996000	1.556046000
C	1.888292000	-4.443526000	-0.208544000
H	4.480798000	-5.829618000	2.240294000
C	4.099373000	-4.343175000	0.734499000
C	3.145175000	-3.838763000	-0.192573000
H	0.177141000	-3.271558000	-0.836941000
H	2.827780000	-2.844713000	-2.086328000

H	5.017098000	-1.741702000	0.554884000
C	5.496378000	-3.836623000	0.802485000
C	5.804920000	-2.475575000	0.656511000
C	3.441656000	-2.739776000	-1.190104000
C	6.599501000	-4.693934000	0.991639000
N	7.064130000	-1.991380000	0.619837000
H	6.440554000	-5.762497000	1.094214000
C	8.103986000	-2.826134000	0.805154000
C	7.895811000	-4.186158000	1.017450000
H	4.484960000	-2.744578000	-1.516096000
H	3.222101000	-1.740411000	-0.784581000
H	9.098598000	-2.395700000	0.776085000
H	8.748270000	-4.840046000	1.168342000
H	-8.629478000	-0.501346000	5.013909000
H	-9.417083000	2.133295000	0.366335000
H	-9.101147000	-0.364507000	2.561547000
H	-9.569723000	4.618829000	0.276587000
C	-7.833180000	-0.254204000	4.319796000
C	-8.554178000	2.738987000	0.113319000
C	-8.111727000	-0.168251000	2.958581000
C	-8.626400000	4.126607000	0.064567000
C	-6.525595000	-0.074898000	4.763510000
N	-7.399867000	2.087090000	-0.146284000
H	-6.292051000	-0.212941000	5.814297000
N	-7.145744000	0.152660000	2.077496000
C	-7.485982000	4.862767000	-0.258868000
H	-7.528075000	5.946929000	-0.304370000
C	-5.496261000	0.245624000	3.854916000
C	-6.300968000	2.798674000	-0.459430000
H	-4.614134000	1.330066000	6.120944000
C	-5.892776000	0.396652000	2.517852000
C	-6.281511000	4.198567000	-0.536764000
C	-3.793873000	0.912093000	5.542624000
H	-3.272376000	1.371829000	8.085269000
H	-5.394313000	2.234609000	-0.640824000
H	-2.110478000	2.568369000	7.519700000
C	-4.075668000	0.352060000	4.284819000
H	-4.992752000	4.739703000	-4.018068000
H	-5.184440000	0.716362000	1.765767000
C	-2.349498000	1.498746000	7.511120000
H	-4.138117000	3.250156000	-3.641589000
C	-4.821658000	3.998882000	-3.226219000
C	-4.991955000	4.891985000	-0.812314000
C	-2.512213000	0.957522000	6.111472000
C	-4.250326000	4.674008000	-1.998322000
H	-5.106638000	5.973424000	1.041861000
H	-4.152453000	-1.363921000	2.083560000
C	-3.009402000	-0.216722000	3.532180000

H	-1.547317000	0.978584000	8.041421000
C	-4.482957000	5.743344000	0.180406000
H	-5.782386000	3.514208000	-3.034325000
C	-3.184352000	-0.868605000	2.177755000
H	-0.324437000	2.866044000	6.179680000
C	-1.459450000	0.398795000	5.362254000
H	0.744189000	3.895335000	8.179577000
H	0.227986000	2.256220000	8.605797000
C	-1.743162000	-0.210106000	4.115311000
C	0.904414000	2.832206000	7.969245000
C	-2.956043000	5.197185000	-2.035362000
C	0.677816000	2.542697000	6.472194000
H	-2.419282000	-1.627232000	2.001406000
H	-3.097935000	-0.140454000	1.357481000
C	-3.195840000	6.296090000	0.126471000
H	-3.618517000	8.006218000	1.355433000
H	-1.576592000	4.059781000	-3.255071000
C	-0.005461000	0.193177000	5.641459000
C	-2.383373000	5.903152000	-0.954771000
H	-1.150881000	-2.413523000	5.737305000
C	-1.966071000	5.085193000	-3.174292000
C	-2.789157000	7.317198000	1.163641000
H	2.118193000	4.185503000	6.090779000
C	1.761339000	3.264929000	5.615449000
H	1.929879000	2.582627000	8.263357000
C	0.915138000	1.079723000	6.104925000
C	-0.497578000	-0.891913000	3.587372000
H	1.350526000	3.550641000	4.634978000
H	-2.519991000	6.865122000	2.125334000
H	-2.408859000	5.332120000	-4.146061000
C	-0.065229000	-2.375366000	5.594605000
C	0.349304000	-1.079486000	4.869669000
H	0.030438000	-0.248212000	2.867994000
H	-0.697280000	-1.844198000	3.084576000
C	-0.832703000	6.074202000	-2.776685000
H	0.397563000	-2.461982000	6.579908000
H	-1.938184000	7.909663000	0.820793000
C	-0.928825000	6.068207000	-1.252083000
C	2.385526000	0.962864000	5.885261000
C	2.852005000	2.224501000	5.459771000
H	0.228061000	-3.252956000	5.005962000
H	4.842314000	4.403551000	5.376302000
H	2.101250000	-1.279241000	7.375878000
H	1.412962000	-1.114857000	4.629774000
C	0.086451000	5.833286000	-0.378211000
C	-0.110258000	5.431916000	1.084370000
C	4.582558000	3.793041000	4.501603000
C	4.131311000	2.414023000	4.927234000

C	3.280233000	-0.119082000	5.985153000
C	2.987809000	-1.390309000	6.748321000
H	5.463966000	3.765493000	3.855081000
H	1.087316000	3.947614000	2.191046000
H	3.830813000	-1.631104000	7.405196000
C	0.753045000	4.148986000	1.167385000
H	1.510993000	7.924283000	-1.919325000
H	3.792996000	4.331978000	3.967298000
H	2.833994000	-2.256353000	6.093899000
C	1.511128000	5.486813000	-0.672993000
C	4.539356000	0.053000000	5.395613000
C	4.950551000	1.265338000	4.824809000
H	0.177145000	3.271570000	0.836942000
C	2.250327000	7.266616000	-2.383085000
H	1.898764000	7.034983000	-3.394955000
C	1.888295000	4.443536000	0.208539000
C	2.473403000	6.029194000	-1.546449000
H	7.511748000	2.187856000	5.535940000
H	5.234978000	-0.783752000	5.403125000
C	6.233351000	1.270288000	4.057005000
H	3.182173000	7.830724000	-2.489227000
C	7.454008000	1.763552000	4.538035000
H	2.827781000	2.844728000	2.086327000
C	3.145177000	3.838772000	0.192569000
C	3.739131000	5.424998000	-1.556057000
C	3.441659000	2.739788000	1.190104000
C	4.099375000	4.343180000	-0.734506000
H	3.222104000	1.740423000	0.784584000
C	6.230188000	0.726981000	2.765038000
H	4.480798000	5.829617000	-2.240308000
C	8.592955000	1.694843000	3.733956000
H	5.313498000	0.324059000	2.349805000
H	9.552665000	2.057118000	4.087211000
H	5.017100000	1.741709000	-0.554877000
H	4.484962000	2.744592000	1.516097000
C	5.496380000	3.836628000	-0.802492000
C	5.804923000	2.475581000	-0.656510000
N	7.323032000	0.682052000	1.978684000
C	8.494126000	1.154527000	2.457049000
C	6.599504000	4.693938000	-0.991654000
H	6.440555000	5.762500000	-1.094238000
N	7.064134000	1.991387000	-0.619834000
H	9.354902000	1.094851000	1.800281000
C	7.895813000	4.186162000	-1.017464000
C	8.103988000	2.826139000	-0.805159000
H	8.748273000	4.840049000	-1.168360000
H	9.098601000	2.395706000	-0.776084000
Pd	7.237063000	0.000004000	0.000003000

Pd	-7.336791000	0.000002000	-0.000001000
C	0.414627000	6.484521000	2.081616000
H	1.491107000	6.642340000	1.951563000
H	-0.076110000	7.452025000	1.956064000
H	0.245375000	6.151774000	3.112645000
H	-1.157433000	5.209746000	1.297148000
C	-1.105088000	7.463031000	-3.388735000
H	-2.110781000	7.811365000	-3.128747000
H	-0.394300000	8.214925000	-3.037412000
H	-1.037929000	7.417367000	-4.481371000
H	0.139100000	5.704480000	-3.112948000
C	-1.105091000	-7.463028000	3.388725000
H	-2.110785000	-7.811360000	3.128737000
H	-0.394304000	-8.214923000	3.037401000
H	-1.037931000	-7.417365000	4.481361000
H	0.139100000	-5.704479000	3.112940000
H	-1.157437000	-5.209732000	-1.297153000
C	0.414621000	-6.484504000	-2.081627000
H	-1.938187000	-7.909658000	-0.820800000
H	-0.076117000	-7.452008000	-1.956079000
H	0.245369000	-6.151753000	-3.112655000

Pd_2(unstable *Z*-1)$_4$

(*S,S*)$_4$
Sum of electronic and thermal free energies: -5952.761853

H	-0.348844000	1.348783000	4.574277000
C	7.466693000	0.914311000	3.988962000
H	8.398676000	0.762554000	4.523189000
C	0.458858000	1.121003000	5.279413000
C	7.337810000	0.447975000	2.686020000
H	8.147746000	-0.070214000	2.185259000
H	2.796967000	0.611481000	4.122419000
C	6.386909000	1.555778000	4.589282000
C	2.793040000	1.619589000	4.532520000
N	6.194489000	0.617319000	1.989655000
H	6.463881000	1.908183000	5.612708000
H	0.018975000	1.169682000	6.281254000
C	1.624992000	2.069016000	5.159858000
C	5.182286000	1.727056000	3.883344000
C	5.146940000	1.237928000	2.570256000
H	0.784374000	0.091244000	5.097873000
C	3.986245000	2.359922000	4.494551000
H	4.258313000	1.363877000	1.961809000

C	1.661455000	3.362132000	5.740749000
C	0.678702000	4.060358000	6.591899000
C	4.042901000	3.632201000	5.112431000
C	2.879177000	4.082388000	5.735098000
C	1.477569000	5.093319000	7.418338000
H	5.008818000	5.554798000	4.936020000
C	5.276726000	4.508984000	5.117344000
C	2.731834000	5.345832000	6.535132000
H	3.615856000	5.587859000	7.133398000
H	6.000595000	4.220094000	4.350768000
H	5.792951000	4.483162000	6.086270000
H	2.550873000	6.207843000	5.874653000
H	-5.008474000	1.713954000	7.231107000
H	-2.550493000	2.231723000	8.250824000
C	-1.477668000	4.119419000	8.000354000
C	-5.276576000	2.368998000	6.395992000
C	-2.731733000	3.221666000	7.804498000
H	-6.000312000	1.830548000	5.778442000
H	-4.258305000	1.081068000	2.130634000
C	-2.879151000	3.115541000	6.312832000
C	-5.147045000	1.676302000	2.308319000
C	-4.042869000	2.780300000	5.621456000
N	-6.194664000	1.459073000	1.486790000
C	-0.678757000	3.880786000	6.699209000
C	-1.661498000	3.461833000	5.681131000
C	-3.986336000	2.838986000	4.208285000
C	-5.182441000	2.600561000	3.361497000
H	-5.793021000	3.234400000	6.832269000
C	-2.793201000	3.223263000	3.574217000
C	-1.625113000	3.562835000	4.267130000
H	0.348548000	3.388114000	3.355172000
H	-3.615804000	3.633727000	8.301101000
C	-6.387207000	3.303035000	3.545553000
C	-7.467075000	3.078306000	2.696336000
C	-0.459000000	4.117155000	3.488810000
H	-2.797216000	3.339766000	2.492139000
H	-0.018895000	4.976250000	4.006313000
H	-6.464216000	4.037061000	4.341032000
H	-8.399172000	3.620399000	2.816138000
H	-0.784584000	4.445268000	2.496007000
H	-8.148103000	-1.956878000	-0.974568000
C	-7.338092000	-2.152320000	-1.668082000
N	-6.194638000	-1.459175000	-1.486858000
C	-7.467026000	-3.078420000	-2.696415000
C	-5.147013000	-1.676392000	-2.308381000
H	-8.399116000	-3.620524000	-2.816221000
H	-4.258280000	-1.081150000	-2.130690000
C	-6.387151000	-3.303137000	-3.545626000

C	-5.182394000	-2.600648000	-3.361564000
H	-2.797191000	-3.339870000	-2.492174000
H	-6.464146000	-4.037164000	-4.341105000
N	6.194521000	-0.617239000	-1.989625000
C	-2.793155000	-3.223355000	-3.574249000
C	-3.986276000	-2.839065000	-4.208336000
H	4.258357000	-1.363830000	-1.961796000
C	5.146986000	-1.237864000	-2.570235000
H	8.147769000	0.070324000	-2.185212000
H	0.348590000	-3.388209000	-3.355136000
C	7.337845000	-0.447877000	-2.685982000
H	-0.348785000	-1.348848000	-4.574216000
H	-0.784564000	-4.445372000	-2.496012000
H	-6.000204000	-1.830566000	-5.778520000
C	-0.458956000	-4.117246000	-3.488804000
H	2.797001000	-0.611469000	-4.122384000
C	-1.625052000	-3.562918000	-4.267144000
C	-4.042777000	-2.780349000	-5.621507000
C	0.458898000	-1.121049000	-5.279367000
C	-5.276460000	-2.369009000	-6.396064000
H	0.784389000	-0.091279000	-5.097837000
C	2.793101000	-1.619574000	-4.532495000
C	5.182350000	-1.726989000	-3.883325000
C	7.466744000	-0.914206000	-3.988924000
H	-0.018842000	-4.976335000	-4.006309000
C	1.625060000	-2.069029000	-5.159829000
H	0.018999000	-1.169745000	-6.281200000
H	-5.008327000	-1.713947000	-7.231156000
C	-1.661405000	-3.461885000	-5.681145000
H	-5.792905000	-3.234393000	-6.832378000
C	-2.879042000	-3.115574000	-6.312863000
C	3.986325000	-2.359875000	-4.494540000
C	6.386974000	-1.555687000	-4.589255000
H	8.398729000	-0.762433000	-4.523143000
C	1.661553000	-3.362139000	-5.740729000
C	4.043012000	-3.632147000	-5.112434000
C	-0.678642000	-3.880814000	-6.699211000
C	-2.731587000	-3.221648000	-7.804529000
H	6.463960000	-1.908090000	-5.612681000
C	2.879296000	-4.082359000	-5.735098000
C	0.678816000	-4.060377000	-6.591890000
H	-2.550342000	-2.231687000	-8.250814000
H	6.000727000	-4.219990000	-4.350788000
H	-3.615642000	-3.633694000	-8.301169000
C	5.276860000	-4.508895000	-5.117363000
C	-1.477510000	-4.119386000	-8.000388000
H	5.008982000	-5.554718000	-4.936045000
C	2.731983000	-5.345798000	-6.535145000

H	2.551048000	-6.207818000	-5.874671000
C	1.477703000	-5.093316000	-7.418340000
H	5.793078000	-4.483051000	-6.086293000
H	3.616005000	-5.587799000	-7.133419000
H	5.008928000	-4.935978000	5.554769000
H	2.550994000	-5.874650000	6.207838000
C	5.276824000	-5.117301000	4.508952000
H	6.000678000	-4.350713000	4.220049000
H	5.793063000	-6.086220000	4.483124000
H	3.615997000	-7.133375000	5.587852000
C	2.731962000	-6.535128000	5.345828000
C	4.042989000	-5.112407000	3.632184000
C	1.477712000	-7.418359000	5.093325000
C	2.879280000	-5.735094000	4.082381000
H	6.463962000	-5.612646000	1.908172000
H	8.398740000	-4.523101000	0.762538000
C	6.386972000	-4.589225000	1.555759000
C	7.466747000	-3.988890000	0.914291000
H	-0.018830000	-4.006369000	4.976313000
H	-0.784512000	-2.496063000	4.445322000
C	0.678821000	-6.591937000	4.060366000
C	3.986311000	-4.494529000	2.359906000
C	5.182338000	-3.883304000	1.727033000
C	7.337844000	-2.685951000	0.447951000
C	-0.458912000	-3.488861000	4.117209000
C	1.661554000	-5.740771000	3.362134000
C	-1.477543000	-8.000415000	4.119389000
C	-0.678638000	-6.699260000	3.880790000
H	8.147774000	-2.185178000	-0.070237000
C	5.146972000	-2.570217000	1.237904000
N	6.194512000	-1.989602000	0.617293000
H	0.348656000	-3.355211000	3.388194000
C	-1.661379000	-5.681185000	3.461831000
C	-1.625005000	-4.267183000	3.562849000
C	2.793102000	-4.532521000	1.619581000
C	1.625069000	-5.159883000	2.069018000
H	4.258337000	-1.961783000	1.363856000
C	-2.731593000	-7.804555000	3.221617000
H	-3.615667000	-8.301171000	3.633654000
C	-2.879020000	-6.312889000	3.115511000
C	-2.793094000	-3.574275000	3.223267000
H	8.147738000	2.185288000	0.070291000
H	-2.797121000	-2.492199000	3.339791000
H	2.797013000	-4.122424000	0.611472000
C	7.337805000	2.686048000	-0.447905000
C	0.458928000	-5.279468000	1.121016000
H	8.398669000	4.523221000	-0.762468000
C	7.466688000	3.988992000	-0.914235000

H	-2.550333000	-8.250865000	2.231669000
C	-4.042739000	-5.621519000	2.780264000
N	6.194488000	1.989679000	-0.617261000
C	-3.986219000	-4.208349000	2.838968000
H	0.019074000	-6.281321000	1.169698000
H	-0.348792000	-4.574356000	1.348805000
H	-5.792886000	-6.832351000	3.234335000
C	5.146942000	2.570279000	-1.237878000
C	6.386909000	4.589310000	-1.555710000
H	0.784367000	5.097899000	-0.091225000
C	-5.276437000	-6.396060000	2.368942000
H	-6.464080000	-4.341138000	4.037068000
C	-5.182335000	-3.361571000	2.600561000
H	2.796958000	4.122430000	-0.611452000
H	0.784429000	-5.097919000	0.091254000
C	-6.387091000	-3.545652000	3.303047000
C	5.182290000	3.883369000	-1.727003000
H	6.463882000	5.612737000	-1.908112000
H	4.258318000	1.961829000	-1.363839000
C	2.793042000	4.532537000	-1.619559000
C	-5.146965000	-2.308387000	1.676306000
C	0.458862000	5.279450000	-1.120986000
H	-5.008323000	-7.231166000	1.713892000
H	-4.258237000	-2.130689000	1.081058000
C	-7.466974000	-2.696449000	3.078337000
C	3.986255000	4.494576000	-2.359878000
H	-6.000172000	-5.778510000	1.830493000
H	-0.348846000	4.574325000	-1.348777000
C	1.625001000	5.159886000	-2.068991000
H	-8.399062000	-2.816269000	3.620441000
H	0.018991000	6.281296000	-1.169661000
N	-6.194601000	-1.486875000	1.459094000
C	-7.338051000	-1.668113000	2.152242000
C	4.042929000	5.112475000	-3.632148000
H	-4.258262000	2.130630000	-1.081088000
C	1.661485000	5.740798000	-3.362095000
H	-8.148070000	-0.974608000	1.956801000
H	6.000632000	4.350826000	-4.220025000
N	-6.194605000	1.486772000	-1.459160000
C	-5.146984000	2.308309000	-1.676353000
C	5.276767000	5.117406000	-4.508913000
C	2.879214000	5.735157000	-4.082337000
H	5.792990000	6.086333000	-4.483064000
H	-5.008471000	7.231093000	-1.713959000
H	-2.550468000	8.250845000	-2.231673000
H	0.348662000	3.355288000	-3.388063000
C	-7.338046000	1.667984000	-2.152328000
C	0.678747000	6.591972000	-4.060312000

C	-5.182361000	3.361492000	-2.600607000
H	-8.148054000	0.974461000	-1.956902000
C	-3.986260000	4.208297000	-2.838993000
H	-6.000279000	5.778410000	-1.830583000
C	-2.793108000	3.574248000	-3.223253000
C	-5.276546000	6.395976000	-2.369014000
C	-4.042817000	5.621467000	-2.780299000
C	-1.625027000	4.267183000	-3.562808000
C	1.477634000	7.418436000	-5.093240000
C	-1.661442000	5.681182000	-3.461806000
C	-2.879105000	6.312863000	-3.115518000
C	2.731889000	6.535219000	-5.345766000
H	-2.797104000	2.492171000	-3.339762000
C	-0.678713000	6.699273000	-3.880748000
C	-0.458886000	3.488891000	-4.117110000
H	3.615918000	7.133479000	-5.587779000
C	-2.731702000	7.804533000	-3.221624000
H	5.008874000	4.936101000	-5.554733000
C	-7.466975000	2.696319000	-3.078426000
C	-6.387107000	3.545545000	-3.303116000
H	-0.018792000	4.006397000	-4.976209000
C	-1.477634000	8.000412000	-4.119369000
H	-5.792983000	6.832250000	-3.234423000
H	-3.615774000	8.301135000	-3.633679000
H	-8.399057000	2.816119000	-3.620546000
H	-0.784437000	2.496074000	-4.445214000
H	2.550920000	5.874759000	-6.207789000
H	-6.464100000	4.341031000	-4.037136000
Pd	-6.140976000	-0.000051000	-0.000033000
Pd	6.140877000	0.000038000	0.000015000
C	-7.338126000	2.152205000	1.668006000
H	-8.148131000	1.956752000	0.974488000
C	-0.776810000	-9.321079000	3.776847000
H	0.022928000	-9.584839000	4.470591000
H	-0.352168000	-9.284204000	2.766764000
H	-1.505191000	-10.139179000	3.803174000
H	-1.826804000	-8.031371000	5.161125000
C	0.777014000	-7.741846000	6.418812000
H	1.505328000	-8.152716000	7.126812000
H	-0.023011000	-8.477478000	6.322694000
H	0.352746000	-6.834592000	6.864722000
H	1.826690000	-8.350669000	4.627302000
C	-0.776946000	3.776882000	9.321026000
H	0.022813000	4.470609000	9.584770000
H	-0.352332000	2.766786000	9.284170000
H	-1.505326000	3.803247000	10.139125000
H	-1.826906000	5.161164000	8.031295000
C	0.776846000	6.418795000	7.741811000

H	1.505136000	7.126800000	8.152715000
H	-0.023207000	6.322659000	8.477413000
H	0.352605000	6.864708000	6.834547000
H	1.826529000	4.627297000	8.350658000
C	-0.776917000	9.321084000	-3.776820000
H	0.022849000	9.584832000	-4.470539000
H	-0.352312000	9.284224000	-2.766721000
H	-1.505298000	10.139182000	-3.803188000
H	-1.826869000	8.031359000	-5.161113000
C	0.776938000	7.741966000	-6.418717000
H	1.505249000	8.152876000	-7.126697000
H	-0.023099000	8.477583000	-6.322572000
H	0.352685000	6.834724000	-6.864665000
H	1.826604000	8.350734000	-4.627183000
C	-0.776735000	-3.776771000	-9.321012000
H	0.023052000	-4.470466000	-9.584755000
H	-0.352143000	-2.766668000	-9.284088000
H	-1.505078000	-3.803109000	-10.139146000
H	-1.826734000	-5.161133000	-8.031390000
C	0.777026000	-6.418820000	-7.741802000
H	1.505343000	-7.126799000	-8.152705000
H	-0.023032000	-6.322721000	-8.477401000
H	0.352808000	-6.864745000	-6.834533000
H	1.826638000	-4.627279000	-8.350661000
